# Correction: Perceptions of persons deprived of liberty regarding tuberculosis vaccine research

**DOI:** 10.1371/journal.pgph.0005922

**Published:** 2026-01-30

**Authors:** The PLOS Global Public Health Staff

The first two authors, Mariana Cristina Campos Falleiros Pires and Yiran E. Liu should be noted as shared first co-authors on this work.

The publisher apologizes for the error.
