# Supplementary material for: Perceptions of persons deprived of liberty regarding tuberculosis vaccine research
Source: PLOS Glob Public Health. 2025 Dec 16;5(12):e0004941. doi: 10.1371/journal.pgph.0004941 (PMC12707645; doi:10.1371/journal.pgph.0004941)
Supplement: S1 Data — (DOCX) [file pgph.0004941.s001.docx]

**S1 Data: Complete Dataset Supporting the Study Findings**

This dataset consists of verbatim statements from participants, which have been subjected to a thematic coding process. It is important to note that the same statement may be relevant and, therefore, simultaneously classified in more than one coding category. This approach aims to capture the complexity and interconnectedness of the themes addressed by incarcerated individuals, ensuring that the richness of their perceptions about health, autonomy, risk, and trust is fully preserved in the sub-codings.

**Health Care in Prison**

- Access to health services
- Distrust in the prison system
- Equity in health services
- Neglect of rights
- Neglect/deficiency of health care

This document presents the participants' literal quotes, which constitute the database for the critical analysis of Health Care in Prison. The following transcripts detail the perceptions and experiences of individuals deprived of liberty (IDLs) regarding Access to health services and Equity in health services. The discourses reflect a deep Distrust in the prison system and health management, addressing the Neglect of rights and the various instances of Neglect/deficiency of health care observed in the routine of prison units.

**Discourse:** I've been a health worker here for twelve years, even at the previous penitentiary. And the treatment has always been very adequate, everything. I've lived with people who had it, but they were taken care of. But you can't just isolate everyone. So prevention is done, everything. Everything is always handled correctly. The health care here is excellent. It always has been, you understand? Everyone is well taken care of. The hospitalizations, everything. It's always been handled correctly. And regarding those who come from the outside, because they're already being treated, right? They just continue their treatment here inside. And here, the area of care regarding health, regarding TB, has extreme control, right? No... There's never been a single case of anyone dying from TB here. Everything is well handled, well organized. Everyone receives their medication, the tests, everything is done directly. Everyone who has a health problem, that we notice, is immediately referred to where they can get tested and start treatment. So that there isn't that time for it to accumulate, right? I've been a community health worker here for 12 years. And the treatment here has always been very good. From TB nursing to other situations. The facility is always well-organized, right? And the care is always excellent, right? There's nothing to complain about. Because I've been around for 12 years, haven't I? One, two days, a year, two... But it's been a while. If there was something wrong, we would say so. No, it's excellent. There's nothing to complain about, right? The facility, the care, the nursing, everything. Nobody goes without medication in any area. They are always well cared for, well assisted. Psychologist, psychiatrist, everything, you understand? When it's reported and confirmed that it's TB, the infirmary is called. They talk to the psychologist, everything. And the treatment starts right there, you understand? The treatment is given at the door. The nurse comes, pays for the treatment. Everything is done. There's nothing... It's all within the correct requirements, right? Because it's a penitentiary, right? There's no specific place to leave them, right? Go on, you have to know how to live. You have to isolate the cell there. Those who are in the gallery, isolate that cell. And make sure the others don't get it. Up to 10 days there, and then release them.

**Discourse:** This year... This year, actually, since I've been here for two years, nothing has been achieved. Last year we did achieve our goals; we distributed pamphlets and even put them up, dividing them into two galleries, A and B, right? We put them on the wall so people could see the level of contamination, all the things, the duties, the precautions, right? They achieved their goals, they have the pamphlets, we put them up, right? But this year, unfortunately, that wasn't done, right? Therefore, even our meetings with the health agents, this year is the first. If the guy there has been here for 12 years, correct him, it's the first, right?

**Discourse:** Correction. There was one in January. There was one in January.

**Discourse:** I've been here for a year and nine months, but I haven't been called.

**Discourse:** I've been here for a year and two months, I wasn't invited to this meeting, was I? Suddenly, everyone is speaking for their own group.

**Discourse:** But here, number nine, but here the treatment, nobody... Tell me about it, nobody can complain. The nurse goes to the door, gives the treatment, sees the prisoner receive the treatment, and only leaves after the prisoner has finished. That's the reality.

**Discourse:** The only problem, which I don't know if it's suddenly... It's not the prison's fault either, I think it's just that... It must be from outside, something that he... Sometimes there are prisoners who take three, four months to find out... I don't know if it's from the disease or not, right? They find out, they go there and take the test, and they can't find out if it's tuberculosis. There was one there who... In the gallery where I am, who... He, I think on the fourth, third or fourth time he took the test, he found out he had tuberculosis. But, you know, I don't know if it's suddenly from the disease, sometimes it takes longer to manifest, or something like that, but about the prison, the prison provides the right treatments, the tests are done when requested. That can't be denied.

**Discourse:** But the health workers don't give them out, the nurses don't give them masks. So, the on-call staff also talks to them. They have to wear them there, it's very... Regarding medication, like he said, I have a little box there with all the medicines. You can take 10 to the ward per week. I take five, four. The rest I can control internally. I go twice a week, I see who is losing weight, who has symptoms of tuberculosis. Then, I put them there, I take them to the nurse on Monday. Look, we can do sputum tests on these people. They have sweats, they have a fever, so we don't have a tuberculosis outbreak in the ward, in this part, there's nothing to say. I've always been like that, you know. As much as possible, you know. As much as possible, also, because there are several wards. You can't just say there, oh, let's go to this ward, but let's put the 20 that are there. So, we put them as we can, you know. So we understand this, too. One guy was there, he got tested, but it didn't show up. He got tested again, it was drying up, it was really strong, it wasn't drying up. I insisted, no, it's impossible. He went there, they even tested him, we isolated his cell. And then, we went to see the others, taking extra special care. Just to see the others, you know. To see if they were going to show any symptoms so we could catch them early and test them.

**Discourse:** This last vaccine had several side effects lasting two to three days. Fever, body aches, and sweating. It was a COVID booster. Some vaccines have side effects. Several people... This should be optional, right? It should be optional, not mandatory. You understand? Signing the consent form is fine. If there's a problem, you go there and... You understand? Not that everything is mandatory. One thing you do... There are people who don't even have more problems. The effect of the other one. Not every body is the same. Each body is different. Each person has a body, a blood system, blood type. Everything is different. When you take a series of people, everyone is different. It's like a fingerprint, everyone is different. No two are alike.

**Discourse:** The unit has support from the technical staff, which are the doctors, and sometimes they are meeting all the demands and needs of the interns, but fortunately there are times when they can't meet all the demands, also because there are many interns. But regarding medication, we are quite well assisted, you understand? But there's a lack of attention for cases that happen within the unit, like today we're doing tuberculosis research. Many times, those who have tuberculosis, like me, are undergoing treatment, and sometimes we aren't; the medication doesn't go down at the right time, you understand? We have to take the medication at the right time, right? For it to be effective. So that's all from my side.

**Discourse:** Yes. Sometimes, as I'm saying, there's a problem where it doesn't come through; one time comes, then another time comes, and our bodies don't balance things out properly, right?

**Discourse:** I was hospitalized in this unit when I contracted tuberculosis. I was hospitalized for six months, for 15 days, and the situation where we are treated here for this disease is not adequate. The medical staff needs to pay more attention to us, the medications, everything on time. There isn't enough treatment here in this unit where they put us, to be treated sufficiently, at the right time, and we get even sicker. I believe they should release us to a hospital that is more suitable for our situation, because you know that our breathing is difficult, we could even die here in this place.

**Discourse:** He's kept separate, isolated, far from the others because of the risk of contamination. We're still, let's say, I don't know how to say this word, other people look at you and say, "Oh, that one got tuberculosis, stay close to him, no," and it becomes embarrassing for us, doesn't it? We're living in the same cell, which is a small cell, and the air is constantly circulating, so we can't be in the same place. And there's no suitable place here for us to be hospitalized in a situation like this, there isn't, we get short of breath, he could even die in here.

**Discourse:** There's a lot, yeah, because, you know, we're isolated, like the brother said, then the 15 days pass, which is supposed to be the contamination period, right, as they say. Then, during those 15 days that we're there, we're at the mercy of the situation, like he said, because we're not being assisted. Like, I was there too, I spent 24 days there, when it was supposed to be 15. Then it took a while for the other container to arrive to do the test, so we're in a cell, alone, isolated, next to the infirmary, which is also the infirmary, but we don't get the proper treatment. They isolate us from our cell, we're getting more care from the others, they put us near them so we can be cared for by them, and we don't really get that care, you know? We're at the mercy of the situation.

**Discourse:** So, I'm asking for a little more attention for the brothers and sisters who, well, there are brothers and sisters who get this disease, you understand? Because the place they put us in isn't suitable for us, because they put us in the ward. If you take a look there, when you go in, you have to walk like this, because it's a mosquito infestation, you understand? And there we are, mostly sick and hospitalized in that place, you understand? Because we don't have the attention, the care, the medication, because it was the first time I've ever had this situation in my life, you understand? But when I went to be treated in the ward, I saw that it was already painful to be hospitalized in that place, isolated there with the mosquitoes, without the technical nursing support that we should have, you understand? So, we are somewhat, like, discarded, like, when we are in this unpleasant situation in our lives.

**Discourse:** I came from the streets with this disease, you understand? Tuberculosis. I came straight to prison, from there I went straight to isolation, they didn't send me to the general population, you understand? I spent forty-eight days in that infirmary, and in the infirmary we need a lot of attention, you understand? Because of this disease, something that wasn't happening there. The brother who went through there knows all the things that were happening, and we need a lot of attention. There's medication to be paid for at the right time, I pay two hours later, sometimes I pay after lunch, you understand? Even though we're supposed to take it before, and this disease is very serious, you understand? We need a lot of attention, and since they put us there, they have to give us attention, you understand? Because it's next to the infirmary, and in the case of the medication schedule, it always has to be at the right time, right? So that's what I have to say here, and please give a little more attention to those of us who have this disease, you understand? We're still not one hundred percent well in treatment, you understand? We need to get these doctors' attention so they can pay for the medication on time, you know?

**Discourse:** When I broke my leg in prison, I was put together with those who had tuberculosis, you understand? Because the prison system doesn't differentiate if you have a broken arm, a broken leg, if you have AIDS, if you have tuberculosis, it doesn't differentiate. They throw you all together because they can't meet everyone's needs. There, you understand? The system can't accommodate everyone, each one with the correct treatment, you understand? So, I spent seven months there treating my broken leg. I didn't have tuberculosis at all, but I was living with people who had tuberculosis right next to me. When I went out, I came back, I caught tuberculosis, on the street, on the street. When I got back inside the prison system, I notified the staff. I'm undergoing tuberculosis treatment, can you send me medication? They didn't pay attention. When I arrived at this unit here, two months had already passed, they went to the SUS (Brazilian public health system), they saw that I had already been marked as having given up. So I started the treatment again from the beginning. So, I spent five months out there, and I've already started the six months again. For what? I spent eleven months taking this medication. And for a brother who has tuberculosis today, who doesn't know he has tuberculosis today, he'll only find out if he gets a fever, if he really needs it, even if you see our whole appearance changes. We get pale, we get a high fever, there's a tremor in the body, loss of appetite. And we don't have daily access to testing here. We don't have that. We ask and the nurse will see if you really need it, but she doesn't know what we're feeling at that moment. Then yes, there will be testing, but not for all the cells, it's only for one person. If one person in the cell has tuberculosis, which was my case, everyone will have to take the test. Then yes, you understand? But the others who have it and don't know, whose condition remains incubated, won't take this test, you understand? So, I think the prison system needs to improve its technical staff, I mean, with more nurses, with more assistance from technicians to be able to take care of each one of us.

**Discourse:** Good morning, actually when they come to vaccinate us here in prison, they don't inform us, they don't inform us. They call us and the vaccine isn't just a simple way of saying, "Look, there will be a vaccination for you tomorrow for this, that, and the other thing." At a certain point, they arrive at the prison, call the cell block, say they're going to get vaccinated, and they don't explain the situation with the vaccine. They say it's for the flu, they say it's for Covid, and for other diseases we don't know about. We have no way of knowing...

**Discourse:** I think the staff are there, right? The nurses have to help us, but I think the proper medications aren't arriving to meet our needs, to be able to meet them when we have them. I believe that's what's happening in the unit. I think the medication people need isn't arriving because, lately, even people who depend on sleeping pills like clonazepam and cyclobenzeprine aren't coming here anymore. And other medications, I think they're not arriving at the unit for us to receive. I don't have complaints about the staff's service, you see? And even then, it takes a while to get the patient out, and then they come and get them. So I think it's a difficulty in getting the materials to us... to help us consume them, to improve our condition, to deal with any illness we might have in this chaotic situation, which we thought would happen, yes. That's my opinion.

**Discourse:** Yes, wait a minute. Regarding the attention from the unit's staff, sometimes we encounter a lack of assistance within the unit, a serious situation, sometimes with shortness of breath, sometimes with a colleague whose condition could worsen, and we have to wait an hour or more to be seen, that is, when the infirmary is open and there are staff to come to the unit. When we get there, there's a lack of medication, there's no medication, sometimes we are poorly treated, sometimes we wait a long time to be seen in the afternoon. When someone suffers from a more serious illness or becomes ill, there's a lack of medication or an intestinal infection, because of the poor quality of the food we are given, we wait until late at night to be seen. And even then, when we leave, it's under threat, when we have to go to a hospital, it's under threat. I think they should pay more attention to our health situation here, because it's not just us, but if it weren't for us, we would have many prisoners.

**Discourse:** So, when I found out I had tuberculosis, I had to say I had it. I went to the infirmary because they had already told me I had tuberculosis. I went up to the infirmary and told them I had tuberculosis. The nurse said to me: "How do you know you have tuberculosis? Because of the symptoms, I can't eat and I'm getting weak, I have a dry cough and so on." I explained the symptoms, and she said it didn't mean anything. Then I asked if I could get tested, and the nurse told me they weren't doing the test and that it wasn't done that way, she had to schedule it. She couldn't schedule it after a month, but after a month, she scheduled it, but I was already coughing up blood, and even then she didn't believe me. She sent me back to my cell, and I waited fifteen days for the test results to arrive. When they arrived, I really did have it. That's how they found out.

**Discourse:** Honestly, there's a lot of negligence on the part of some of the staff. Some try to help us, but others, for them, it doesn't matter; they just want to come, do their day, and leave. I myself have been without food for four or five days, with a terrible sore throat, body aches, fever, a lump in my throat, difficulty swallowing, even saliva and a glass of water—I don't even enjoy drinking water, I can't sleep. So, I've already asked several staff members, I'm even speaking rather loudly because if I speak softly, you won't be able to hear. So, it's already hurting on both sides, there's a lot of plaque, a high fever, at night, sweating, feeling very ill. I've already asked for various medications, but they don't reach me. There's negligence on the part of some staff members, but some don't care or pay attention; it's only here. For them, we're just prisoners; they just want to do their job. For them, it doesn't matter if we close, it's all the same, they lock us up. Since I don't have visitors, I have no way to communicate with my family, so it becomes somewhat impossible for me.

**Discourse:** The infirmary is very good and they provide excellent care, but the difficult part is getting there, to the infirmary, leaving the cell; that's the hardest part for the prisoner. The infirmary here isn't so bad, in my opinion.

**Discourse:**: Everything in life has two sides. Today we live in a precarious system, but why don't they pay much attention to the prisoners inside? Because many have nothing and want to go out for medical attention, so every day, one prisoner tries to harm another, you know? Because they want to have a good time in the prison, and they end up harming those who are really sick. Then, they end up closing the door to those who are truly ill. But there are two sides to it.

**Discourse:** I'm undergoing treatment myself; I have to take anxiety medication to sleep at night. I've asked several times, and they don't pay any attention at all, so I gave up and don't ask anymore. The infirmary has to be sent to the person in charge of restocking because, here in the system, we call it a cage, but it doesn't reach our hands. Sometimes it does arrive; you ask a staff member, and they kindly give it to us. Some don't have it, it's stuck, unfortunately it hasn't reached us. Some also make a little effort, but they don't pay attention; it's always like that. It's like the guy over there said, the infirmary has the medication, the difficult part is getting it there. I believe that if I had family, someone here who could get it to the attention of a higher-ranking coordinator, I would get it to the infirmary immediately because this is a serious case; it can cause a lot of problems. You understand?

**Discourse:** The only drug we use here is just drugs, pharmaceuticals, chemicals. For six days I'm up all night, without any pressure, just inside the cell.

**Discourse:** I have it. I think I even caught it. I'm dying little by little, I've been here for three years, I'm losing weight, I've lost about 20 kilos.

**Discourse:** No, I did it in the middle of last year. Last year? I did it and nothing happened. I was depressed... Depressed.

**Discourse:** It was God, it was God who saved him from getting caught. That's my thought.

**Discourse:**. High immunity.

**Discourse:** It was God who helped him.

**Discourse:** High immunity.

**Discourse:** Yes, normal, common.

**Discourse:** No.

**Discourse:** Man, this is the first time in my life that I've seen anything like this in prison.

**Discourse:** No, just the talking. I only hear about it.

**Discourse:** I don't know anyone in this unit, I'm 32 years old and I've been here for 6 years.

**Discourse:** In another prison I was in, two people died, not in the same cell, but in another one.

**Discourse:** Last year I was in the "Luta Ladeira" prison, there was a huge tuberculosis outbreak there, that's where I lived in that cell with prisoners, and then I was transferred to another wing, I arrived in another wing, there was already another prisoner with tuberculosis, he was also going to the infirmary a lot. I didn't witness any deaths, but I spent a lot of time with people like that.

**Discourse:** Healthcare here is something that's very scarce. You understand? Their service here is slow. Sometimes they don't have the necessary medicine to determine the type of occurrence, certain illnesses that happen during the cell. They prescribe a medicine, let's say, that won't solve the problem, it will only alleviate it. What they have most of is ointment and dipyrone. Everything else is in short supply. For boils, for example, there were times when they ran out of medicine. They have tuberculosis, there was plenty of it in the unit. Now, for other treatments, other illnesses, sometimes it's quite complicated, there's a lot of shortage.

**Discourse:** I think the facility is very underfunded. Not only in terms of medication, but also in medical care and escorts. People sometimes die. Things that could be saved. So I think that minute by minute we know that our breath is very valuable and if it arrives in time, help is provided. But most of the time it doesn't happen. But not because of the facility. The facility chases after these health escorts, these services. The facility does what it can, but it's the system, the outside world, especially the health system. So I think in that sense it's very weak.

**Discourse:** This prison health strategy is weak, very weak. Even in our cell there was a man with a terrible flu. It leaves you waiting a long time. But I'm surprised that they even had medicine for TB.

**Discourse:** You've been waiting a long time, a year waiting for an appointment with a neurofibromatologist. So that's your only concern.

**Discourse:** And sometimes, not always, there's a specific medicine for us, for example, if we have a fever, a headache. Then she gives us Benegripe, which isn't... I think it's irrelevant, right? So, she always gives a medicine that isn't specific to what we're feeling. You understand? In my case, I have gallstones. But thank God we're lucky because we 're getting our tests done through the SUS (Brazilian public health system), it took a while but they're finally here, it's a short time. But when we're having a crisis, they only give omeprazole and Buscopan compound, that's it, there's nothing else. So we have to accept it, we have to endure the pain, because the medicine they're giving isn't specific to what we're feeling. So, it's a lack of medicine. There's also a lot of itching here in the system, we feel a lot of itching, I don't know if it's from the mattress, I don't know, they even give us a lotion, when they have it? Lotion for us to apply to the body, right? And a little pill sometimes helps too, it soothes, right? So, and in the case of others, we also ask to go to a specific doctor, but it takes a long time, the escort in that case, right?

**Discourse:** But she never investigates our problem, does she? Because whenever we have something, she always says no, right? What you don't need, simply, if you prescribe medication, I think most of us here have already been examined by her, right? And she always says, we don't have anything.

**Discourse:** Then the escort takes more than a year, because we arrange for the escort to go to the doctor. Meanwhile, we're left suffering with the illness. I give them some medicine to alleviate it, you know? And even that doesn't really help.

**Discourse:** Everyone here has a tumor, practically. A lot of tumors grow in us.
 A lot, a lot, a lot in here.

**Discourse:** Just so the professional can tell me it's a bacteria and give me penicillin but it never gets better.

**Discourse:** During the COVID-19 pandemic, they obtained permission to receive treatment outside the prison system because they often don't receive adequate treatment within it. To avoid consequences like death, they often create a document, send it to the judge, and release these individuals for treatment.

**Discourse:** I've been trying to get a blood test for a year. Zero.

**Discourse:**  Every pain is dipyrone. Fell out of bed, dipyrone and ice.

**Discourse:** Actually, it's not even dipyrone anymore, it's paracetamol. I'm being treated for diabetes and I'm having a problem with my blood sugar, so it's having spikes, either dropping too low or rising too high. And they simply tell me: "Don't give me insulin, you have to wait for the doctor." And what if, in the meantime, since my diabetes is chronic, I have a flare-up? What if I have a spike? That's just my experience. There are other women who have terrible treatment. Sometimes they need insulin and there's no insulin available. I was bedridden for seven days because I didn't have insulin; my wife had to buy it. And it takes a week to get through to family members so they can send medication. So it's awful.

**Discourse:** I had breast cancer at 25, I had my entire right breast removed, and about a year and five or six months ago my breast became infected. It was swollen, red, and had discharge. I tried and tried to get treatment and couldn't. Then one day I saw a lady from the department and showed her my breast and said: "Look at the situation I'm in, and I'm trying to get treatment and I can't." She said: "Wow, it's very red, with a fever." Then she said she would see what she could do, so she requested and got an escort and I had a breast ultrasound, but the doctor doesn't talk to us. So I came back and it disinfected itself. Now, about a year and six months ago, I went to the pharmacy to get the medication my family sends, and the doctor saw me and said: "Oh Micheli, I was looking at your exams and they showed six nodules, but according to the parameters they are normal." Then I said, "But how can they be normal, doctor? I had cancer." She said, "In the other breast that I didn't have removed, oh, then I was worried, I'll try to schedule a mammogram for you." They requested a follow-up every six months, but now it's been a year and six months and they've finally checked.

**Discourse:** Another thing that happens a lot, and happened to me, is people falling out of bed. Since there's no safety railing, it's normal for people to fall, and the service is terrible.

**Discourse:** You fall, fracture something, and there's no immediate escort to take you to the hospital for treatment. Often, the security forces refuse to take you at that moment. When it happened to me, I realized I was fine at the time, I hadn't fractured anything, I got up, but then I started feeling a lot of pain in my lower back, and it was a struggle, more than a month, to get treatment, an injection to treat the pain.

**Discourse:** Excuse me, we have a colleague who fell off the "Jelga." She fell onto the bed—the "Jelga" here is a bed—and she fell headfirst onto the floor. She started bleeding from her nose, ears, and even her eyes. She had, what's the name? She had a traumatic brain injury and a stroke on the side of her face, which required extensive treatment. And she should still be going back there, and she has a lot of headaches. She cries from the headache. She cries, she cries from the pain.

**Discourse:**They saw blood, right? And I think they found it... And the shift, it was a shift that treats us like human beings.

**Discourse:** The bed is very high. And it doesn't have a... It doesn't have a guardrail. It doesn't have a door, it doesn't have a guardrail.

**Discourse:** And depending on the time, there's no escort. If you fall at night, if you get hurt, they tell you to stay lying down, because they don't even open the... Because there's a little window on the door that opens to talk to us. They don't even open the little window, they tell everyone, leave her alone, don't touch her, that tomorrow we'll see what happens.

**Discourse:** There are accommodations that have 36 people. There are only 18 beds.

**Discourse:** Overcrowded, it's practically double the number of people.

**Discourse:**There are 18 beds, but there have been as many as fifty-two people.

**Discourse:**You can't even go downstairs to use the bathroom. You have to step
over others and if you complain they get angry.

**Discourse:** And I spent 7 months fighting to be seen by a doctor because I have ovarian cancer, because my belly started growing and I had cancer. I had surgery 12 days ago, which is why I'm having cancer removal surgery. I'm going to start chemotherapy, I should have been doing it since February, since January, I'm starting now because of the delay at home. Because it doesn't move, I have to go out with an escort, they don't send people to appointments, there's no one to send them. And the longer it takes, what will happen? My cancer has already come back again, the cancer has already returned to my body, and I can't do chemotherapy to kill it. Because the type of cancer I have is one that, one day it moves and then another one appears. So chemotherapy is necessary, and in this process, I spent 7 months fighting. My belly got this big, I would say I succeeded, but I still haven't succeeded because I was with one of the incarcerated girls who passed the legal process, I had to go with an escort. Because he kept saying it was just poop, that it was gas, so sometimes I'd say there was nothing I could do for myself, and my belly was getting bigger every day, I couldn't sleep, I was in pain, and I couldn't even speak, and they wouldn't let me see the doctor.

**Discourse:** I have problems with asthma, for example, and it 's difficult to get medication. Before, when I asked the nursing home, they always gave me the inhaler, but now they don't even have that. There are people here who can't afford to buy it.

**Discourse:** And many people are still feeling unwell, and I can't afford to buy an asthma inhaler. There are many here who also can't afford the medications they prescribe.

**Discourse:** So, if it's a good shift, like I said, that treats us like human beings, they'll even go and ask you to pass the medication to a certain ward because the girl is feeling unwell, she's in pain, paracetamol,ibuprofen, dipyrone, and sometimes if it's a good shift they authorize it, the pharmacy comes, picks it up and takes it, but if not, they don't authorize it, they say no, they won't pass it and leave.

**Discourse:** As I just told her, I'm diabetic too, I'm 24 years old and I have hereditary diabetes. I started taking insulin inside this prison. I didn't see any endocrinologist. There were times when my levels dropped too low and I felt tingling, drowsiness, things like that. I got to the infirmary, talked to the nurse to see the doctor, and when I finally got to speak to the doctor, I said, "I'll just give you a consultation," she scheduled it, said he denied it, then she scheduled me again, said he denied it again, but I didn't get the appointment. She wanted to give me four insulin doses a day, I take two, she wanted me to take four, because when we ask, we can measure it to check the dosage, but there's no lancet, no needle to prick me, so they don't let us check it. Even now, when I left for work, she said: "Are you going to leave for work?" I said I would, so I asked the nurse when the doctor would see us. She contacted the doctor, and the doctor said she would see me during the week, but she also said she would see us before Carnival. Now, sometimes when I go out to get my insulin, the doctor is in her room, just messing around on the computer, doing this and that, but she doesn't see any of us, any of our patients here. Then one day I caught her off guard, so I went there, and she was there, and I said, "Thank goodness I was here, now I'll talk to you." Then she was there with the girl who 's an intern, because I saw they work with them. She said, "But she's only seeing urgent cases, not us." I asked when the doctor would see us, when the doctor was in the house, and the only thing she'd tell us was, "She'll be here next week." But will she see us? Because last week you told me she didn't see us. She said: I don't know, but your name is there. That's all she can tell us. I don't have any information. I've had situations where I started losing my vision, everything was blurry. I told her, she said she'd refer me to an ophthalmologist, and so far nothing. She said she'd refer me to an endocrinologist, and so far nothing. To start using insulin, I had an exam last year, in August, when she prescribed the insulin. When the results came back, she remembered I existed and said to me, "Look, here are your exam results." When I saw her in August, she said to me, "Wow, that's when you had that exam, and they didn't do anything." Normally, for someone who knows, a diabetic like me at 24 years old, it's 90 to 100-110 at most, but my blood sugar was at 540. So, I don't understand.

**Discourse:** We're obligated. We can go anytime, both me, her, and others who have diabetes, but it's less likely we'll lie down and never wake up again, because the other day I went to check my blood sugar, and I thought it was very high, because my blood sugar reached 700, and then I measured it, and it was 57, and I use 47 units, which means, for those who don't understand, it's a syringe of insulin, and if I used that syringe of insulin, I would die in here, I would die in here.

**Discourse:** I'm monitoring her diet, and it's not good because she 's not getting anything to eat, because she can't eat, and she's eating carbohydrates, and it's very bad, which worries me, you know?

**Discourse:** Sometimes when we feel unwell, we call the lady, and she says, "Oh, here's a dipyrone to help you feel a little better." When there's no dipyrone, she turns to us and says, "Go take a cold shower, she won't go there anymore."

**Discourse:** A lump appeared in my breasts, she said, I had the exam done, but I never got the results.

**Discourse:** Because there are three nurses there, you feel humiliated, there's also the doctor, it's very difficult to get the exam done, only by the mercy of God.

**Discourse:** Most of the women in prison are uninformed. Because there aren't any lectures about STDs in prison, I've lived with someone who is HIV-positive, and I've also lived with others who wanted to hide it. That's not shameful; shameful is not taking care of yourself. And unfortunately, women who have tereré (a type of yerba mate drink) don't like to share it, and that's not how it should be. It used to be that way. We need to sit down and listen to the health sector, and sometimes there isn't time for this to be said, to be discussed, and they end up feeling belittled. So it's difficult, but that's a part of health, that sometimes the girls say, "Oh, he doesn't need to interfere," because there are many homosexual relationships here, one person is with another, then they're with another, and it ends up narrowing their minds, they no longer have the perspective to see if, you know, and sometimes that affects their psychology because they keep thinking about that relationship they had. So, I think it's more along those lines, if everyone were guided to understand that it's not like that, to see it that way, that they're not going to get involved because of a spoon, I think it would be easier.

**Discourse:** And here I think there are four nurses who are there every day, and you go in there, first of all, they never give a lecture, they never even explain anything, but if you go in there for any kind of care, someday, they don't even say good morning.

**Discourse:** There used to be a lot of lectures, in 2006 or 2007, but today there aren't any. I even went to the administration to ask for a lecture bout HIV, because in our cell there's a girl who's prejudiced against smoking a cigarette, and people say she'll catch it. I've already experienced prejudice in my dormitory.

**Discourse:** I believe it's because we are the most vulnerable group; we are confined among many people, so the risk of contamination is even reater, right? The spread is even greater, so I believe that's the reason.

**Discourse:** Not here, but I've been following it from the street and ... we can follow it on TV, but some accommodations don't have that.

**Discourse:** No, they hid it from us when... when it was in 2020, when there was a high rate of Covid, they hid it from us, and there were many people, at least the ladies, with Covid inside, and some inmates with Covid, myself included. I spent twenty days here before with Covid without knowing what it was.

**Discourse:** In this world, they're trying to hide from us that we're inside, and it's very difficult. It wouldn't even be possible, so it was a lie that trucks were passing by a body like that. So it was something that was impossible. What was going to happen? Were we going to die? That's what we were thinking. We're not going to die? It's over, the world is ending and we're in here and they're not giving us any information. We thought they were going to lock the door and let us die from the virus here.

**Discourse:** For example, when I got the video after a long time, I was really sick, I had lost my sense of taste and smell, I had a fever, on top of a fever, and I found a lot, a lot, a lot of stuff lying around, and from there it was one thing, then another, when you look at it they started taking us out, saying it's no use, that's what's the point of taking us out after we've already been through the whole cell, then they took us out, threw us way back there, I was really, really, really sick, I couldn't even stand it mentally, it seems like my head was...

**Discourse:** People don't have time to get vaccinated; they ask who
wants to get vaccinated, it's an invitation, they just ask who wants to, then they leave, they open the door and three leave at a time. Once, a girl said, "No, what if it's the chip?"

**Discourse:** Sometimes we speak like this: "Ma'am, please, you... Madam, here in the others... Please, he's feeling unwell. Wait." So, the way he was treated was inhumane.

**Experiences with TB**

- Information received about TB in the Prison
- Fear of contracting TB
- Fear of passing on TB for family members during a visit

This document presents the verbatim statements of the participants, which constitute the database for coding the Experience with TB in PDL. The transcripts below detail the participants' narratives about the quality of treatment, the time to diagnosis, access to medication, the challenges of living with the disease (including fear and prejudice), and the importance of information in managing TB within the prison system.

**Discourse:** I've been working as a health agent for two years and six months , and within the prison gallery, six cases of tuberculosis have already been treated. None of them were within my own cell, but within the gallery itself. Regarding the treatment from the health department, from the infirmary, it has always been adequate. One thing I question, actually I question it, but I can't change it, is the issue of when someone with tuberculosis lives with others who don't have it. That's the point I'm raising. But overall, the prison system's treatment of those with tuberculosis has always been adequate in the gallery where I am.

**Discourse:** Actually, to correct myself, this week a guy came to live with us in the same cell as someone with tuberculosis. He had already been undergoing treatment outside for four months, and he needed to be taken back in. So he's continuing his treatment there. But we need to know what tuberculosis really is to see if it's truly contagious, if it can infect other people or not. So I can't just say that a person has to be isolated if there's no way of transmission. Because they have their own dependencies, their own private lives. So I can't just say they can't live with me because I don't know the means of transmission and how they get infected, or their personal lives, or because we have seven people in the cell and I don't know if it's someone who has tuberculosis. If it's someone who has tuberculosis, if it's someone who has tuberculosis. If it's a hot place, I'll tell you, or if it's a well-ventilated place. So I can't say that because I don't know the level of contamination.

**Discourse:** I've been a health worker here for twelve years, even at the previous penitentiary. And the treatment has always been very adequate, everything. I've lived with people who had it, but they were taken care of. But you can't just isolate everyone. So prevention is done, everything. Everything is always handled correctly. The health care here is excellent. It always has been, you understand? Everyone is well taken care of. The hospitalizations, everything. It's always been handled correctly. And regarding those who come from the outside, because they're already being treated, right? They just continue their treatment here inside. And here, the area of care regarding health, regarding TB, has extreme control, right? No... There's never been a single case of anyone dying from TB here. Everything is well handled, well organized. Everyone receives their medication, the tests, everything is done directly. Everyone who has a health problem, that we notice, is immediately referred to where they can get tested and start treatment. So that there isn't that time for it to accumulate, right? I've been a community health worker here for 12 years. And the treatment here has always been very good. From TB nursing to other situations. The facility is always well-organized, right? And the care is always excellent, right? There's nothing to complain about. Because I've been around for 12 years, haven't I? One, two days, a year, two... But it's been a while. If there was something wrong, we would say so. No, it's excellent. There's nothing to complain about, right? The facility, the care, the nursing, everything. Nobody goes without medication in any area. They are always well cared for, well assisted. Psychologist, psychiatrist, everything, you understand? When it's reported and confirmed that it's TB, the infirmary is called. They talk to the psychologist, everything. And the treatment starts right there, you understand? The treatment is given at the door. The nurse comes, pays for the treatment. Everything is done. There's nothing... It's all within the correct requirements, right? Because it's a penitentiary, right? There's no specific place to leave them, right? Go on, you have to know how to live. You have to isolate the cell there. Those who are in the gallery, isolate that cell. And make sure the others don't get it. Up to 10 days there, and then release them.

**Discourse:** I've had tuberculosis before. I received treatment, but it was in another prison, and the treatment there was exceptional. Just like I see here in this penitentiary. Everyone receives their medication at the right times and it never runs out.

**Discourse:** Fatigue, fever, and loss of appetite. These were the symptoms I experienced.

**Discourse:** A simple test to diagnose the problem: a sputum test.

**Discourse:** I had tuberculosis in 2022, in another prison, but I was treated well there too, treated for six months, and the symptoms are sweating, loss of appetite, you lose a lot of weight.

**Discourse:** Yes, we always and frequently receive lectures about diseases and vaccines, always advised, to prevent them. The lady there has already participated in several meetings together and when it appears, as soon as they see that it's not possible, the sputum test, the infirmary immediately sends for an X-ray, when there isn't time for... Because then it appears immediately, right? The sputum test, the X-ray, then there isn't time. Then they go ahead and take preventative measures right away.

**Discourse:** This year... This year, actually, since I've been here for two years, nothing has been achieved. Last year we did achieve our goals; we distributed pamphlets and even put them up, dividing them into two galleries, A and B, right? We put them on the wall so people could see the level of contamination, all the things, the duties, the precautions, right? They achieved their goals, they have the pamphlets, we put them up, right? But this year, unfortunately, that wasn't done, right? Therefore, even our meetings with the health agents, this year is the first. If the guy there has been here for 12 years, correct him, it's the first, right?

**Discourse:** Correction. There was one in January. There was one in January.

**Discourse:** I've been here for a year and nine months, but I haven't been called.

**Discourse:** I've been here for a year and two months, I wasn't invited to this meeting, was I? Suddenly, everyone is speaking for their own group.

**Discourse:** One year and nine months, a little over a year here, and I haven't been called yet.

**Discourse:**  Well , for about a year and two months, more or less three months, I still hadn't been called. There was no information about Tuberculosis, so... This year... There was no information. Last year, neither.

**Discourse:** The only problem, which I don't know if it's suddenly... It's not the prison's fault either, I think it's just that... It must be from outside, something that he... Sometimes there are prisoners who take three, four months to find out... I don't know if it's from the disease or not, right? They find out, they go there and take the test, and they can't find out if it's tuberculosis. There was one there who... In the gallery where I am, who... He, I think on the fourth, third or fourth time he took the test, he found out he had tuberculosis. But, you know, I don't know if it's suddenly from the disease, sometimes it takes longer to manifest, or something like that, but about the prison, the prison provides the right treatments, the tests are done when requested. That can't be denied.

**Discourse:** In our cell block, we had a guy who didn't want to take his medication because of the side effects it was causing. In that case, the guard said he had to be isolated, he had to leave the cell block, he had to be isolated. Then he decided to take it, otherwise he would be isolated.

**Discourse:** Regarding tuberculosis, from the beginning, when he starts sweating, complains of back pain and fever, we refer him for testing. It takes a few days for the results to come. How many days? Tuberculosis is still transmissible. Transmissible.

**Discourse:** Most of the time, they come to us when they're already in that situation. They come, and we tell them, "Look, I'm sweating, I have back pain, I have a dry cough." And then we give them their name, and as I said, after 10 days, they're often lucky and they come in the week of the blood draw, but when it takes about 15 days, they usually complain that they're coughing up blood. It's very common; when they cough, it comes out, but often it can be from their throat because it's dry, and the cough irritates it, and I often monitor that too. I check the color of the blood, the frequency, whether it comes out continuously or just a little, only in the morning; I'm always monitoring that.

**Discourse:** This gentleman already had a test once and it didn't show up as Tuberculosis. So now I had to walk again, and this week I walked again, but I insisted on... Suddenly, I don't know if the disease takes a while to show up, or if it might already be there, but sometimes it takes a while to show up, and it goes untreated, and it doesn't even show up in the tests for 15 or 30 days. No. It will show up on the third, fourth, sixth time the test is done.

**Discourse:** Since 2001, when I was imprisoned at PED, I've been taking care of the sick. Back then, I was already taking care of the sick. In cell 4, I had Guilherme; he weighed, I think, about 30-something kilos because of tuberculosis. I looked at him and thought he was going to die. That was back then. But then, as the years went by, you realize it's not quite like that. Many times, your own body has ways to defend itself. It's natural; you don't need medicine. You can't take chances, you always have to use [protective measures]. I even try to ask the nurse for a couple of masks to get them into their cells. But he knows how it is. He uses them for half an hour, and then forgets about it. And about catching tuberculosis, some of them get it. There's one in the cell who gets what... I don't remember, but the guy had it in cell 8. He left the cell, changed cells, and Eduardo stayed. Eduardo has been taking it for three months now." How much is the treatment again? How long is it? 6? 6, yes, he has two months left. He's already been taking it for 4 months. And he caught it from the guy who wasn't in the cell. But only him. I already noticed that he caught it because he's very thin and doesn't eat properly. He even has the purple pills that I give him every day before meals. Ferrous sulfate. Supplement. And he was the weakest in terms of heat.

**Discourse:**It dries up and comes back. And he has HIV, you have to see. He has HIV. It's also something he's not cooperating with to cure, right?

**Discourse:** I would accept it. I saw in 2014, 2014/2015, a huge man, the size of participant number 6 here, shrink to the same size and be taken out of there, it wasn't in this prison, no. It wasn't in this prison, it was in another one I was in. I got out of the chair throwing everything up and then the guard just came back and said he wasn't coming back, that he was done with tuberculosis. He was the same height as me, he got out, but vomiting, like this, I don't know, but his tongue was bigger than this table here, everything was spilling out, balls of blood, down the corridor, in a matter of 5 to 6 months he was my size until the disease consumed him. Of course, I also think he didn't really push the treatment, you know, he didn't push it, he kind of abandoned it, thinking he'd get better anyway, and he never came back. That was in 2014, so I'm a guy who already has this disease, a disease that scares me. Not for myself, but like, in a little while, let him catch it and pass it on to someone in my family, he was already coming here, you know? He's already coming here, actually, suffering as he comes to live, and then I could bring something to him too.

**Discourse:** One of his questions was this: if we make a vaccine, if we make a tuberculosis vaccine for everyone to take, even those who don't have it. Just because you saw someone die of tuberculosis doesn't mean you're going to get vaccinated. If I saw someone get into a car accident and they were all mangled, does that mean I'm not going to buy a car? It's complicated.

**Discourse:** Tuberculosis is a topic that's very much on the mind here in the prison. And to pass on the information through meetings, which you pass on here so I can go there and pass it on to them, I'd ask them the same questions, what do you think about the tuberculosis vaccine now, who has it and who doesn't? It's for everyone to prevent and also to cure it. What I learn, or study, or participate in outside the prison, I pass on to everyone there, cell by cell.

**Discourse:**  Yes. Sometimes, as I'm saying, there's a problem where it doesn't come through; one time comes, then another time comes, and our bodies don't balance things out properly, right?

**Discourse:** There's a lot, yeah, because, you know, we're isolated, like the brother said, then the 15 days pass, which is supposed to be the contamination period, right, as they say. Then, during those 15 days that we're there, we're at the mercy of the situation, like he said, because we're not being assisted. Like, I was there too, I spent 24 days there, when it was supposed to be 15. Then it took a while for the other container to arrive to do the test, so we're in a cell, alone, isolated, next to the infirmary, which is also the infirmary, but we don't get the proper treatment. They isolate us from our cell, we're getting more care from the others, they put us near them so we can be cared for by them, and we don't really get that care, you know? We're at the mercy of the situation.

**Discourse:**  When I broke my leg in prison, I was put together with those who had tuberculosis, you understand? Because the prison system doesn't differentiate if you have a broken arm, a broken leg, if you have AIDS, if you have tuberculosis, it doesn't differentiate. They throw you all together because they can't meet everyone's needs. There, you understand? The system can't accommodate everyone, each one with the correct treatment, you understand? So, I spent seven months there treating my broken leg. I didn't have tuberculosis at all, but I was living with people who had tuberculosis right next to me. When I went out, I came back, I caught tuberculosis, on the street, on the street. When I got back inside the prison system, I notified the staff. I'm undergoing tuberculosis treatment, can you send me medication? They didn't pay attention. When I arrived at this unit here, two months had already passed, they went to the SUS (Brazilian public health system), they saw that I had already been marked as having given up. So I started the treatment again from the beginning. So, I spent five months out there, and I've already started the six months again. For what? I spent eleven months taking this medication. And for a brother who has tuberculosis today, who doesn't know he has tuberculosis today, he'll only find out if he gets a fever, if he really needs it, even if you see our whole appearance changes. We get pale, we get a high fever, there's a tremor in the body, loss of appetite. And we don't have daily access to testing here. We don't have that. We ask and the nurse will see if you really need it, but she doesn't know what we're feeling at that moment. Then yes, there will be testing, but not for all the cells, it's only for one person. If one person in the cell has tuberculosis, which was my case, everyone will have to take the test. Then yes, you understand? But the others who have it and don't know, whose condition remains incubated, won't take this test, you understand? So, I think the prison system needs to improve its technical staff, I mean, with more nurses, with more assistance from technicians to be able to take care of each one of us.

**Discourse:** In my case, just the little container, the sputum test. When the result came back, the medication came for me and the little container for everyone in my cell, you know? Then everyone already looks at you differently. Why did the little container come for me too? Oh, you have TB, right? I said, I have treatment outside, I'm starting treatment again here. But is yours transmissible or not? I said, I don't know how to explain. It's the first time I've had TB, you know? We also didn't have a lecture for us to know how to explain to others what TB is, you know?

**Discourse:** Here in the unit, like our brother said, he doesn't have, how do you say, he has no way of knowing, right? But, like, it's only from the moment we have it. We know, sometimes we're not educated, but we know that when we have something that we're feeling, that we... That's when we'll ask for medical attention, you know? So, the demand is very high. Many of us are almost more than eight hundred. You know? So, to meet the needs of this unit here, there isn't enough, you know? Like, as the brother said, look, there's a symptom, I'll go, and if I have tuberculosis, I'll isolate myself, and it's good to do the same with the rest of the cell. So, he doesn't have enough. It's the first time I've seen this here in the unit. This is even good for us, a study like this, to keep us informed more and more each day, you know? Regarding... this disease, you know? And also so that we can take information inside, because here, through us, I believe today that we will be intermediaries for our brother inside. So, like, they should be more rigorous about that, you know? Like, because we have demands to go from cell to cell to monitor other things. They should also do that regarding TB, everything they do. Go from cell to cell to properly monitor the inmate's life, you know? Something that doesn't happen.

**Discourse:** He was infected with TB, and he had an allergic reaction to the medication that was initially given to him. He had an allergic reaction, then another medication was given, and he had another allergic reaction as well. And... I think it's quite negligent on the part of the system, the administration of the system, and also the health administration of the unit, because... Even knowing that the problem is a transmissible infection, the young man remains with us, in the cell, inside the classroom, even though we are infected, even the teachers, right? And we asked the director, and several people, to look into his situation, and they continued, he's taking medication, like, for what? Medication for allergies, he's taking it for the same purpose, for his health, to protect his cellmates and the people in the classroom. So, it's negligence, and it needs to be addressed in all units, not just this one.

**Discourse:** No. This is the first time. This is the first time. No. I've never received any information about tuberculosis. No.

**Discourse:** So, when I found out I had tuberculosis, I had to say I had it. I went to the infirmary because they had already told me I had tuberculosis. I went up to the infirmary and told them I had tuberculosis. The nurse said to me: "How do you know you have tuberculosis? Because of the symptoms, I can't eat and I'm getting weak, I have a dry cough and so on." I explained the symptoms, and she said it didn't mean anything. Then I asked if I could get tested, and the nurse told me they weren't doing the test and that it wasn't done that way, she had to schedule it. She couldn't schedule it after a month, but after a month, she scheduled it, but I was already coughing up blood, and even then she didn't believe me. She sent me back to my cell, and I waited fifteen days for the test results to arrive. When they arrived, I really did have it. That's how they found out.

**Discourse:** I already have, I already have, I didn't.

**Discourse:** The lady was saying that there would supposedly be a new vaccine for TB, correct? Well, I've already had TB, and it's quite complex, and even if you're undergoing treatment correctly, you can still get it. Many people undergo treatment to get cured, and then your breathing is no longer the same; you can't even run without getting tired. It affects the lungs and leaves lasting effects; very few people are left without lasting effects from TB. Regarding this vaccine, what would it do? If you're in the know, how would this vaccine help fight the virus, combat the virus, or improve the person after they've had it, with that developmental stage? Would it manage to kill the virus? Because many times, even after treatment, more of the virus remains, and when it returns, it comes back worse.

**Discourse:** I'd like to know about the new vaccine. Since I've already had it, my lungs are affected, so it's like a vaccine that would expel the virus, because with COVID, a vaccine was created to fight the virus, like if you haven't had COVID, you'll receive the virus and fight it head-on, like with TB. I don't know how this vaccine works. But it would help, but in what way? In its development, the person doesn't suffer the after-effects, right? So how does this vaccine help? How would it help with the suffering of someone who has already had it or who might get it? How would its development help? Thank you.

**Discourse:** I will continue to accept this invitation because so many things happen in the world and we are the last to know, we can no longer know, for example, even about the vaccine, we are practically the last to be vaccinated, for example, it's an opportunity like this, why not try? Because I caught COVID and didn't know, I was infected with the virus, I only found out when I got tuberculosis, when I caught tuberculosis and I found out that I had caught that damn thing, but I didn't feel any effects, I didn't feel anything, I didn't feel anything, I didn't feel any effects, and when I went to get tested for tuberculosis, to measure some other tests, to see if I had HIV or another type of disease, I had a test there, which I also did for tuberculosis, so why not participate, right?

**Discourse:** I had the experience that a friend in my cell had tuberculosis, he left, stayed for 15 days and came back again. I even saw the medication he was taking. But then I saw him vomiting blood again, and I had to call the police. Unfortunately, they say tuberculosis is contagious, it kills, you can't use the same spoon, the same cup, it's impossible to live with someone who has tuberculosis next to you. I saw him vomiting blood, saw him taking these huge pills, four pills a day, he smoked like crazy, he was going to die. While smoking, I think the disease came back because he was vomiting blood, I'm telling you something I saw, I saw it.

**Discourse:**I broke my leg, spent two months in the infirmary, and saw more than five people die from tuberculosis. More than five died.

**Discourse:** I have it. I think I even caught it. I'm dying little by little, I've been here for three years, I'm losing weight, I've lost about 20 kilos.

**Discourse:** I've been here for 3 years.

**Discourse:** No, I did it in the middle of last year. Last year? I did it and nothing happe.

**Discourse:** In my family, I had experience with my aunt who had tuberculosis, underwent treatment, and she smoked a lot. She quit smoking, and she was cured of tuberculosis. I don't know if tuberculosis is curable, though. However, she had a worsening problem in her lungs, caused by the tuberculosis and smoking. Nowadays, she uses an oxygen tank; she's like, let's say, a compressor—she can't live without air. She carries the tank around all the time because of that. And as I said, 20 years in the prison system, so I've seen many cases of people dying, people who are being treated and undergoing treatment. In the last prison unit, I lived with prisoners in the same cell. I didn't get tested, but they were in treatment, and they said it was improving. So much so that they bring prisoners from other units here to this infirmary. They say it's very good in relation to tuberculosis. All the prisoners from the units I've been to are treated here. They're brought here. They say that regarding tuberculosis, they give a lot of attention and treat them very well. They really treat them, they give a lot of attention to the prisoners with tuberculosis, they give medication, and until the prisoner is cured, he doesn't leave here; he stays hospitalized here. In that regard, it's very good here, you understand?

**Discourse:** In 2014 I was released on parole, and so, to avoid using my real name again, every time I came back, I gave the wrong name. Why? To get away with it. Then I fell into the system, and they said I had tuberculosis. Why? In César Gameleira's cell, there was a shack, they said it was a storage room, the guards wouldn't go there, and I had to stay hidden to get my pay. I wanted to know why I was in the tuberculosis cell six times, I gave the wrong name, I never had it, I was in the tuberculosis cell, I didn't get it, why? Six times, I wanted to know why I didn't get it, but I didn't get it, I didn't get it, I didn't get it, I didn't get it, I didn't get it.

**Discourse:** I've been to all the units, I've attended several lectures, they give out this booklet, the damn yellow cup, that's the one.

**Discourse:** It's him, he has some drawings on him, with the symptoms written on them: fever, sweat, cough.

**Discourse:** I don't know anyone in this unit, I'm 32 years old and I've been here for 6 years.

**Discourse:** In another prison I was in, two people died, not in the same cell, but in another one.

**Discourse:** Last year I was in the "Luta Ladeira" prison, there was a huge tuberculosis outbreak there, that's where I lived in that cell with prisoners, and then I was transferred to another wing, I arrived in another wing, there was already another prisoner with tuberculosis, he was also going to the infirmary a lot. I didn't witness any.

**Discourse:** I have doubts about whether there is a cure or not. In my aunt's case, she had all the complications, so it's good that you're here to clarify the doubt, because I myself have my doubts about whether there really is a cure.

**Discourse:** I also have doubts. Because I had a friend who lost his father to Covid; he was undergoing treatment, but he still passed away. I think it also depends on immunity.

**Discourse:** In our cell there was a young man who had tuberculosis. He was undergoing treatment. He was taking many, many medications. He followed the treatment correctly. He managed to finish the treatment. But he became very weak. He even became very thin. But regarding the tuberculosis treatment, they don't leave anything to be desired. The facility is efficient in treating TB. However, it's complicated. Because you're living together, the environment is closed. There's little ventilation. There's nowhere to run. So everyone is susceptible to catching the virus. To catching this disease. Regarding the disease, because of the large number of people there, there's a lack of management in separating people who are already identified with tuberculosis. They should be separated and placed in a specific cell so that those who have the disease don't transmit it to others.

**Discourse:** It's almost exactly the words he's saying. I've lived here in prison with several people who have confirmed tuberculosis. Thank God, I've never had it. I've always had the tests done here. Even regarding this matter. I don't see much complaining because we're always getting tested. We're always having sputum and blood tests done; they're always doing the tests. But it's just like he said. We're always living with people who have the virus and are susceptible to the disease.

**Discourse:** We've already received information. There are conflicts. We received them some time ago. And in all the time I've been here, every year, the girls come to have their tuberculosis tests done. And they are always very well taken care of. There are no complaints, they are well prepared in that regard. Absolutely.

**Discourse:** I caught tuberculosis four years ago. But the example was even before it faded. Then the medicine came, it arrived correctly, and it came to the prison. You know this group too, it came here. I stopped my medication and it ran out. I was taking it, I recovered very quickly. Because I almost died. Mediator. But did you have information about the disease? I received everything correctly.

**Discourse:** I was there before, in 2019. And in the cell I was in, there was a person with tuberculosis. And the follow-up and treatment were very difficult. There was a moment when the cell leader had to put pressure on them because that person refused treatment. And they were very thin, and it was a difficult situation. But another factor is overcrowding; it makes treatment in general very difficult. Very difficult, you know? There are so many people, so many elderly people, it's very cramped, everyone is right next to each other. We are really susceptible to catching it, to being infected by it at any moment. And the treatment itself will always leave something to be desired because it can't meet the demand. At the exact moment you need it, even though the system tries to meet the demand, it really falls far short of expectations.

**Discourse:** No, I didn't have that. Now, as he very well explained, there is a very specific job for this issue. That can't be denied. To care for, to attend to. There are patients who end up making it difficult, but they have an intention to take good care of them. But in general, other things, as needed. But there is indeed a very great concern in combating tuberculosis.

**Discourse:** She had tuberculosis, she isolated herself, then she came back
 to live with us again. live with us again , but I don't know if she got better or not.

**Discourse:** I had two experiences. One was with Yara, who came here very reluctantly. But she underwent isolated treatment. She felt that there wasn't a proper treatment. Later, she spoke with us and got better. The other was Dona Varena in Mar, at the PFM. She had normal experiences. She didn't know she had tuberculosis. She had a dry cough. She left very weak, because she only gained 6 kilos a year. She arrived there and passed away. When they discovered it, it was already very advanced.

**Discourse:** She only found out she had tuberculosis when she was outside. And she already had the virus, tuberculosis, for some time.

**Discourse:** Because they say that tuberculosis kind of goes dormant, right? And that's why it manifests itself in a person. Especially when we're around people who have it and don't know it. But they did everything right.

**Discourse:** Yes. My mother, she separated from us. Because I was little, she separated from us. Because at that time, you know? My brother caught a lump here on his neck. Then he had surgery. So, well, at that time he didn't have it. And my sister, Cassiola, she caught it, we didn't have it... But not everyone did. We all had sputum tests. There were 6 children. We didn't have to do it for everyone. But she did the treatment properly. And they say that when you start treatment, you don't transmit it anymore. And she had the test.

**Discourse:** My mother smoked. And the others did too. They both smoked, but the seed didn't. So, it's impossible to know if it was just the tobacco, right? It could also e hereditary, I don't know.

**Discourse:**I don't have that kind of prejudice. Because I interact with people like that. But, give me patience. I don't know. Even someone taking medication, saying it doesn't go away, people who aren't experts say no, that it does go away.

**Discourse:** When she arrived at the unit, I think she already knew what she had, but I think she was afraid to speak up because of the prejudice. She went straight to the ward, to a cell, lived with the people, and two people were suspected of having tuberculosis. That's the process she was going through. They were tested, and all of them were detected, except for the other two. When she was in the cell where she was living, two people were infected. When they found out, it was too late. They were isolated. She should have seen the results.

**Discourse:** Everyone in the cell tested positive, and two people did. I think because she thought there would be prejudice, she didn't inform anyone in the unit or the other people. Everyone was probably invited to get tested.

**Discourse:** Right now, only the people from the cell were tested. After two or three weeks, the entire ward had to have their sputum tested. Nothing else was detected. But especially those two, those three, were isolated and started taking their medication properly. Right at the beginning, you know? There's treatment; the people were isolated so as not to infect the entire ward.

**Discourse:**At that time they were informed about TB.

**Discourse:** All of us, from the pavilion to wing B. So we went to get tested, 15 days after this happened.

**Discourse:** Yes, information about TB.

**Discourse:** I never had any information. I never had any information about this disease. I don't know it, I don't even know if it's bacteria or a virus. Normally, I don't have any information like that.

**Discourse:** Like one, a young woman who came, who left isolation, she was being treated for TB. She's still here. And then she was isolated, all that isolation stuff. Then she went to the ward, to live with us. And then she started treatment again. Then she started, you know? That she had TB, and she was being treated, all that. And she explained to the girls that they shouldn't drink from her cup, they shouldn't use her spoon. How they should treat themselves in case they didn't catch the virus.

**Discourse:**We don't really know how it's contracted, I don't even know what kind of lump it is anymore. More than just one kind of lump. I didn't know that, I didn't know it gave birth.

**Discourse:** Fever, failure, cough, shortness of breath. Dry cough.

**Discourse:** Recently I had a problem with my breast, and the gynecologist said it could be mammary tuberculosis, not mastitis. I didn't know that existed. Does mammary tuberculosis even exist? She said she has to come back again now. And I'm left wondering about that.

**Discourse:** I lived with someone, whose name I'll mention here because she's like a mother to me, it's Dona Leila Maria. She went to the hospital, São Julião Hospital? São Julião, she stayed there for more than 40 days. She arrived very thin, she was a drug addict, she contracted tuberculosis on the street due to lack of treatment, and then she was supposedly hospitalized. When I arrived here at the prison, on February 3rd, she was hospitalized, and she was discharged at the end of February. So, I met her, she was just starting treatment, the treatment is for 9 months, right? At São Julião, I don't know if that's the standard, but that's what was said. So, it was very sad to see her suffering, you know? The bloody sputum, the thinness, her lungs, the daily shortness of breath, this situation itself, a deplorable physical condition. And when she left here, she was already recovered, she had been discharged, Dr. Maurício discharged her, and she was beautiful, she was already heavier, you know? So, this gave me a different perspective than I had before because I used to think, "Oh, I'm sitting here next to Clarice, I'm going to catch tuberculosis." No, it's not like that. Of course, you can catch it through saliva, right? Something like that, it becomes viral, right? Viral. So, I had a different experience because we talked all the time, we were close, close all the time, we hugged, I took care of my things, she shared, we shared the cup, we shared the spoon, you know? So, I paid attention to the fact that tuberculosis, of course, is a very dangerous disease, very dangerous, right? It has to be treated, treated very riskily, right? But, that we can live with the other person without fear, without fear of being treated as a human being.

**Discourse:** Let me tell you something. I had tuberculosis, but I got sick because I had asthmatic bronchitis, and I saw them saying it's nothing, they said it's nothing, you have one, but no, it's nothing. And I'm still feeling sick, feeling sick, feeling sick. Mrs. Léa, who is a psychologist, she helped me, she went there three times, and on Saturday she couldn't speak anymore. So I was just like that, I couldn't speak, and Mrs. Léa called, we arrived at the nurses' room, right? Mrs. Michel and Mrs. Priscila, they even called me upstairs, I don't have to say anything, I heard that, because she's very ill there, at that moment Mrs. Michel said I had to take the test, so I did and it was positive, but I was in such an advanced state, I was so sick, to get out of bed the girl had to help me, sit up, take off my shirt, I couldn't, because my bones hurt so much, at night I slept with five blankets, my head was going to burn from the high fever. Then, I saw Dr. Maurício, because he only treats this disease, so I went to do the treatment they told me about, but I know they have treatment, but I also got very thin, at the beginning of the treatment it's bad because you want to stop, it makes you very nauseous, you feel very bad, but I managed to do the treatment correctly, but it left a lot of after-effects, because my bones, my shoulder, you know, hurts a lot, like the vitamin, the doctor, here for you to see the doctor, and then you wait to die.

**Discourse:** No, I got there and went in, the nurse said, "I'm going to tell you something , you have tuberculosis." And then, like, I started crying, it felt like I wasn't standing on the ground, and she said, "Oh, but you didn't expect this?" I said, "No, I've never had it before, who expects illness? Who expects illness in their life?" I told her, and she said, "Oh, now you have to do the treatment." Oh, Dr. Maurício, who is a very good doctor, thank God, if it weren't for him, I think I would have already died in this prison, because I think I would never have found out, and if Dona Léa hadn't written an official letter helping me find out, I would have died. And the people in my cell, I mean, can you tell me, there wasn't anyone on my side, because everyone was afraid of catching tuberculosis, because they thought I was some kind of monster, a terrible disease, so at that moment I felt like only God and a few other people were by my side, the rest didn't even come near, because it seemed like I had a disease that was going to kill me. So, it's very painful, I cried.

**Discourse:** I lived with four girls who had tuberculosis. The first one who had tuberculosis, we called the security team on her, and people said it was just a simple flu, but the girl went to the health center and they found out she had tuberculosis. Another one here, a colleague of ours, she was on medication for two weeks. When the medication starts, it's every day before meals, usually before or after, and you'd see the girl would take it at 10 or 11 in the morning. She had already eaten bread, she had already eaten everything. And also, two weeks without taking medication, all four girls I lived with had this problem, of stopping the medication.

**Discourse:** I also lived with Dona Leda, and the prejudice was very evident because people stayed away from her, away from her bed. My bed was above hers, and I was one of the few people who talked to her in the unit. She was an elderly lady, and she wore a mask all the time. And the prejudice was even worse; people didn't even want to go near the back of the unit because her bed was there. They couldn't even go there to wash clothes for fear of catching something. And it wasn't just tuberculosis, but various infectious diseases.

**New TB vaccines**

- Doubts about the creation of the vaccine
- Insecurity about the introduction of protocol changes
- Increased knowledge and reliable sources of information
- Fear of not receiving post-vaccination care
- Potential side effects of the new vaccine
- Satisfaction in fighting TB

This document presents the participants' literal quotes, which constitute the database for the analysis of perceptions regarding New TB Vaccines. The following transcripts detail Doubts about the creation of the vaccine and Insecurity about the introduction of protocol changes in the prison system. Furthermore, the statements reflect the need for Increased knowledge and reliable sources of information, the Fear of not receiving post-vaccination care, the concern about Potential side effects of the new vaccine, and the Satisfaction in contributing to the fight against TB.

**Discourse:** Ah, if it were tested on tuberculosis patients in the terminal stage and it worked, then yes. But a vaccine, I'll give you this vaccine, you won't get tuberculosis, but I wouldn't take it.

**Discourse:** We needed to see the test results, right? And you, who produce the vaccine, have to show them, right? Because the important thing is to know the results from abroad.

**Discourse:** There has to be approval, right? With approval, everything works, right? We're going to arrive and say, let's vaccinate all the galleries there, right? It worked in 10, you understand?

**Discourse:** The thing is, most of the time, when it comes to vaccines, they call the on-call person, the health agent, and it's the flu vaccine. Whether you get it or not, you sign a responsibility form, you might get it, you might not. When the subject of visits comes up, the prisoner immediately hesitates. So, I'll get it. There's a lot of talk about this, this, and this. It's the flu or COVID, it's just those two topics.

**Discourse:** The question, I had already asked this question regarding whether they would accept new vaccines. In fact, new vaccines are constantly emerging, more diseases are appearing, and we are developing more vaccines. The need for us to know why these vaccines are necessary, how they work. I, and I agree with the other person (participant two), will not accept any vaccine injection knowing that I am at risk.

**Discourse:** The system of who makes, who manufactures, who produces, who researches new diseases that will emerge, but what do they research based on? Not on what already exists. So how can we accept a vaccine for a disease we haven't experienced? Television is showing that it's emerging, it's already emerging, I don't know if it's been tested, I don't know if it's been 100% proven, for example, HIV infection. Then soon they'll want us all to get vaccinated, for HIV, for example, knowing that we have... I have... my partner "Isis," and she has a clean test, nothing wrong, oh, but I'm going to get an HIV vaccine that will immunize me for the rest of my life. The same thing with tuberculosis. If I don't have... I already know what the contact is like, how tuberculosis is contracted, the first few days of the stage when it's strong, we have a certain restriction, no... not as they say, a... we don't have a prejudice, but we have to have our restrictions, especially us health workers.

**Discourse:** We know what can happen, but I'm not going to accept taking a vaccine, having a vaccine administered to me, knowing that I won't run that risk. In my... in my understanding, that's it, I'm responsible for my... for my continued presence, I wouldn't accept it. Now, the Minister of Health... sends two thousand doses to be administered at the Montenegro Penitentiary, and they're going to administer them. But administer them for what reason, if... this isn't happening? Is it just because they've already proven it's effective, they're going to administer it? I wouldn't agree.

**Discourse:** Vaccines are based on studies and research, but if it proves to be an immunizing agent, I would take it. I've already had the disease... I don't know what it's like. I know what it did to my body. So I would take it.

**Discourse:** From my point of view, everyone has their own, right? I would accept the vaccine if we arrived there, with all the explanations, and said, look... Let's take the tuberculosis patients from the gallery, let's give them the vaccine, you'll see they'll become immune, and then I'll come back here to vaccinate you. You'll be immune forever? That's something I would accept, but now to arrive there and say, oh, you'll take the vaccine and you'll become immune? Then I would be strongly against it, but like, if they came there and proved that the vaccine... Oh, let's give the vaccine. It won't... Those with tuberculosis will be cured with the vaccine.

**Discourse:** And then tomorrow you get the same thing. I'd be against it, but like, if you get there and it's confirmed, look... This person has tuberculosis, let's vaccinate him, you'll see he won't... That his tuberculosis will be cured and he won't pass it on to anyone else, nobody will get it. Then I'd agree, right? But it's something that would guarantee you'd be immune, even because of the visit. Because many times you don't know, sometimes it takes two, three months to discover tuberculosis. Then a little while later your child comes along, someone from your family comes along. You'll see out there on the street that you passed it on to your family.

**Discourse:**I would accept it. I saw in 2014, 2014/2015, a huge man, the size of participant number 6 here, shrink to the same size and be taken out of there, it wasn't in this prison, no. It wasn't in this prison, it was in another one I was in. I got out of the chair throwing everything up and then the guard just came back and said he wasn't coming back, that he was done with tuberculosis. He was the same height as me, he got out, but vomiting, like this, I don't know, but his tongue was bigger than this table here, everything was spilling out, balls of blood, down the corridor, in a matter of 5 to 6 months he was my size until the disease consumed him. Of course, I also think he didn't really push the treatment, you know, he didn't push it, he kind of abandoned it, thinking he'd get better anyway, and he never came back. That was in 2014, so I'm a guy who already has this disease, a disease that scares me. Not for myself, but like, in a little while, let him catch it and pass it on to someone in my family, he was already coming here, you know? He's already coming here, actually, suffering as he comes to live, and then I could bring something to him too.

**Discourse:** And if the whole population volunteered to participate, not just prisoners or inmates, maybe I'd accept, right? If it were a general thing, well, if it was only for prisoners, then no, right? Then it would be like using them as guinea pigs. But if it's a general thing, for the population and the prisoners, then I'd accept, maybe I'd accept...

**Discourse:** But like, accept it, if suddenly it were proven, if it were a simple question, if it proved, oh, it's going to work, bam, we don't have anyone who needs one, like, ah, there are a million people, we don't have anyone who needs one to see if it's going to work, I, in my way of thinking, I'd say, oh, you can vaccinate me, it'll work. But for sure, everyone has their own point of view, right? I think that more, like, if it were about vaccinating, I don't even often go there, most people get vaccinated because of the visit. Or if you go there and say, oh, if there are people in the yellow gallery with Covid, if there are in gallery 1, I think two or three cases, something like that, right, if there are, there are no more visits. Then you go there, you can't vaccinate them, you don't even want to know what the vaccine is, what it is, there are no more, you know, the thing, so, that's how it was, right. I don't even want to know what the vaccine is, if it's going to be good, if it's going to be bad, if it's going to affect me like I did. I was on my left side for five days, I lost all my strength, my arm was tingling for five days, I couldn't grasp any of that vaccine. I'd try to grasp it with my left arm, but it was like trying to grasp it and then letting go, until it wouldn't let go. I was there for five days, I had no strength in my left arm. But I went and got it, I didn't know if it was what it really was, they said it was for Covid, that it was a single dose. But I didn't know if it would really work or not, but I went and got it and I knew. Then, what if I.

**Discourse:** But like with the Covid thing, they gave a dose, a new dose. Like you said, the vaccines come from the Ministry of Health, right? There are health posts on the street too, so why aren't people vaccinated on the street, but prisoners are? Because two years ago, when Covid emerged, there was a certain number of vaccines, like our people, our wives, took the three vaccines and then more. And after we took the three, we've already taken about five more doses. And why aren't people vaccinated on the street too? We end up feeling like lab rats, so they're testing the vaccine on us.

**Discourse:** I think the vaccination could start inside the prisons, but what he said was, if he came here, called everyone together, in a way, a consensus, how many tuberculosis patients are there, there are 50 tuberculosis patients, so let's take those 50 tuberculosis patients, if you agree, in a consensus, but not just come here without giving any explanation, just saying, let's vaccinate everyone against tuberculosis, but without giving an explanation like, oh, this will immunize you for six months, a year, a lifetime. An explanation and then test the tuberculosis patients first, to see if it will really work. Then I would agree. Now, to come and vaccinate everyone, just come and say let's vaccinate against tuberculosis, we have to vaccinate, and then inject the vaccine into their arm, turn their backs and leave, then I wouldn't agree to that.

**Discourse:** Tuberculosis is a topic that's very much on the mind here in the prison. And to pass on the information through meetings, which you pass on here so I can go there and pass it on to them, I'd ask them the same questions, what do you think about the tuberculosis vaccine now, who has it and who doesn't? It's for everyone to prevent and also to cure it. What I learn, or study, or participate in outside the prison, I pass on to everyone there, cell by cell.

**Discourse:** So, I wanted to say, if we take this last Covid vaccine, which they said wouldn't be available anymore, right? Covid is here and it's on the street, but the street got it, but the vaccine that we take, that's what everyone in the cell block thinks about, because only prisoners have to take it, and people on the street didn't. It's the same thing with tuberculosis, does tuberculosis only exist in prison? No, it doesn't, it exists on the street too. Oh, so we're going to test the vaccine in the cell prison blocks there. No. So, if it came, and we tested it, and it was proven to be effective, that it will help, everyone would accept it better, you understand? So, let's only test it in the cell blocks, you understand? The vaccine came, but only we got it, at least I didn't hear on TV that everyone is having to get vaccinated again, you understand? And only us. And then many are reluctant to take that vaccine. Oh, why are we going to take it again?

**Discourse:** I think everyone felt comfortable here, because the conversation started off right, asking who wanted to and who didn't, and what it was about, who it was. And it gave us the chance to say, "Do we want to stay or not?" I think that's it. Now, the part I think is right is that everyone listens to whether they want to or not. I think the conversation always starts right when one person asks if the other wants to listen or not. I think that's it. That was the most...

**Discourse:** So, what you said just now... We still need much more information about what's happening regarding health. This isn't about a prison; it's about whoever has an interest in this. It could be the Health Department or the government. Regardless of who's responsible, that responsibility ceases to exist. But for example, we have 12 health workers here. Not all of them have the information to deal with a matter or initiate what they should. We're dealing with people here who have dental problems, right?

**Discourse:** So I believe that information should be much more frequent about everything that happens in the population. About everything that happens in the medical field, about the studies they are doing, about diseases that are out there, that we don't know how to treat, we don't know how to react to, we don't know how to inform a person about. Because just because someone is coughing a lot doesn't mean it's tuberculosis. So we need to have this information increasingly clear so that we can help people who supposedly have it, or have it, or who need treatment, or who won't. And I think about that too. Because we should have much more information.

**Discourse:** We need more information. Sometimes there are people on the street, who have the daily routine of work, and they don't have the means to seek information. You understand? Because that's where it comes from. So sometimes we have more information here than the people on the street. And the more information that comes to them, the more we'll end up with.

**Discourse:** This vaccine would be good because we live in a very confined space here, we don't get much sun, we spend two hours sunbathing and our immunity gets lower, you know? I think a vaccine like this would be good to combat this type of vulnerability. We have this situation here in this place where we don't eat properly, we don't have... We don't get much sun exposure, we spend more time indoors than outdoors.

**Discourse:** I would like to know about this vaccine, if you could bring, as he said, my brother, the package insert, in other words, the concrete information, the level of research you've done, if it was approved, 100% accurate. This vaccine here will be... You will take the vaccine, and you won't need to take any more medicine or vaccines. Or else, you will have to take this vaccine every month because it's a treatment you have to go through, you understand? So, the information is very important to us.

**Discourse:** Many wouldn't want it because it restricts them to injections. But for those who really need it, for those who truly require it, it would be a good option, you understand? Especially since we all know that injections have a faster effect, right? So, if the information about it is kept confidential, if we know the effects it will cause, if we know what we will really feel with it, it would certainly be good for everyone.

**Discourse:** Because I myself am afraid of catching tuberculosis. Because, especially here inside the prison, there's no way to escape the problem, right? Because sometimes, the cellmate who lives in the same cell or wing, on the same side as the shower, the one with the sun... Whether you like it or not, you'll have direct contact with them, and we can end up getting infected. This is a problem even if the unit treats it, even if the SUS (Brazilian public health system) provides the correct medication, but it will leave you a little weaker, it will diminish your health capacity. So, whether you like it or not, in the future it can cause a bigger problem, even if you get treatment, you'll be a little weaker. So it would be very interesting to have a vaccine for tuberculosis. I would take it.

**Discourse:** I'd like to know about the new vaccine. Since I've already had it, my lungs are affected, so it's like a vaccine that would expel the virus, because with COVID, a vaccine was created to fight the virus, like if you haven't had COVID, you'll receive the virus and fight it head-on, like with TB. I don't know how this vaccine works. But it would help, but in what way? In its development, the person doesn't suffer the after-effects, right? So how does this vaccine help? How would it help with the suffering of someone who has already had it or who might get it? How would its development help? Thank you.

**Discourse:** I'd like to know how this vaccine was developed? Generally, vaccines are developed to increase the body's immunity to a specific virus or bacteria, right? Was this one developed in the same way? Or does it seem to be developed in the same way?

**Discourse:** Because, as I've already said before, I felt harmed by this whole thing, so a question I want to ask is, if it's a single dose, will I have to keep taking single doses frequently? I want to know because it says it's a single dose and I shouldn't have to take another one. And I also want to know what the symptoms are because I've already had some vaccines and I don't want to feel weak or unwell.

**Discourse:**Yes, I would agree to participate in this research, especially because vaccines are very important for our human society. We know that since the last century, many diseases have had their mortality prevented precisely because of vaccines, which provide us with defenses. I believe that with new research, especially on TB, which is very important in our society regarding our freedom and our social interaction, it would be very important and essential. We know that there couldn't be just one vaccine, but rather other types of vaccines because, as time goes by, we know that all viruses and bacteria are constantly mutating and changing. I am sure that there would be not just one vaccine, but constantly changes within vaccines, and with this research bringing adequate information for us to understand what is happening, I believe that we would agree to take it, and it is essential for us humans to avoid both mortality and the spread of viruses to the maximum extent possible.

**Discourse:** I think not everyone would, because you have to see, like the brother here said, several people had side effects from getting vaccinated, I myself was one of them, I got a vaccine a while ago and got sick, not everyone would accept it, but most would, I think not everyone.

**Discourse:** Because I want to know if there's a new vaccine…

**Discourse:** Yes, I would like to participate in the research study. And if, for example, we had more people here, let's say I'm going to participate, right? And then, if we have more than us, will they call us there?

**Discourse:** Yes, I would like to, I would accept being part of research like this because I know it wouldn't just benefit me, but also the multitude of other people who need treatment. So I would be happy if I did it willingly, not just for myself, but for everyone in general. So yes, I would accept it.

**Discourse:** Would it be completely effective? Since it's a serious illness.

**Discourse:** Would there be any contraindications?

**Discourse:** I really wanted to take it, regardless of the information.

**Discourse:** Will it work this time? Will it work this time? It will be effective, efficient.

**Discourse:** I think so, because the rates I see in research, in newspapers, on television, people saying that the rate of tuberculosis within the prison system is very high, higher than outside. So I think so, but I also think it would be the last place I would get the vaccine. I would wait for a lot of prisoners to die first, then conclude that it was tuberculosis, let's take the vaccine there, just like with COVID, first they waited for a lot to die. Then they sold it at a very high price, then it reached the poorest populations, then it became free, but first they stabbed them in the back, then many people died, as the man there says, it was just a little flu.

**Discourse:** I believe.

**Discourse:** Would you accept to participate?

**Discourse:** I would accept, just to see how far it goes.

**Discourse:** Will this vaccine be effective, will it work, or is it just hype? I was going to ask that, if it's not really going to work or if it's just for me to get vaccinated?

**Discourse:** I wanted to know if it was made to cure or to treat.

**Discourse:** I wanted to ask the following: would she do the same as she did with the Covid vaccine? Some people got sick after the vaccine, others had no problems, nothing happened to me, many almost died because of the vaccine, the effects will be the same.

**Discourse:** It would be great if a vaccine to fight tuberculosis became available; it would help many people. The main symptoms, how the disease starts and ends.

**Discourse:** It would depend on whether it had already been tested previously. If it already had a target audience, if it had already been pre-tested, what was the response to that vaccine? If there were any reactions, if there were any setbacks, let's say, in relation to that vaccine. No problem, no problem testing.

**Discourse:** If the vaccination has already been tested on the population, a medicine to have here, treatment with people who come from outside, who can have these tests and more, that's great. But regarding medicines, as you say here, not only here but also abroad, it's a public calamity. Nowadays, the city hall, in the past, the city hall used to provide these medicines. Today we have difficulty buying them, we even have difficulty... It's like, how do you say, you have to scramble, you have to buy the medicine. Because in the past it was donated by the city hall, today you have to buy it.

**Discourse:** A question. Regarding the vaccine you're talking about, is it a vaccine that's meant to be used for cure, for mitigation, or like the others that are only taken for prevention? What would this vaccine be? What would it be? Mediator: This vaccine would be a possible preventative vaccine to avoid contracting TB in adulthood. It would be to immunize. It's a preventative measure. It's to immunize beforehand, or to treat after the person already has it. Because sometimes, there are many vaccines that are released. The person takes them beforehand. But the vaccine is always for prevention. But I can't say for sure, because there are many people who already have it, many are coughing up blood . Mediator : This research group I work with here does the diagnosis, the screening of people, collects sputum, makes the diagnosis, does the examination, does the medical examination, does the X-ray, and starts the treatment. So there is also a treatment. Treatment lasts for 4 months, or however long is necessary for the person to recover. Number 14 also had an experience with this research group in Dourados. That's how you described it. That's how it's done here too. Nowadays, the tuberculosis vaccine, as you've probably already been told, is given when we're children. But today, research is advancing. And this is research that many research groups are doing around the world for adults, to prevent adults from contracting tuberculosis. So, we're discussing this from that perspective, if your question were posed to you.

**Discourse:** I would take it, especially since I've never caught TB, thank God, I've never had TB.

**Discourse:** I think about it myself, because I'm thinking about my health. About being around people who have the disease, or they don't, you know, or they get sick again. There's a boy who keeps coming back, this disease of his comes and goes. I don't know why. It's always coming and going. If he's here, he's already well again. That's what it offers you, that makes a difference.

**Discourse:** Even those who have already had tuberculosis can get it again, right? That's the vaccine. It's another way. A mediator. Would you take the vaccine? And who would give you the most security? Who would make you feel more confident? The health team here at the prison, your family, us, the researchers. Who would you feel most secure with to take a new vaccine? Number ??. You, the researchers. You will be testing it.

**Discourse:** Yes, I think mainly because of the research, right? Because of the evaluation, right? This vaccine, because of the process it went through, the approval by the Ministry of Health, so that its application could be authorized, and that's what gives me security. After the research, the reactions, the types of reactions. Because I heard you saying that when we are born, we receive a dose, right? A vaccine. It wouldn't be this one, it would be a new one. Is it a new one? A mediator. This vaccine doesn't exist yet. We are studying this vaccine. After research, right? After tests, right? It brings security.

**Discourse:** There will be research, this research will arrive here, and it will go through several implementation processes to be approved. It arrives inside the prison, it has already been approved outside, and then it will also enter the prison. And if it's coming inside the prison, it will be approved outside, right? Everyone has used and approved it. This is the prison entrance, right? There are people here who have already contracted tuberculosis, who have been through the police stations and it wasn't easy to cure them. But, despite the treatment, they are doing well, they are well cured.

**Discourse:** I see that any initiative to help the inmate, who is very needy, is welcome. Another research project, like the one you are undertaking, with this initiative, in my view, will give us much more security and peace of mind to accept it. But in my case, whatever comes, I accept. I can't dismiss it. I can't think that this won't affect me, because it will. And regarding the general point he made, I say this: the prisoner, the inmate, is the person who knows best how to fend for themselves. Because I'm learning something tremendous in prison. Because first aid comes from within us. I am a giver of life; I bring first aid. We have a function, a church, and then there's the title of pastor; we're the first to come to help. And there's this assistance within the cell, as we said, it comes from outside, and we have it, so this first aid comes from there. But there are situations that can't be resolved. It needs more in-depth care, more attention.

**Discourse:** Mainly about TB. If the vaccine arrives here, it will be approved, right? If the new vaccine won't cause a reaction in those who already have the disease, if there won't be a reaction. In my case, I've never had it. But if it won't affect those who have it, well...

**Discourse:**That would be very good. In my view... It would be very good. Because we would have a vaccine for Dengue fever, COVID, it would be a good thing... This vaccine would be good too.

**Discourse:** Definitely yes. I think everyone, everyone in general, wants more clarification, everyone wants a vaccine, everyone wants to go home alive, preferably healthy. Here, everything's calm, nobody wants to get sick. So, me and my brother, I've never met him, but he's my brother, if he needs help, I'll help him. We have to help each other here. If a vaccine comes, with clarification, with everything in order, everyone will embrace the cause. Everyone wants to go home. Nobody wants to infect anyone. We don't want to be a disappointment.

**Discourse:** For sure.

**Discourse:**. Yes.

**Discourse:** Yes, definitely.

**Discourse:** Yes. That's what we're talking about. We want, one thing only, we understand that we're going to participate in the research and we could be guinea pigs. But we have to be conscious. We want to participate consciously. Mediator. What does "guinea pig" mean to you? What can you do? We can take the vaccine, if it's in our bodies, we'll be guinea pigs. Until it's approved. And it's all unsafe. Until then, we'll be guinea pigs, using vaccines. Many times there's no certificate of completion. And that could be it. It hasn't gone through several stages. We want that, clarification.

**Discourse:** Yes.

**Discourse:**  For sure. We might have a little resistance though.

**Discourse:** Could anyone resist?

**Discourse:** But the vast majority would accept it; in all prisons, everyone has to go home healthy.

**Discourse:** Ah, but then it's already superior force, right?

**Discourse:** It wouldn't be coercion. It wouldn't be coercion from the moment it was an invitation. So it wouldn't be coercion. For example, you agree to participate in the study, and so on. That's it. It doesn't obligate you, right? It doesn't obligate you.

**Discourse:** Wouldn't that be an obligation? No.

**Discourse:** Including those with Covid, several people went unvaccinated. Many people here are not vaccinated against Covid.

**Discourse:** One question. Is this question from the lady intended to test the prisoner?

**Discourse:** A test survey.

**Discourse:** A test of a possible vaccine that is reportedly being developed.

**Discourse:** The vaccine will be tested on Brazilian prisoners.

**Discourse:** Exactly.

**Discourse:** So, I... If I have the opportunity, I'll accept. Yes. I don't know when that will happen, probably if it will even happen to him. I hope to be here. But if I am, you can count on me.

**Discourse:** No, no. It's really asking about... It's like... What we were talking about with you, asking for the third time, is doing research on the vaccine. If you accept taking it. Mediator. If you think this is a good thing. Or if it's a bad thing. We would be the last phase of testing. If it's for health, if it's to improve things, and if it's approved by everyone. I can call you "you." Of course. Even if you say that it will be tested abroad. It will be taken. It will come to us too if we accept it.

**Discourse:** Yes, I think so. What would the guarantee be? None. Mediator. All the guarantees that the regulations give regarding the effectiveness and safety of the vaccine. The guarantees are... When we present a phase three study, we present it under very strict regulations. They are strict regulations from Anvisa (Brazilian Health Regulatory Agency), from CEP (Ethics Committee). There are several regulations from the Ministry of Health. Because otherwise, the people who are punished are not researchers. We need very rigorous approvals. With a lot of responsibility to develop research. It's because, for example, when I arrived here in the first year, I took the flu vaccine. It gave me a reaction. I was sick for about three days. I was fine and suddenly I got a fever, I felt bad and all that. I don't know if it's because my immunity dropped, or was already low, that this reaction occurred, or some component. I don't know. Because there is also the issue of allergies. Sometimes a person is allergic and doesn't even know it. For example, I am allergic to Benzetacil (penicillin). I can't take it. And before you take Benzetacil, you can, of course, get a test done outside. Tests, right? Tests to see if you're allergic. If you'll have any reaction. Your body will react in some way to that composition that's entering your body. So, only in terms of that, in terms of benefits, it's great.

**Discourse:**  I also got the vaccine, like him, and it caused a reaction that made me catch the flu. I had nothing wrong with me. I got it and I caught the flu. I got a fever, a runny nose. I got it and I caught the flu. I got it so I wouldn't catch the flu, and I caught the flu. Now, I don't know if, according to the research, if you suddenly have flu symptoms, but that's how it is, that's not how it is. Because I was feeling fine, I got it and I caught the flu.

**Discourse:** Then, sometimes, comes the resistance. The resistance.

**Discourse:**. Yes.

**Discourse:** In my case, I never had a "no" reaction. Thank God.

**Discourse:** But there's no way to know, if you don't take it, how will you know if the vaccine is safe if you haven't taken it yet, if it hasn't even been, how do you say, tested on the population or even on prisoners? You're being a guinea pig, in fact, for you to test it, in fact. That's the most well-put way to talk about a vaccine. If you're seeing the third reaction, you want me to be tested, as you said, first in the laboratory, then on animals, then it will come to people, right? And the people are us. Only by taking it will we know the reaction. That's it, man. That's the risk we run, but it's for our health.

**Discourse:** It's very important for the whole world to get vaccinated, and regarding the TB vaccine that 's being developed, in my opinion it will be very important because it will prevent this disease for the whole world.

**Discourse:** Could you give us more specific information so we have a better idea, and also reassure all of us, especially since this scaredy-cat here doesn't need to get vaccinated, you know, to see if she can at least get one vaccine?

**Discourse:** I was curious to know about its composition.

**Discourse:** Ministry of Health, right? They should express their point of view to the general population like this.

**Discourse:** Yes, and I would make my children take it. He didn't want to take the COVID vaccine, and I said, "Go ahead." And I made him take it. Because it takes so long, you know, all that progress to do a study, to make the vaccine, and sometimes people don't want to take it, and those who don't take it can harm others, even those at home. It ended up bringing the disease from the streets to us, as has been said, but the vaccine is very important for everyone.

**Discourse:** Because, for example, I saw on television the other day, and there was a dengue vaccine. But the response was... They're not getting it right. So, I have the science, I saw it, everything was there, but in any party there wasn't a response of going there to get vaccinated. So, it was still low, because they have, right? A calculation, a percentage that a vaccine will be successful. Because they didn't have that response. So, they were still calling for... It's precisely in this part of raising awareness among the people that's lacking. Because there's a vaccine? Yes. There are... The professionals have it, but the world is going downhill because there's a lack of information. And leaked information, like you're discussing here now, you have no idea what the dividing line is. Because, at least, I've never even been interested in this subject. Because I put on my glasses and then I look the other way. So, we are informed about this subject.

**Discourse:** I think it would be very important because the majority of the TB population is inside the prison. It's mostly not male. Many arrive who have it, sometimes they don't know they have it, others have it but don't say anything. So it contaminates the prison society. This also applies to women. Because many times when they arrive here, sometimes they go straight down to the general population. Other times they don't because they were informed in the infirmary. But we don't understand why. Because they don't say what they really have. So it can pollute the prison society. I think it would be very important, especially inside the prison.

**Discourse:** Because that's how it is, folks. I think the possibility is much greater outside. But inside the prison it's even greater because it's a closed environment, an environment with little space. It's not like that outside. Outside the world is big, you can take precautions, you can go to a health unit. You can get hospitalized, you can take precautions, you can take care of yourself. Inside the prison the situation is a bit more precarious. Because we don't have much assistance, not much, just like we can't take precautions going outside. Waiting for an escort takes a long time, months, years. So I think it would be very important both outside and inside the prison. But especially inside the prison. Because it's a closed environment. You can't take precautions, you can't know if it's the person arriving from outside. Because there are many people who have been here for many years, then a recent arrival with problems comes along. That person who has been imprisoned for many years and has nothing wrong ends up catching it from the person who arrives. So there's a lack of communication, information, and foresight as well.

**Discourse:** The thing is, our immunity is low, which makes it even easier. So, the vaccine is very important there to prevent anyone from getting infected.

**Discourse:** Because the vaccine will emerge, there will be studies, the vaccine will be made, and we will participate. We will participate, we have to do our part. That's what we want. It's work. My children, my descendants will benefit. That's what we wanted, right? That's what we want.

**Discourse:** So, if I can participate in this, it would be good for us, and besides gaining more knowledge, it would be passed on to future generations.

**Discourse:** It would be good if the treatment were to prevent TB, but for prevention it would be someone who has already had contact with a patient who has had it.

**Discourse:** I think that I will participate in this study, which is important for humanity, because the vaccine will last forever, passing from generation to generation, serving your grandchildren and great-grandchildren.

**Discourse:** The vaccine isn't tested, right? What if it has a side effect and we die?

**Discourse:** I think that to study Covid, many people also did this study to reach a conclusion and to pass it on to society as a whole. So, I think in reality, it's the same thing, right? You would never want to do a test on me. Do you have any way that if it worked for me, it would work for other people too? And for those who have, like, the composition for me is always, for example, allergic to dipyrone, right? There's this situation with the medicati**on.**

**Discourse:** We're going to get the vaccine, and since we have the right to know, we'll get a fever.

**Discourse:** Because sometimes we find ourselves deprived of freedoms, because we have very low immunity, we are very vulnerable. And we become concerned about this . Because we... don't have access to healthcare.

**Discourse:** Because, suppose we get the vaccine, when we come back from the reaction. Maybe a very high fever, maybe diarrhea, maybe... And we wouldn't get the proper care for that. You got vaccinated, so you have care now. How long will it take? You 'll feel unwell, you'll be able to get care. That's when you'll have to have a fever. You have to have very strong diarrhea, you know? If you have that, will the security team, will the security team come?

**Discourse:** Many times we hear another ward calling. "Ma'am, please , here in that ward, the inmate is feeling unwell." And then, many times, "Now, wait, I just sat down." Like this. Now it's our turn. Sometimes we say, "Ma'am, please, you... Ma'am, here in the others... Please, she's feeling unwell. Wait." So, the way he's treated is inhumane.

**Vaccines in Geral**

**Confidence in vaccine administration by healthcare professionals**

- Confidence in vaccines (benefits and safety)
- Prior knowledge about the vaccine
- Belief in natural immunity
- Personal experiences
- Lack of information
- Fear of side effects/risk of vaccines
- Protection of individual and collective health

This document presents the participants' literal quotes, which constitute the database for the analysis of perceptions regarding Confidence in vaccine administration by healthcare professionals and Confidence in vaccines (benefits and safety). The following transcripts detail the relationship between Prior knowledge about the vaccine versus Belief in natural immunity and Personal experiences. The statements reflect the impact of Lack of information, the prevalent Fear of side effects/risk of vaccines, and the underlying concern for the Protection of individual and collective health within the custodial setting.

**Discourse:** Very reluctantly, people don't know anymore with so many vaccines. Vaccination all the time, vaccination. Then everyone gets reluctant because they're saying that if they don't get vaccinated, they'll catch the virus. Everyone gets vaccinated. And nobody really knows about this vaccine thing.

**Discourse:**. The last vaccine, I didn't even know the name. They just said it was the new, modern vaccine.

**Discourse:** I didn't get it. It's a no-go for visitors. At first I still got the meningitis vaccine. That arm vaccine, the one everyone got, the one with the mark, I didn't get that one. Because it was in the middle of the sea and there was no way. But later, when I ate, I started to keep track, I'll register it later. You understand? But at first it was fine. Meningitis, rubella, vaccines like that, all that stuff was fine. But now there are so many vaccines that people are already wanting them. You understand? People don't even know what they are. First the Covid vaccine, it was one. They made another one, then another one, then another one. I've already had six vaccines. I'm not getting any more.

**Discourse:** Yes, in a year I think that's it too. Remembering, in a year and two months, I think I've already taken about five of these Covid vaccines.

**Discourse:** This vaccine was the fifth one I took. It was the fifth dose I took and I had a reaction. And most people, right, everyone took it too. It was said, right, that whoever hadn't taken it wouldn't have visitors. And then everyone took it. And I had a reaction. And quite a few people had a reaction.

**Discourse:** I also noticed that after the vaccine, this last one, I had a lot of complaints of chest pain. I never had many complaints like that about chest pain. Because I go to the cell almost three times a week. I go, the nurse gives some medicine. Then I ask who's in pain, who's feeling anything, right. Most people feel something, right. And it's normal, right. I always recommend, look. Take it, go to the yard, do some stretching, right. Do something, I have... Luís is 62 years old, you know? He has back pain, so I take him to the yard almost every day. So he can do some exercise, you know, some stretching. But the chest pain started quite early... Look, if there isn't one per cell, there are two who complain of chest pain. Sharp chest pain. Pain in the middle of the chest that goes up to the neck. Some go down to the arm. That's what I get a lot of complaints about.

**Discourse:** They said that every year there would be a vaccine for us now.

**Discourse:** She said there was an outbreak on the street that... Even they had dismissed visitors. Then there's an outbreak on the street. The visitors were bringing it from the street to the cell, because it was for vaccination. So...

**Discourse:** I'm very careful, the news isn't informing me. I'm very careful, I'm just paying attention. Especially here in Rio Grande do Sul. How am I going to say it? I don't know how to say it now, but... Oh, there was an outbreak of H1N1 flu. You understand? Vaccine... I don't know what. Do I mention names? I don't know, they put difficult names on the vaccines... But most of the time it doesn't... It doesn't get in here?

**Discourse:** Well, sometimes it doesn't get in, right? No, it doesn't get in here. How can I repeat this? Oh, now I remember. Like this last one, we didn't have any cases, right? Not a single case of this disease from the last vaccine, right? We didn't have any cases, in the whole prison, of anything. We didn't have any deaths, we had none here in the modular unit, from Covid. Or did we? No, right? None... Here in the modular unit, none.

**Discourse:** From inside here? Yes. Those who live inside here. It's perfect. One of the prisons I've been in that cares most about the infirmary is here. Here there isn't... You can see, I have a medicine box there that I have almost all kinds of medicine. When someone comes in... Oh, they're feeling sick here, man. Go quickly, some of them have... What do you mean? Tachypeptic. Right? The guy doesn't need to be called anymore, just lay him down here, he took his medication today, how is he doing? He pays for the medication every day, morning and afternoon, for them, they don't even keep it, nor pay for it. So, he took his medication, everything's fine. Oh, no, I took it today, leave him lying here, he wakes up, give him his medication. And that's how we go. But it's great, the infirmary is great.

**Discourse:** One question regarding the visit he mentioned, about the doses on the street, right? On the street they keep track of how many doses you took, right? Like I also took three or four on the street, if I'm not mistaken. And here, no, it seems that, I don't mean here, in this

**Discourse:** At home, more generally. It seems like they pay for the vaccine by the needles. There's no one who, if you've already had three needles, you're either going to take this one or you're not going to take it at all.

**Discourse:** When it comes to tuberculosis, we've already had two or three lectures, I think, on tuberculosis, other diseases, hypertension. From what was said in the lectures, tuberculosis is more prevalent in prisons. I understand that tuberculosis is the first thing to be treated, but COVID comes from the streets. I believe that with COVID we have more community treatment. I believe that with COVID we have more community treatment than with those who were on the streets. There's less contact with more things, but tuberculosis comes from outside the system. So much so that people who are undergoing treatment here leave and don't continue.

This last vaccine had several reactions lasting two to three days. Fever, body aches, and sweating. It was a booster shot for COVID. Some vaccines have side effects. Several people... This should be optional, right? It had to be optional, not mandatory. You understand? Signing the form is fine. If there's a problem, you go there and... You understand? Not that everything is mandatory. One thing you do... There are people who don't even have more problems. The effect of the other. Not every body is the same. Each body is different. Each one has a body, a blood system, blood type. It's all different. When you take a series of people there, everyone is different. It's like a fingerprint, everyone is different. There's no one the same.

**Discourse:** During the Covid pandemic that happened recently in the prison system, everyone was vaccinated. Everyone. Even those who didn't want to were vaccinated. They had to be vaccinated. It was a priority to be vaccinated. I see that although we are men, 30 years old, some 40 years old, others older, but many people also have a fear of vaccines. They don't want to get vaccinated at all. They dig their heels in and don't... They're going to get vaccinated and they don't, you understand? I don't know how to explain it. It's not my case, you understand? But within the prison system, there are people who don't accept it, they prefer the pill to the injectable. Because they have a phobia or are afraid of vaccines, you understand? But for me, in my case, I think it's better because the vaccine has a faster effect, you understand? Whether we have pain, a fever, or something. Any injectable medication has a faster effect.

**Discourse:** Like he said about the vaccine, here we have many brothers who really have a phobia of vaccines. So, that doesn't apply to me either, but like, even because of the doctor, the nurse who is giving the vaccine, because sometimes they don't have the responsibility, you understand? To administer the vaccine there. And that's why many people abstain. I say, I'm not going to get vaccinated because of how the vaccine was given to my brother. You understand? How it turned out. I think it wasn't in the right place there. As we've already seen, we've had cases here in this unit where an injection was given to a friend and the friend ended up dying. You understand? So, like, that's what prevents many people from getting vaccinated, you know? They really prefer medication in pill form.

**Discourse:** Absolutely, absolutely. Because, not to diminish their work because they are also professionals, but I believe that since the demand is high, there's that thing, I don't know, because there are so many people and so on, then that fatigue, what they're going through personally, you know? I don't know if it's because of that, but I believe it would improve a lot if a professional from outside came in, you know? To give that attention to the unit, you know? It would improve things a lot.

**Discourse:** It protects, right? Yes, it protects. The issue of... If I have an inflammation, then I'll get an injection, I'll get an injectable diclofenac, at that moment it will meet my need, it will be there, you understand? But... Depending on the application that was given, over time there may be a... How do you say it? A side effect, right? From the medication.

**Discourse:** It's mandatory to take it. Even if you refuse, if you choose not to take it, you can lose, lose your visit, as we have already been threatened with losing visits. Who wants to lose their visit? We who are deprived of liberty. We don't want to lose, so that's why it becomes an obligation, you understand?

**Discourse:** This level of vaccine research. Well, I'm on vaccine treatment, I don't have a spleen. Every month I need to be there. Once a year I have to go to the "Tropical" clinic to get a vaccine because I don't have a spleen, for recovery. I need to get this vaccine so my immunity doesn't drop. If my immunity drops, then tuberculosis comes, then the flu comes. Any cold I catch, then I say, I have tuberculosis again. If I catch a cough, I have tuberculosis again. This vaccine, getting a vaccine, I think it eliminates all the person's need. That's it.

**Discourse:** Eliminating the need would be good, right? A vaccine for someone who is taking medication, which, according to you, medication when it's supposed to be taken at the right time, doesn't come? It's better to get a vaccine that lasts a month.

**Discourse:** Never.

**Discourse:** No, no, they are units. Unit of CDPM2, which had a larger technical staff to attend to people, because here they attend to 10 people a day, there they attend to 20. So there were more nurses, more doctors, you understand? They gave a lecture there about Covid, you understand? Because there were a lot of rumors that it was going to kill you, that if you were vaccinated, you would get Covid, you understand? That they were bringing the vaccine from outside, the disease from outside through the vaccine, you understand? That also caused a lot of phobia in people, not wanting to get vaccinated, you understand? But they gave a lecture there to end this rumor that was going around, saying that the best way was for everyone to get vaccinated and that way we would be preserving both our lives and the lives of our family who came to visit us.

**Discourse:** Good morning, actually when they come to vaccinate us here in prison they don't inform themselves, they don't inform us. They call people, and the vaccine isn't just a simple matter, like, "Look, there's going to be a vaccination for you tomorrow for this, that, and the other thing." At a certain point, they arrive at the prison, call the cell block, say you're going to get vaccinated, and they don't explain the situation with the vaccine. They say it's for the flu, they say it's for Covid, and for other diseases we don't know about. We have no way of knowing...

**Discourse:** I think it would be important to get vaccinated, I think all the vaccines they develop, right? For diseases, it's important. I think a vaccine for tuberculosis would be very important.

**Discourse:** Because I myself am afraid of catching tuberculosis. Because, especially here inside the prison, there's no way for us to escape the problem, right? Because sometimes, the cellmate who's living in the same cell or cell block, on the same side as the shower, the side with the sun. Whether you like it or not, you're going to have direct contact with them, and we can end up getting infected. This is a problem even if the unit treats it, even if the SUS (Brazilian public health system) provides the correct medication, but it will leave you a little more, it will diminish your health capacity. So, whether you like it or not, in the future it can bring a bigger problem, even if you get treatment, you will be a little weaker. So it would be very interesting if we introduced a vaccine for tuberculosis. I would take it.

**Discourse:** I wanted to talk once again about the situation that people mentioned here just now. I got the COVID vaccine, but I didn't feel anything. Some time later I came

**Discourse:** They had to take the test, everyone took the test and it showed that I had it. Okay, but what happened is that I arrived here and was forced to take this vaccine. On the day of my hearing, I could only go if I was vaccinated, and since I hadn't done anything and I knew I was going to leave, I had to take it. Later they said that if I was acquitted, I wouldn't leave, because if I didn't get the vaccine, I wouldn't go. I got the vaccine, I didn't leave, I felt bad, my eyesight got bad, and I'm still bad today because of the vaccine. So I think that if I hadn't taken this vaccine, I wouldn't have lost my sight. This is the last one and it's over. Until a few days ago I was taking it and it hasn't finished, so when is this vaccine going to end? That's my opinion, that's what happened to me. I felt very bad, I even thought I was going to go blind. And my eyesight got worse; it was something that perhaps brought the world back to life, and after that, it damaged my eyesight.

**Discourse:** I'd like to know about the new vaccine. Since I've already had it, my lungs are affected, so it's like a vaccine that would expel the virus, because COVID-19, a vaccine was created for you to fight the virus, like if you haven't had COVID, you'll receive the virus and fight it head-on, in the case of TB, this vaccine, I don't know how it works. But it would help, but in what way? In its development, the person doesn't suffer the after-effects, right, because they had it, so how does this vaccine help? How would it help with the suffering of someone who has already had it or who might get it? What would its development entail? How would it help? Thank you.

**Discourse:** I'd also like to know how this vaccine was developed? Generally, vaccines are developed to increase the body's immunity to that virus or bacteria, right? Was it developed in the same way? Or does it seem to be developed in the same way?

**Discourse:** Because, as I've already said before, I felt harmed by this whole thing, so a question I want to ask is, if it's a single dose, will I have to keep taking single doses frequently? I want to know because it says it's a single dose and I shouldn't have to take another one. And I also want to know what the symptoms are because I've already had some vaccines and I don't want to be affected.

**Discourse:** This question about the purpose of the vaccine is very much about that. The only people who wouldn't participate in this regard are those who are healthy, right? Those people for whom it doesn't affect them, because COVID, after taking many lives, didn't have an effect on everyone, right? I didn't even catch COVID, but it was just a normal flu. So, 30% of those people can't participate because they have good blood, good health, so sometimes they're even afraid to get vaccinated. And because of the strength of the blood, for example, like my mother, my stepfather was HIV positive and my mother, who died, I cried a lot, she cried and cried, for example. And so, my mother doesn't have anything, my mother's blood is clean, for example, my mother is beautiful, my mother is 64 years old, so that's very good. I'm a healthy person, but I'm also often blocked, and the psychological aspect gives more strength, it lets us wander, so the psychological aspect gives, it goes to your head, it sabotages you, so I think like this, I have this position in my mind, and these diseases, they don't affect my blood, so I'm not going to get vaccinated because my blood is good and I know that.

**Discourse:**  It was God, it was God who saved him from catching it. That's my thought.

**Discourse:**  High immunity.

**Discourse:**  It was God who helped him.

**Discourse:**  High immunity.

**Discourse:**  You have to take it, the researcher's mind isn't going to be there, making vaccines, making vaccines, making vaccines. When making the vaccine, it's not going to be to harm the person, it's not going to be done for the good of others, you understand? For me, vaccines are necessary.

**Discourse:**  Vaccines, as my friend said, are also necessary. I've taken all the vaccines, you know. I've taken them all. I think vaccines are necessary.

**Discourse:** If I were to get vaccinated, I wouldn't be doing this.

**Discourse:** They regularly administer vaccines to us; they don't disappoint in that regard. They've already given us the Covid vaccine, then there were booster shots, and they've given us flu vaccines several times during my time here. So, regarding vaccines, they don't fall short. Whenever there's a campaign, they come for us too. There's no shortage here. Moderator: And do you accept vaccines? Normally. Moderator: What's your experience when the vaccines arrive? Do you think it's good or bad? Look, we see that people who don't get vaccinated already know where it's going. So it's good to get vaccinated. Vaccines are always a good thing, as long as they've been tested. Because usually they're tested outside of here. When they come here, a specific public has already been vaccinated. So why shouldn't we get vaccinated too? Always approved with this vaccine. And here they don't let it run out.

**Discourse:** I think they're to prevent diseases, right? I think it's very good, because in the past we didn't have access to these things. Today we've been helping you all a lot. All kinds of diseases, treatments, very good.

**Discourse:** I think it's really about prevention, you know. So much so, because, you know, I have a child. Since my son was born, he's been taking medication. It's important that we take care of this in the past, because the future is coming. It's also important that people study for this and specialize in this area for the prevention of humanity, you know. Our health is important. It's very, very important.

**Discourse:** So, I think there should be more vaccines for people who have intimate diseases, you know, gynecological ones. Because they are very often treated... It's just pills. I think the vaccine gives a faster result. You understand? I think there should be. Especially the penicillin vaccine. For us to get a penicillin vaccine here, it's rare. Very rare.

**Discourse:** A vaccine will also help many people who have relationships with these diseases, especially with HIV-6.

**Discourse:** Like, the COVID vaccine. There are many people who haven't taken it. I worked on the front lines, you know, I didn't catch the virus. But it was very sad. But there are many people today who haven't taken the vaccine because... I think because of prejudice. And the vaccine is important. This was done... Many people lost their time there to make this vaccine. And it worked. So it's to help cure, right? People have to have that perception. That it's not out of malice, it's because... It's not out of malice.

**Discourse:** Many people didn't take the COVID vaccine because they heard rumors... That it even turns into something like that. And that... Even after centuries. After centuries, we've been thinking about something like that. Without a doubt. Friends, why didn't you think about it? These are uninformed people, I think that's the information everyone has.

**Discourse:** I didn't take it because they said that if people are in a very fragile situation, it could cause a bigger problem. My aunt took it and her arm became paralyzed. My daughter took it and lost her sense of taste. My friend took it and got pain in her leg. They say that this disease, this vaccine they made, was caused by the Chinese, that they paid for this epidemic. I heard several rumors, and I was in that situation. They said it was the end times, that this had to happen because God is coming. So I heard several things about this vaccine, and I simply didn't take it. I was afraid, I was scared. I thought about several things at the time about this vaccine that is supposed to kill, that it's so strong that even if the person is of a certain age or young, or if they have some illness, it leads to death. Because many people who took this COVID vaccine, I saw people who were healthy and in good health die. They took this vaccine and it has already affected other health problems in these people.

**Discourse:** Like she just said, "We're going to die, it's a risky thing, it's the end times, it kills, that this vaccine..."

**Discourse:** "The vaccine is meant to kill, you understand? That's why I didn't take it, because I came to the other world andI see that this vaccine is going to be so strong, you understand? And also because even Bolsonaro didn't want to take it, not even him, nor his family, he just made a team saying he had taken it without taking it, and then he didn't want to take it, and neither did we, you didn't take it either.

**Discourse:**I took all of them, thank God.

**Discourse:** I only took the first dose because I had a reaction to the vaccine, then my body felt very bad, it got very hot here, I felt sick."

**Discourse:** I see that the vaccine's purpose is to make our bodies immune, that's why I think it's important. I took all 4 doses of the COVID vaccine, I took the MMR vaccine, I had the first reaction to the COVID vaccine, but maybe, the first dose, right? I had a reaction, I won't explain. So I see it this way, regarding vaccines, also vaccines during pregnancy, I took the children to get vaccinated, I think it's important to have prevention, even now, even the vaccine for cervical cancer, right? Children take it at 12, 13 years old, so I think it's important and there should be more explanation in these health systems, and we have to have a multiplication to really remove this, because since the vaccine came out, there were people who didn't want to take it, there was even a vaccine revolt, so it's a lack of clarification in this situation, because I shouldn't take it, I never wanted to take it, besides seeing the same information that I didn't accept it, the next day I'll take it, I think it's just another issue, but for me it's super important. I don't even know if I got the tuberculosis vaccine, but it's very good, right? I wanted to know.

**Discourse:** I got the vaccine, had a reaction, but I kept getting vaccinated. But I got more illnesses, I think the vaccine is very important for prevention.

**Discourse:** I got the vaccine, all 4 COVID vaccines, and I know it's for prevention for humanity. I think the people who talked about the vaccine were right, because it was very important for the whole world to get vaccinated. And about the TB vaccine that is being created, in my opinion it will be very important because it will take care of and prevent this disease for the whole world, it's very important.

**Discourse:** In the case of COVID, which they are not taking, this one here felt feverish, everyone felt feverish, saying that one of the vaccines was stronger, those who took it felt feverish, headache. For me, I think it varies from person to person, you understand? Because when I took mine, thank God I didn't feel anything.

**Discourse:** Even children get a fever when they get vaccinated; it's a reaction to a vaccine.

**Discourse:** It's very strong. The truth is, the COVID vaccine is actually the virus itself, transformed into a vaccine. It's there so the body can learn to fight the disease, but everyone has a reaction. You know, something I found interesting when I came here? On the first day, they did the rapid tests, which is very important. But it's also important to see that every three or six months they call you back to repeat the tests.

**Discourse:** That's what they did when I arrived the first day. But if you know about the vaccine issue, I think there's a real lack of information, because many people sometimes don't get vaccinated because they don't understand the information. Sometimes the negative information comes first because it's something they might say they won't get vaccinated. My mother, for example, didn't want to get the MMR vaccine because I don't know who told her that someone had been killed recently and wouldn't be paying her pension anymore. My mother was very resistant; it was a lot of confusion. You see, wrong information makes things worse. So, just like there's a breast cancer awareness campaign to be well publicized, we're doing very well here, so the importance of having mammograms is being addressed, you know, that pain is ending, this ignorance of ours. I believe that the vaccine should be promoted more, the importance of how the body reacts, how the body reacts, and thus know well what the vaccine does.

**Discourse:** Regarding the vaccine, in general, the most accurate way we have of providing care is that everyone is called to get vaccinated when they have the flu, or for COVID, so on.

**Discourse:** But the vaccine doesn't mean you won't get it; you can get it, but your symptoms are less severe.

**Discourse:** The only information we have is from television, so yes, sometimes you can't get it because there's no accommodation, so there's no information at all. They just arrive and say, "It's the flu vaccine, so get vaccinated." That's all.

**Discourse:** I'll share with you from the beginning of COVID-19, the buzz that it was going to be a pandemic. I also worked abroad, and it started to emerge that a million people had died, and people were getting out of trucks with a bunch of bodies on top of them. I said, "I'm not going out to work anymore, I'm not." I already thought like that. I followed the news. I'm not from the state, I'm from the state of São Paulo, really. I'm not going out, because those who go out on the streets are the agents, and the virus will come through them. I don't want it, I want the vaccine to come. But it took a long time. And I thought, if I die, my family will know. So when the second, third, fourth dose arrives, I don't even care if it's AstraZeneca or another brand, I'm going to take it and get immunized.

**PDL Autonomy**

- Individual autonomy built within a group context
- Coercion
- Group identity
- Freedom of choice
- Repression by external agents and circumstances
- Fear of having their rights violated

This document presents the verbatim statements of the participants, serving as the database for the central coding of Autonomy and its sub-codings. The transcripts that follow reflect the perceptions, experiences, and opinions of individuals deprived of liberty on topics such as health, vaccination, and the right to choose in the prison environment.

**Discourse:** But the health workers don't give them out, the nurses don't give them out masks. So, the on-call staff also talks to them. They have to use them there, it's very... Regarding medication, like he said, I have a little box there with all the medicines. You can take 10 to the ward per week. I take five, four. The rest I can control internally. I go twice a week, I see who is losing weight, who has symptoms of tuberculosis. Then, I put them there, I take them to the nurse on Monday. Look, we can do sputum tests on these people here. They have sweating, they have a fever, so that we don't have a tuberculosis outbreak in the ward, in this part, there's nothing to say. I've always been like that, you know. As much as possible, you know. As much as possible, also, because there are several wards. You can't just say there, oh, let's go to this ward, but let's put the 20 that are there. So, we put them as we can, you know. So that we understand this, too. One guy was there, he got tested, but it didn't show up. He got tested again, it was drying up, it was really strong, it wasn't drying up. I insisted, no, it's impossible. He went there, they even tested him, we isolated his cell. And then, we went to see the others, taking extra special care. Just to see the others, you know. To see if they were going to show any symptoms so we could catch them early and test them.

**Discourse:** A question regarding the visit he mentioned, about the doses on the street, right? On the street they keep track of how many doses you've had, right? Like I also had three or four on the street, if I'm not mistaken. And here, it seems that, I'm not saying here, in this place, but in general. It seems that they pay per vaccine by needles. There isn't a rule, you've already had three needles, you're going to have this one or you're not going to have it.

**Discourse:** As I said, I've been here for eight months, I had all my vaccines, right? All of them, and I had to get them all again, right, to be able to receive visitors. I presented my vaccination card. I had them all, and I presented my card. Everything was in order, and I had to get the vaccine. Otherwise, I wouldn't be able to receive visitors.

**Discourse:** But like with the Covid thing, they gave a dose, a new dose. Like you said, the vaccines come from the Ministry of Health, right? There are health posts on the street too, so why aren't people vaccinated on the street, but prisoners are? Because two years ago, when Covid emerged, there was a certain number of vaccines, like our people, our wives, took the three vaccines and then more. And after we took the three, we've already taken about five more doses. And why aren't people vaccinated on the street too? We end up feeling like lab rats, so they're testing the vaccine on us.

**Discourse:** If it's not showing on TV that there's that epidemic again, that they're only testing us again, then, you know, we talk here, oh, we won't receive visitors. Oh, so-and-so won't get tested, and the whole gallery will be without visitors. So it's kind of by force, right? It's true that they're forced to get tested. Of course, everything that's good for us, as he said, is a suffering for the family to come here, you know? Hmm, hmm. Right? We're the ones who pay for the mistakes we made. That's it. We're the ones who did it, you know? Then they come, but we didn't pass anything on to them, you know? It's complicated.

**Discourse:** I think everyone already feels comfortable here, because the conversation started off on the right foot, asking who wanted to and who didn't, and what it was, who it was. And it gave us the chance to say, "Do we want to stay or not?" I think that's it. Now, the part I think is right is that everyone listens to whether they want to or not. I think the conversation always starts right when one person asks if the other wants to listen or not. I think that's it. That was the most...

**Discourse:** Good morning, actually when they come to vaccinate us here in prison, they don't inform us, they don't inform us. They call us and the vaccine isn't just a simple way of saying, "Look, there's going to be a vaccination for you tomorrow for this, that, and the other thing." At a certain point, they arrive at the prison, call the cell block, say they're going to get vaccinated, and they don't explain the situation with the vaccine. They say it's for the flu, they say it's for Covid, and for other diseases we don't know about. We have no way of knowing...

**Discourse:** Yes, because we have a relationship, you know, a day-to-day relationship there, and we try to give each other security, so I believe that Speech: I won't want to, if I want what's best for myself, I want what's best for my neighbor, for my cellmate, you understand? So I believe he won't give me a suggestion outside the norm, you understand?

**Discourse:** Yes. It's because we are also, as I said, learning from their teachings, and we are also acquiring knowledge here, we are gathering information and everything, we will inform ourselves properly and pass it on to our family member, you understand? Knowing what we are going to be talking about. Now, if we don't know, there's no way we can ask them to do it, right?

**Discourse:** Yes. It's information, it would be good information, but sometimes there are cases and there are cases, right? Because sometimes, I get a vaccine here, I'm getting a vaccine, but my family member doesn't know that I got a vaccine there, so when they come to visit me, I'll say I got such and such a vaccine, I felt good, you know? And I hope you get one there too, it will be a suggestion and some information for them, my family member.

**Discourse:** I, personally, wouldn't participate in two ways. Firstly, I wouldn't participate because, as you yourself said, it's something that's being tested, something that doesn't yet exist, it's still being formulated. So, practically, what would we be? Guinea pigs. And, since we're signing this paper now, here, taking full responsibility, we won't gain anything, maybe, later on, we'll get one less day of remission, you understand? If a side effect were to happen to any of us in these tests, we would be harmed ourselves and that would be the end of it. But, if you were to provide the explanation, the paper here, which we're going to sign and read, and it stated that you would be taking responsibility for any eventual severity, any error that might happen to us, then yes, I would participate.

**Discourse:** But the vaccine is quite complex. When it came out there, COVID started, for example, and we were deprived of our freedom. It happened that the people who came to vaccinate us demanded that if you didn't get vaccinated, you couldn't go out for visits. It was something that, when COVID hit the system, created this situation that many people believe we live in, that we live in a country where people have free will, that we can make these choices of wanting or not wanting.

**Discourse:** But suddenly we were forced, even our conjugal visits were at stake, we had to accept it, even without knowing how it happened, where it came from, we had to accept it and many people were taking it and were getting headaches, it was getting worse, every body is different, right? So for some people the vaccine caused side effects, the vaccine did something to them, it's quite complex in this context... Today it's calmer, it's not mandatory to get vaccinated to receive visitors, it's not mandatory, but they are administering it constantly, including the COVID and flu vaccines within the system.

**Discourse:** Yes, I would agree to participate in this research, especially because vaccines are very important for our human society. We know that since the last century, many diseases have had their mortality prevented precisely because of vaccines, which provide us with defenses. I believe that with new research, especially on TB, which is very important in our society regarding our freedom and our social interaction, it would be very important and essential. We know that there couldn't be just one vaccine, but rather other types of vaccines because, as time goes by, we know that all viruses and bacteria are constantly mutating and changing. I am sure that there would be not just one vaccine, but constantly changes within vaccines, and with this research bringing adequate information for us to understand what is happening, I believe that we would agree to take it, and it is essential for us humans to avoid both mortality and the spread of viruses to maximize contamination.

**Discourse:** I don't know if I would be obligated to participate, but I would participate myself; I'm 44 years old, I have more knowledge.

**Discourse:** Yes, I would like to, I would accept being part of this research because I know it wouldn't just benefit me, but also the multitude of other people who need treatment. So I would be happy if I willingly participated, not just for myself, but for everyone in general. So yes, I would accept it.

**Discourse:** I would accept.

**Discourse:** I would accept it too, especially since I'm a smoker.

**Discourse:** I would accept that too.

**Discourse:** I would accept that too.

**Discourse:** I would accept that too.

**Discourse:** I would accept that too.

**Discourse:** I wouldn't accept it. I wouldn't drink it.

**Discourse:** I wouldn't change my mind. However, it's something that... It's something that affects us, it's family, children, mother and father. I've already lost my mother, I've already lost my father. So, I would never accept losing my children for anything in this world. Only their opinion. Or in their case. Then yes, I would take 50,000 vaccines for them. Apart from that, nothing else, I wouldn't change my mind, not at all.

**Discourse:** Would it be completely effective? Considering it's a serious illness.

**Discourse:** Would there be any contraindications?

**Discourse:** I really wanted to take it, regardless of the information.

**Discourse:** Will it work this time? Will it work this time? Will it be effective, efficient?

**Discourse:** If it were to be tested directly on us here, I wouldn't agree to participate. I would have to see the results first.

**Discourse:** We were going to be the guinea pigs.

**Discourse:** Laboratory mice.

**Discourse:** I would agree to participate.

**Discourse:** I'd accept, just to see how far it goes.

**Discourse:** Including regarding Covid, many people went without getting vaccinated. Many people here are not vaccinated against Covid.

**Discourse:** I wanted to say that the right to be heard is very good for us. When everyone enters this system, we lose many rights. This right to be heard is very good for us. So, as long as there is respect from both sides, it becomes easier for us to accept the treatment itself. So, everyone will accept it. I believe it's more than 80%. More than 80%. Most people want to know about their health. Everyone wants to go home. That's the truth. Respect is very good.

**Discourse:** I'd like to speak. You can speak. I'd like to thank you because this is a great gesture of attention. You researchers and healthcare professionals are doing a great job. This is very important for everyone, no matter what. So, I have to thank you. Whenever we can and you are there in some way to help, we are always willing to help.

**Discourse:** I also wanted to thank you for coming to the researchers who asked me if we also want to take the vaccine. Because if it were only tested abroad, and it arrived here we would be forced to take it. We didn't know what the reaction would be. We didn't know what its purpose was. So, thank you very much for coming here to the prison and asking again.

**Discourse:** The bottom line is that we know it won't happen now, but later. There's this whole new process. But the bottom line is one of gratitude for the initiative, knowing that in the new year there will be this treatment, this assistance that you help with here inside the prison, something that everyone is receiving. So, a very good moment and very welcome.

**Discourse:** This is very important, you know? Very powerful. And someone from there, spending a lot of time, clearing up some doubts, and we passing that on to those who don't have access to power. To be able to transmit what we've discussed here. That's it. For the good. For other people.

**Discourse:** I didn't know the name of the vaccine either. But, because they use capsule treatment there, right? So, I think it would be important. It would be mandatory for the population to take it, like, along with others, right? But it has to be indicated, they can take it, but that's how it is. There's fear, in this case.

**Discourse:**  Inside the prison, the situation is already a bit more precarious. Because we don't have much assistance, not much, just like how to protect ourselves going outside. Waiting for an escort takes a very long time, months, years. So I think it would be very important both outside and inside the prison. But especially inside the prison. Because it's a closed environment. There's no way to protect yourself, there's no way to know if it's the person arriving from outside. Because there are many people who have been here for many years, then a recent arrival with problems comes in. That person who has been imprisoned for many years and has nothing wrong with them ends up catching it from the person who arrives.

**Discourse:** So, can I participate in this? It would be good for us, and besides gaining more knowledge, it would be passed on to future generations.

**Discourse:** For me, if I were to participate in this study, I would already be aware that if one didn't work, I would have to take another and another until I found that "click" that I like, that would make the vaccine.

**Discourse:** Everyone, I can't say for sure or tell you because there isn't that communication happening, right? Here we are in a circle, there are explanations about various things, and people aren't here, but there is communication.

**Discourse:** I don't feel coerced because I know that if they're calling people to get that vaccine, it's for the best, to protect against something even more serious within this place.

**Discourse:** I think that's how I'm going to participate in this study, which is important for humanity.

**Discourse:** So, there's no pressure; whoever wants to participate comes, whoever wants to sign the agreement comes, whoever wants more information comes. If you want to see, if you don't want to know, there are many people here who give lectures on various topics... like, they're graduating, you know? Then we come to give lectures, it's interesting.

**Discourse:** Whether I participate in that study is my decision. Whether I go or not, even while incarcerated. Because it's about my health, right? So, it's a personal decision. I'll participate or not, I'll go because I feel safe. That's different.

**Discourse:** The agent is deprived of liberty. And many times, of course, it wasn't your choice to be here. You're already in a place you don't want to be. Now, participating in the things presented here at the unit, whether it's prevention, lectures, things like that, in that type of decision-making, it gives me the freedom to choose.

**Discourse:** I think that those deprived of liberty have this privilege, right? The privilege of being able to have this opportunity, not by force, nor by violence, nor by pressure of any kind. Especially because there are people outside who have never entered the system to have an hour of conversation with us, who, like you who are here today for the first time, are getting to know us. So, for those who have worked in the system, you know that nowadays, people, despite what they did outside to be here inside, they have dialogue, right? Dialogue, conversation, you know? To receive, you know? To talk to people too. And we understand that this is important for our lives, right? For our lives here inside this place. And also to pass on to the people who are outside, to tell them that we are free with our choice, right? The choice to be able to participate. And it's important to say that. It's a privilege, actually.

**Discourse:** We're saying that we live here in this prison. The administration here, the management here, they always work with us on this issue of letting us be free to choose. In a situation like this, for me, I wouldn't feel pressured.

**Discourse:** As he, the secretary himself, came here with us and said, "You are deprived of your freedom? Yes. But you are human beings. You have dignity, you have the right to respect. So, all these things, right? He said, 'This isn't a motivation, you know that, it's no use, right?' But you have respect, we, our society, you will return. You are still citizens. So, let's make it very clear that you will return and you are beings, you know?"

**Discourse:** And so, we also have the right, like any citizen, just not to be able to go back to our homes, right? To our families. But here there are studies, there are courses, there is care, which many times, in the area of health, we have psychological, psychiatric, dental, medical, gynecological care, we have this, this, this little time to go to the health center, to receive care here, to the extent that we can do so. For us.

**Discourse:** In some situations, you know, when we're in the ward or feeling unwell, the prescription always comes from the doctor. So, because it could harm our health, many times they won't give the medication we want, for a headache or something else. So, we prefer to see the doctor first, right? Before taking any medication. It's just a precaution for our health.

**Discourse:** Sometimes we have to help those who are in the cell, because there are people who live here, their families aren't from here, most of them don't have families, they don't have visitors, and sometimes those who do have visitors have to try to help.

**Discourse:** The only thing they called us to find out was why so many people were collapsing, of course they blamed us, saying we were taking too much controlled medication and collapsing because of that. Every time they bring up some issue, it's usually our fault. When they say there's a pandemic, right, the government says there's a pandemic, the first thing they do is cut off visits, and gate two, which we say is for receiving belongings, hygiene and cleaning supplies, that's the first thing they do. But, even before, when we had a period without visits, without anything, of isolation, but during that period of isolation, which was the period when everyone got Covid, because those who bring Covid are the agents themselves who go home and then, you understand, so they cancel our visits, our right, but they don't think about the fact that those who bring it are...

**Discourse:** And I would like you to take into consideration something that was mentioned here, which is to do it cell by cell. So that everyone, for everyone, has access to information.

**Clinical trials for a new tb vaccine**

- Trust in Science, Professionals, and Healthcare Institutions
- Fear of Experimentation
- Motivation (or Hesitancy) and Intention to Get Vaccinated
- Trust in Science, Professionals, and Healthcare Institutions

This document presents the participants' literal quotes, which constitute the database for the analysis of crucial themes related to Clinical Trials for a New Tuberculosis (TB) Vaccine. The following transcripts detail the perceptions of individuals deprived of liberty regarding Trust in Science, Professionals, and Healthcare Institutions, their Fear of Experimentation, and the Motivation (or Hesitancy) and Intention to Get Vaccinated. The discourses address the dichotomy between the hope of eradicating the disease and the fear of being treated as 'guinea pigs' in the prison environment.
**Discourse:** Ah, if it were tested on terminally ill tuberculosis patients and it worked, yes. But a vaccine, I'll give you this vaccine, you won't get tuberculosis, but I wouldn't take it.

**Discourse:** We had to see the test results, right? And you who produce the vaccine, you have to show them, right? Because the point is to know the results outside.

**Discourse:** There has to be approval, right? With approval, everything works, right? We'll arrive and say, let's vaccinate all the cell blocks there, right? It worked in 10, you understand?

**Discourse:** It's that most of the approaches, when it comes to vaccines, look, they call the on-call person, the health agent, it's the flu vaccine. Whether to take it, not take it, you'll sign the responsibility form there, you might take it, you might not. When the subject of visits comes up, the prisoner already pulls the brakes. So, I'll take it. There's a lot about this, this and this. It's the flu or Covid, it's only those two subjects.

**Discourse:** The question, I had already asked this question regarding the acceptance of new vaccines. In fact, new vaccines are constantly emerging, more diseases are appearing, and we are developing more vaccines. We need to know why these vaccines are necessary, how they work. I, and I agree with the other participant , will not accept injecting any vaccine knowing that I am not at risk.

**Discourse:** The system of those who make, manufacture, produce, and research new diseases that will emerge—but what are they researching based on? Not based on what already exists. So how can we accept a vaccine for a disease we haven't experienced? Television is showing that it will emerge, it's already emerging, I don't know if it's been tested, I don't know if it's been 100% proven, for example, HIV infection. Soon they'll want us all to get vaccinated, for HIV, for example, knowing that we have... I have a... my partner "Isis," and she has a clean test, there's nothing wrong, oh, but I'm going to get an HIV vaccine that will immunize me for the rest of my life. The same thing with tuberculosis. If I don't have it... I already know how the contact is, how you get tuberculosis, the first few days of the stage when it's strong, we have a certain reservation, no... not as they say, a... we don't have a prejudice, but we have to have our reservations, especially us health workers.

**Discourse:** Vaccines are based on studies, on research, but if it proves to be an immunizing agent, I would take it. I've already had the disease... I don't know what it's like. I know what it did to my body. So I would take it.

**Discourse:**  From my point of view, everyone has their own, right? I would accept the vaccine if we arrived there, with all the explanations, and said, "Look... Let's take the tuberculosis patients from the gallery, give them the vaccine, you'll see they'll become immune, and then I'll come back here to vaccinate you. You'll be immune forever?" That's something I would accept. But to arrive there and say, "Oh, you'll take the vaccine and become immune?" Then I would be strongly against it. But, like, if they came there and proved that the vaccine... "Oh, let's give the vaccine. It won't... Those with tuberculosis will be cured by the vaccine."

**Discourse:** And then tomorrow you get it again. Then I would be against it. But, like, to arrive there and prove, "Look... This one here has tuberculosis, let's vaccinate him, you'll see he won't... That his tuberculosis will be cured and he won't pass it on to anyone else, nobody will get it." Then I would agree, right? But it's a deal that would guarantee you'd become immune, even just because of the visit. Because many times you don't know, sometimes it takes two or three months to discover you have tuberculosis. Then, a little while later, your child or someone from your family comes along. You'll see, on the street, that you passed it on to your family.

**Discourse:** I would accept it. I saw in 2014, 2014/2015, a huge man, participant number 6 here, who It wasn't in that prison, no. It wasn't in that prison, it was in another one I was in. I got out of the prison throwing everything up, and then the guard just came back and said I wasn't coming back, that I was done with tuberculosis. He was the same height as me, like, he came out, but vomiting, like, I don't know, but his tongue was bigger than this table here, everything was spilling out, blood clots, all over the corridor, in about 5 to 6 months he was my size until the disease ate him up. Of course, I also think he didn't really push the treatment, you know, he didn't push it, he kind of abandoned it, he thought I'd get better anyway, and he never came back. That was in 2014, so I'm a guy who, by the way, already has this disease, a disease that scares me. Not for myself, but like, in a little while, let him catch it and pass it on to someone in my family, he was already coming here, you know? He's already coming here, actually, suffering there, coming here to live, and that's why I'm still bringing something for him.

**Discourse:** One of his questions was this: if we make a vaccine, if we make a tuberculosis vaccine for everyone to take, even those who don't have it. It's not because you saw someone die of tuberculosis that you're going to get the vaccine. If I saw someone get into a car accident, get all mangled, am I not going to buy a car? It's complicated.

**Discourse:** And the thing about us making it, if it were for the entire population to volunteer, not just for prisoners, for inmates, maybe I'd accept it, right? If it were for everyone, ah, so if it's only for prisoners, then no, right? Then it would be like using them as guinea pigs. But if it's a general matter, for the population and the prisoners, then I would accept it, maybe I would accept it...

**Discourse:** But like, accept it, maybe if it proved, if it were a simple question, if it proved, look, it's going to work, we don't have anyone who needs one, like, ah, there are a million people, we don't have anyone who needs one to see if it's going to work, I, in my way of thinking, I would say, ah, you can vaccinate me, it's going to work. But for sure, everyone has their own point of view, right? I think that more, like, if it were to vaccinate, I don't even often get there, most people get vaccinated because of the visit. Or if you go there and say, look, if there are cases in the yellow gallery, if there are two or three cases in gallery 1, something like that, right, if there are, there are no more visits. Then, they go there, they can't vaccinate them, they don't even want to know what the vaccine is, what it is, there aren't any more, you know, the thing, so, that's how it was, right? They don't even want to know what the vaccine is, if it will do good, if it will do harm, if it will affect me like I did. I was on my left side for five days, I lost all my strength, my arm was tingling for five days, I couldn't grasp any of that vaccine. I would try to grasp it with my left arm, it was the same as grasping it and letting go, until it wouldn't let go. I was there for five days, I had no strength in my left arm. But I went there and got it, I didn't know if it was what it really was, they said it was for Covid, that it was a single dose. But I didn't know if it would really work or not, but I went there and got it and I knew. Then, what if I catch a cold and they'll restrict everyone's visits?

**Discourse:** But like with the Covid thing, they gave a dose, a new dose. Like you said, the vaccines come from the Ministry of Health, right? There's also a health post on the street, so why aren't people vaccinated on the street, but prisoners are? Because two years ago, when Covid emerged, there was a certain number of vaccines, like our people, our wives, took the three vaccines and then more. And after we took the three, we've already taken about five more doses. And why aren't people vaccinated on the street too? We end up feeling like lab rats, so they're experimenting with the vaccine on us.

**Discourse:** I think the vaccination could start inside the prisons, but what he said there, if he came here, called everyone there, in a way, a consensus, how many tuberculosis patients are there, there are 50 tuberculosis patients, so let's take those 50 tuberculosis patients, if you agree, in a consensus, but not just come here without giving any explanation, just saying, let's vaccinate everyone for tuberculosis, but without giving an explanation It's like, 'Oh, this is going to immunize you for, like, six months, a year, a lifetime.' An explanation: let's test the tuberculosis patients first, to see if it really works. I'd agree to that. But to just show up and vaccinate everyone, just show up and say 'let's vaccinate against tuberculosis,' 'we have to vaccinate,' and then inject the vaccine into your arm, turn your back and leave—I wouldn't agree to that.

**Discourse:** So, I wanted to say, if we take this last Covid vaccine, which they said wouldn't happen anymore, right? Covid is here and it's on the street, but the street got it, but the vaccine we take—that's what everyone in the prison thinks about, because only the prisoners have to take it, and the street didn't. It's the same thing with tuberculosis, does tuberculosis only exist in prison? No, it doesn't, it exists on the street too. Ah, so let's test the vaccine in the prison galleries there." No. So, if it came, then we tested it, and it was proven to be effective, that it would help, everyone would accept it better, you understand? So, let's just test it in the galleries, you understand? The vaccine came, but only we took it, I at least didn't hear on TV that everyone is having to get vaccinated again, you understand? And only us. And then many are reluctant to take that vaccine. Oh, why should we take it again?

**Discourse:** A study is good. A study is good because the information comes to us, so we stay informed about what's happening, what's not. Worse is getting all the information and then having questions, not even understanding what's happening. You understand? So information is necessary. We need to feel comfortable, the information came to us, and the meeting was held, right?

**Discourse:** I think everyone felt comfortable here, because at the beginning of the conversation it started on the right foot, it started by asking who wanted it and who didn't, and what it is, who it is. And it gave us the chance to decide whether we want to stay or not. I think that's it. Now, the part I think is right is that each person listens whether they want to or not. I think the conversation always starts right when one person asks if the other wants to listen or not. I think that's it. That was the most...

**Discourse:** So I believe that information should be much more frequent about everything that happens in the population. About everything that happens in the medical field, about the studies they are doing, about diseases that are out there, that we don't know how to treat, we don't know how to react to, we don't know how to inform a person. Because just because someone is coughing a lot doesn't mean it's tuberculosis. So we need to have this information increasingly clear so we can help people who supposedly will have it, or have it, or be treated, or won't. And I think about that too. Because we should have much more information.

**Discourse:** We need to have more information. Sometimes there are people on the street, who have the daily routine of working, and they don't have the means to seek information. You understand? Because that's where it comes from. So sometimes we have more information here than the people on the street. And the more information that comes there, the more we will finish.

**Discourse:** This vaccine would be good because we live in a very close-knit environment here, we don't get much sun, we spend two hours sunbathing and our immunity is lower, you understand? I think a vaccine like this would be good to combat this type of vulnerability. We have this situation here in this place where we don't eat properly, we don't have much... We don't get much sun exposure, we spend more time indoors than outdoors.

**Discourse:** I would like to know about this vaccine, if you could bring, as he said, my brother, the package insert, in other words, the concrete information, the level of research you've done, if it was approved, 100% accurate. This vaccine here will be... You will take the vaccine, you won't need to take any more medicine or any other vaccine. Or else, you'll have to take this vaccine month after month because it's a treatment you have to go through, you understand? So, this information is very important to us.

**Discourse:** Many wouldn't want it because it involves getting an injection. But for those who really need it, it would be a good option, you know? Especially since we know that injections have a faster effect, right? So, if the information is kept confidential, if we know the effects it will have, if we know what we will really feel with it, it would certainly be good for everyone.

**Discourse:** I would participate, yes, I could even ask some of my siblings to do it too, because it would be a new experience and also because it would be good for me, you know? So, if it's good for me, I'll want to do it. And yes, I would be willing to participate.

**Discourse:** I think so. I think so. It would be very good for us because we would have more confidence in participating in a new study, of a new vaccine, knowing, because all the research will be done there. Knowing how the research is going, like he asked if we had already seen it, I've seen the COVID-19 vaccine, showing the egg-shaped thing, the little balls, the whole machine that compressed the vaccine, then the vaccine until it reached the person. I saw that part, only in videos, I didn't see it personally. But there, it was also written that it was 80%, it was never 100%, even so, half the population has taken it nowadays.

**Discourse:** I wanted to talk again about the situation that people mentioned here now. I took the COVID vaccine, but I didn't feel anything. Some time later, they came to test me, everyone took the test, and it showed that I had it. Okay, but what happened is that I arrived here and was forced to take this vaccine. On the day of my hearing, I could only go if I had been vaccinated, and since I hadn't done anything and I knew I was going to leave, I had to take it. Later they said that if I was acquitted, I wouldn't go home, because if I didn't get vaccinated, I wouldn't go home. I got the vaccine and didn't go away, I felt terrible, my eyesight deteriorated, and I'm still unwell today because of the vaccine. So I think if I hadn't taken that vaccine, I wouldn't have lost my sight. This is the last one and it's finished. Until a few days ago I was still taking it and it hasn't finished yet, so when will this vaccine end? That's my opinion, that's what happened to me. I felt very ill, I even thought I was going to go blind. And my eyesight kept getting worse. It was something that perhaps brought the world back to life, and after that, it damaged my eyesight.

**Discourse:** Also, I'd like to know about the new vaccine. Since I've already had it, my lungs are affected, so, like a vaccine that would expel the virus, because with COVID, a vaccine was created for you to fight the virus, like if you haven't had COVID, you'll already receive the virus and fight it head-on, like TB, this vaccine, I don't know how it works. But it would help, but in what way? In terms of development, the person doesn't suffer any lasting effects, right? Because they already had the vaccine, so how does this vaccine help? How would it help with the suffering of someone who already had it or who might have had it? What would their development be like? How would it help? Thank you.

**Discourse:** Because, as I said before, I felt harmed by this thing, so a question I want to ask is, if it's a single dose, will I have to take it frequently? I want to know because it says it's a single dose and that I shouldn't have to take another one. And I also want to know what the symptoms are because I've already had a vaccine and I don't want to be affected.

**Discourse:** I think not everyone would accept it, because you have to see, like the brother here said, many people had lasting effects from taking a vaccine, I myself was one, I took a vaccine a while ago and felt bad. Not everyone would accept it, but most would, I think not everyone.

**Discourse:** I would like to participate in the study, research it, and if, for example… If we had more people here, let's say I'm going to participate, right? And then, if there are more than us, will they call us there?

**Discourse:** You have to take it, the researcher's mind isn't going to be there, making vaccines, making vaccines, making vaccines. When making the vaccine, it's not going to be to harm the person, it's not going to be done for the good of others, you understand? For me, vaccines are necessary.

**Discourse:** Yes, yes, yes, yes.

**Discourse:** Vaccines, as my friend said, are necessary too. I've taken all the vaccines, you know. I've taken them all. I think, vaccines are necessary, if I were going to take them, I wouldn't be doing this.

**Discourse:** I would accept.

**Discourse:** I would accept too, especially since I'm a smoker.

**Discourse:** I would accept too.

**Discourse:** I would accept too.

**Discourse:** I would accept too.

**Discourse:** I would also accept it.

**Discourse:** I wouldn't accept it. I wouldn't take it.

**Discourse:** I wouldn't change my mind. However, it's something that... It's something that affects us, it's family, children, mother and father. I've already lost my mother, I've already lost my father. So, I would never accept losing my children for anything in this world. Only their opinion. Or in their case. Then yes, I would take 50,000 vaccines for them. Apart from that, nothing else, I wouldn't change my mind, not at all.

**Discourse:** Would it be completely effective? Since it's a serious disease.

**Discourse:** Would there be any contraindications?

**Discourse:** I really wanted to take it, regardless of the information.

**Discourse:** Will it work this time? Will it work this time? It will be effective, effective.

**Discourse:**The test already says, the vaccine can work or it can fail.

**Discourse:** The test may have side effects.

**Discourse:** The test is to find out what antibodies the vaccine will have to fight the disease.

**Discourse:** The vaccine can make you feel better or worse. If you get the Covid vaccine, you usually feel worse than if you got Covid, so you don't let the disease develop in your body.

**Discourse:** Will this vaccine be effective, will it work, or is it just hype? I was going to ask that, if it's not supposed to really work or if it's just for me to get vaccinated?

**Discourse:** I wanted to know if it was made to cure or to treat.

**Discourse:**I wanted to ask the following: if it would do what happened with the Covid vaccine, some people got sick after the vaccine, others nothing happened, nothing happened to me, many almost died because of the vaccine, will the effects be the same?

**Discourse:** It would depend on whether it had already been tested previously. If it already had a target audience, if it had already been pre-tested, what was the response to this vaccine? If there were any reactions, if there were any setbacks, let's say, in relation to this vaccine. No problem, no testing.

**Discourse:** If it has already been tested on the population, the vaccination, a medicine to have here, the treatment with people who come from outside, who can have these tests and more, that's great. But in relation to medicines, as you say here, not only here, but also abroad, it is a public calamity. Nowadays, the city hall, in the past, the city hall provided these medicines. Today we have difficulty buying them, we even have difficulty... It's like, how do you say, you have to chase after them, you have to buy the medicine. Because in the past it was donated by the city hall, today you can only buy it.

**Discourse:** One question. About the vaccine you are talking about, is it a vaccine that is supposed to be available, is it for This vaccine is a cure, for alleviation, or like the others that are only taken for prevention. What would this vaccine be? What would it be? Mediator. This vaccine would be a possible preventative vaccine to avoid contracting TB in adulthood. It would be immunization. It's prevention. It's to immunize beforehand, or to treat after the person already has it. Because sometimes, there are many vaccines that come out. The person takes it beforehand. But the vaccine is always for prevention. But I can't say for sure, because there are many people who already have it, many are coughing up blood. Mediator. This research group that I work with here, it does the diagnosis, the screening of people, it collects the sputum, it does the diagnosis, it does the examination, it does the medical examination, it does the X-ray and starts the treatment. So there is also a treatment. It treats for 4 months, or the time necessary for the person to recover. Also had an experience with this research group in Dourados. That's how you described it. That's how it's done here too. Today, the tuberculosis vaccine, as you've probably already been told, is given when we are children. But today, research is advancing. And this is research that many research groups are doing around the world for adults. For adults not to get tuberculosis. So, we are discussing this from the perspective of what would happen if you were offered this question.

**Discourse:** I would take it, especially because I've never had TB, thank God, I've never had TB.

**Discourse:** I think with myself, because I'm thinking about my health. About living with people who have the disease, or who don't, you know, or who get it again. There's a boy who comes and goes, this illness of his comes and goes. I don't know why. It's always coming and going. If he's here, he's already well again. That's what it offers you, what makes the difference.

**Discourse:** Even those who have already had tuberculosis get it again, right? That's the vaccine. It's another way. A mediator. Would you take the vaccine? And who would give you more security? So, who would give you more confidence? The health team here at the prison, your family, us, the researchers. Who would you feel more secure with to take a new vaccine? Number ??. With you, the researchers. You will test it.

**Discourse:** Yes, I think mainly because of the research, right? Because of the evaluation, right? This vaccine, because of the process it went through, the approval by the Ministry of Health, so that its application can be released, and that's what gives me security. After the research, the reactions, types of reactions. Because I heard you saying that when we are born, we receive a dose, right? A vaccine. It wouldn't be this one, it would be a new one. Is it a new one? A mediator. This vaccine doesn't exist yet. We are studying this vaccine. After research, right? After tests, right? It brings security.

**Discourse:** There will be research, the research will arrive here, and it will go through several implementation processes to be approved. It arrives inside the prison, it has already been approved outside, and then it will also enter inside the prison. And if it's coming inside the prison, it will be approved outside, right? Everyone used it and approved it. This is the entrance to the prison, right? There are people here who have already contracted tuberculosis, who have already been through the police stations, and it wasn't easy to cure them. But, despite the treatment, they are doing well, they are well cured.

**Discourse:** I see that any initiative to help the inmate, who is very needy, is welcome, plus another research project, like the one you are doing, with this initiative, in my view, will give us much more security and peace of mind to accept it. But in my case, whatever comes, I accept. It's impossible to dismiss. It's impossible to think that this won't affect me, because it will. And regarding the general point he made, I say, the prisoner, the inmate, is the person who knows best how to fend for themselves. Because I am learning something tremendous in prison. Because first aid comes from within us. I am a giver of life, I bring first aid. We have a function, a church, there's the title of pastor, we are the first to come to help. And there is this care within the cell, as we said, it comes from outside, and we have it, so this first aid comes from there. But there are situations that cannot be resolved. They require deeper care, more attention.

**Discourse:** Mainly about TB. If the vaccine arrives here, it will be approved, right? If the new vaccine won't cause a reaction in those who already have the disease, if there won't be a reaction. In my case, I've never had it. But if it won't affect those who have it, that's fine. Well, you know that a vaccine development process takes many years. It involves many researchers. No vaccine emerges in a month, two months, a year, or two years. The vaccine development process takes many years. Sometimes it takes 15, 20 years. And it's a rigorous process. First, studies are conducted, mostly at the laboratory level, then in animals, and then the public is invited to participate for a year. So, for example, the Dengue vaccine. This very group here worked on developing the Dengue vaccine at Butantan. This same group of ours. Several professors, you know Dr. Maurício, groups from the Faculty of Medicine here. They worked for many years on the Dengue vaccine. And we invited the public to participate in the third phase of the vaccine development, which was the Dengue vaccine. And we opened it up to the public. Many participated. The public participated. But we didn't open it up to the prison population. So, I'd like to know from you all. So, the vaccine was approved. The Butantan vaccine and the Taqueda vaccine itself, which is being given in Dourados now, in the municipality, I don't know if you know, was part of this group. It was made available to the public before it was approved. But it wasn't made available to the general population, we also work in the prison. It wasn't made available for the prison population to participate. If we develop a TB vaccine, make it available to the public and open this third phase to the prison, what do you think?

**Discourse:** That would be very good. In my view... It would be very good. Because we would have a vaccine for Dengue, COVID, it would be a good action... This vaccine would be good too.

**Discourse:** Absolutely yes.

**Discourse:** Absolutely yes. I think everyone, everyone in general, wants more clarification, everyone wants a vaccine, everyone wants to go home alive, preferably healthy. Here, peacefully, nobody wants to get sick. So, me and my brother, I've never seen him, but he's my brother, if he needs it, I'll help him. We have to help each other here. If a vaccine comes, with clarification, with everything in order, everyone will embrace the cause. Everyone wants to go home. Nobody wants to contaminate anyone. We don't want to be disappointments.

**Discourse:** Absolutely.

**Discourse:** Yes.

**Discourse:**  Yes, absolutely.

**Discourse:** Yes. That's what we're talking about. We want, one thing only, we understand that we're going to participate in the research and we could be guinea pigs. But we have to be conscious. We want to participate consciously. Moderator: What does "guinea pig" mean to you? What can you do? We can take the vaccine, if it's in our bodies, we'll be guinea pigs. Until it's approved. And it's all unsafe. Until then, we'll be guinea pigs, using vaccines. Many times there's no certificate of completion. And that could be it. It hasn't gone through several stages. We want that, clarification.

**Discourse:** So, from what you're saying, if you had enough clarification, you, the people who are here, believe you would agree to participate in the study.

**Discourse:**  Certainly. We might have some resistance.

**Discourse:** Someone could resist.

**Discourse:** But the vast majority would accept it; in all prisons, everyone has to go home healthy.

**Discourse:** It wouldn't be coercion. It wouldn't be coercion from the moment it was an invitation. So it wouldn't be coercion. For example, you agree to participate in the study, and so on. That's it. It doesn't oblige, right? It doesn't oblige.

**Discourse:** You wouldn't have that obligation? No.

**Discourse:** Including Covid, many were left without getting vaccinated. Many people here are not vaccinated against Covid.

A question. Is this question from the lady intended to be tested on prisoners?

Moderator: This question is being asked in six Brazilian prisons to ask the participants, the prisoners, if they would like to have the opportunity to participate in a test study, just as we open it up to society.

**Discourse:** A test study.

**Discourse:** A test of a possible vaccine that is being developed.

**Discourse:** The vaccine will be tested on Brazilian prisoners.

**Discourse:** Exactly.

**Discourse:** So, I... If I have the opportunity, I accept. Yes. I don't know when that will happen, probably if that will even happen. I hope to be here. But if I am, you can count on me.

**Discourse:** No, no. It's really asking about... It's like... What we were talking about with you asking for the third time is to do research on the vaccine. If you accept taking it. Moderator. If you think this is a good thing. Or if it's a bad thing. We would be the last phase of testing. If it's for health, if it's to improve things, and it's approved by everyone. I can call you "you." Of course. Even if you say that it will be tested abroad. It will be taken. It will come to us too if we accept it.

**Discourse:** Yeah, I think so. What would the guarantee be? None. Mediating. All the guarantees that the regulations give of the effectiveness and safety of the vaccine. The guarantees are... When we present in a phase three of a research, we present under very strict regulations. They are strict from Anvisa, from CEP. There are several regulations from the Ministry of Health. Because otherwise, the people who are punished are not researchers. We need very rigorous approvals. With a lot of responsibility to develop research. It's because, for example, when I arrived here in the first year, I took the flu vaccine. It gave me a reaction. I was sick for about three days. Like, I was fine and suddenly I got a fever, I felt bad and all that. I don't know if it's because my immunity dropped, or was already low, that this reaction occurred, or some component of it. I don't know. Because there's also the issue of allergies. Sometimes a person is allergic and doesn't even know it. For example, I'm allergic to Benzetacil. I can't take it. And before you take Benzetacil, you can, of course, get tested. Tests, right? Tests to see if you're allergic. If you'll have any reaction. Your body will react in some way. That component here, that's entering your body. So, only regarding that, regarding the benefit, it's great.

**Discourse:**  I also took the vaccine, like him, and it caused a reaction where I got the flu. I had nothing wrong with me. I took it and I got the flu. I had a fever, a runny nose. I took it and I got the flu. I took it so I wouldn't get the flu, and I got the flu. Now, I don't know if, according to the research, if suddenly you have flu symptoms, but that's how it is, that's not how it is. Because I was fine, I got vaccinated and then I got the flu.

**Discourse:**. Then, sometimes, resistance comes. Resistance.Moderator. That's a question you also have, right? From what you're saying. You'd like to know what the reaction would be if you took a new vaccine, specific to that vaccine. For example, what you're telling me is that you took the flu vaccine, you took my flu vaccine, and you had some reactions. So, you'd like to know what the mechanism is in your body when you take a vaccine. Is that it? What you said?

**Discourse:** Yes

**Discourse:** Yes

**Discourse:** In my case, I never had a reaction. Thank God.

**Discourse:** But there's no way to know if you don't get vaccinated. How will you know if the vaccine is safe if you haven't even had it yet, if it hasn't even been, how do you say, tested on the general population or even on prisoners? You're being a guinea pig, really.

**Discourse:** The creators, the people who had the time there, who studied for them, should give this explanation nationally.

**Discourse:** The government should govern the health area, join with the researchers who want to and give information.

**Discourse:** In school too, because when our children study, they get to the point.

**Discourse:** a specific person to explain about vaccines, about how it was studied, because for that there are people who are specialists to do studies, to benefit us, they would never send a vaccine to harm us. I'm sure of that, the Ministry of Health is there to take care of humanity, of everyone's health in general.

**Discourse:** To prevent health, right? Tuberculosis, I don't want to get tuberculosis, right?

**Discourse:** I don't know anything about tuberculosis. I'm learning now here in this conversation, right? Well, I don't know. Symptoms that I didn't really know about, I didn't know. And now I know. If I were more interested than just knowing what tuberculosis is, what the symptoms are, how to prevent it, if there will be a vaccine, right? I would take it. Without fear, right? Without fear.

**Discourse:** Yes. It's like arriving and saying, look, don't take this vaccine, because you will die. No, as she said, you will have... sequelae. You will become paralyzed. Right. You are passing on totally wrong information about what this is. The vaccine, right? So, just as it has good uses, there are people who use it for evil.

**Discourse:** I would be proud to know that the government is going to do this, is betting on this, the doctors, the researchers, to be guinea pigs. That's the situation we don't want, I wouldn't accept it then.

**Discourse:** I would be the first person to test it, to try it.

**Discourse:** I would only take this vaccine if it had been tested beforehand. If I took it, I would be thinking about how my body would react if I were to take it as a guinea pig, what the side effects would be, what its composition is, things I would be thinking about. But I would only take it after it was tested and approved by Anvisa (Brazilian Health Regulatory Agency) and the Public Prosecutor's Office.

**Discourse:** Because, people, before reaching humans, the vaccine they are going to test goes through laboratory testing on rats. That's usually how it is. Rats… Monkeys… First.

**Discourse:** The rat experiments. In animals. To see if it will have the effect of a rat dying. We can't just go to people and throw that vaccine that killed a rat at us.
