# Supplementary material for: Perceptions of persons deprived of liberty regarding tuberculosis vaccine research
Source: PLOS Glob Public Health. 2025 Dec 16;5(12):e0004941. doi: 10.1371/journal.pgph.0004941 (PMC12707645; doi:10.1371/journal.pgph.0004941)
Supplement: S1 Table — (DOCX) [file pgph.0004941.s002.docx]

**S1 Table.** Guiding Questions for Focus Group Discussions on Health Care, Tuberculosis, and Acceptability of a New TB Vaccine among Incarcerated Individuals

The S1 Table presents the semi-structured guiding questions used during the focus group discussions conducted with incarcerated individuals. These questions were designed to explore participants’ perceptions, experiences, and attitudes toward prison health care, tuberculosis, and the potential acceptability of a new TB vaccine. The interview guide aimed to elicit in-depth reflections on trust in health services, previous experiences with vaccination, perceived safety and effectiveness of vaccines, and ethical considerations related to participation in clinical research within prison settings. This framework ensured consistency across study sites while allowing flexibility for contextual adaptation and emergent themes during the discussions.

| **No.** | **Guiding Question** |
| --- | --- |
| 1 | How do you evaluate the health care you receive from the prison health team? |
| 2 | What is your experience with TB so far? |
| 3 | What information did you receive about TB in PU? |
| 4 | What do you think about vaccines and how acceptable are vaccines in general to you? |
| 5 | What is your experience with vaccines (inside and outside PU)? |
| 6 | What is the effect of vaccines on the body? Do you consider vaccines safe? |
| 7 | Do you trust healthcare professionals who administer vaccines? |
| 8 | What do you trust most for information about vaccines? |
| 9 | Have healthcare professionals always provided sufficient information about vaccines? |
| 10 | If a new vaccine against TB were introduced, would you accept taking it? |
| 11 | What are your concerns or fears regarding the use of the new TB vaccine? |
| 12 | What would you like to know about the new vaccine, and what information could influence your decision to take the vaccine or not? |
| 13 | Do you think the new vaccine being offered against TB will be effective? What benefits would it bring? |
| 14 | Do you think that a new vaccine against TB should be studied in prisons, since so many people in prison suffer from the disease? Or would it be better to test outside the prison, and only then bring the vaccines that have good results in studies to the prison population? Why? |
| 15 | Would you like to be invited to participate in a study of a new TB vaccine? And their families? |
| 16 | If you were invited to participate in a study of a new TB vaccine, what would you need to know to make a decision about whether or not to participate in the study? |
| 17 | Do you think all prisoners will accept the vaccine? |
| 18 | If you were thinking about participating in a study of a new TB vaccine, what concerns or fears would you have? |
| 19 | Do you feel pressured or coerced to participate in the study? Could the administration force you to participate in the study? Are there other reasons why you may not feel completely free to decide whether or not to participate? If you feel pressured or coerced, is there anything that can be done to facilitate a free decision for the person deprived of liberty? |
| 20 | There are people who think that those who are deprived of their liberty are not able to make a free choice about whether or not to participate in a study, and that therefore they should not be invited to participate. Others believe that those who are deprived of their liberty should not also be deprived of the opportunity to participate in studies, if they wish. What do you think? |
| 21 | Do you have any final thoughts on what we discussed? |
