# Supplementary material for: Perceptions of persons deprived of liberty regarding tuberculosis vaccine research
Source: PLOS Glob Public Health. 2025 Dec 16;5(12):e0004941. doi: 10.1371/journal.pgph.0004941 (PMC12707645; doi:10.1371/journal.pgph.0004941)
Supplement: S2 Table — (DOCX) [file pgph.0004941.s003.docx]

### **S2 Table: Distribution of Narratives by Core Theme, Subcategory, State, and Prison Type**

S2 Table provides the distribution of the narratives according to the defined core themes, subcategories, correctional facility unit, and the sex of the narrator. The table utilizes a colorimetric scale, presenting the absolute frequency of narratives. Specifically, shades of red are employed, where deeper tones indicate higher frequencies of narratives for a given category/subcategory combination, allowing for a quick visual identification of the most discussed or prevalent themes within specific prison settings and between male and female discourses.

###

| **Category** | **Subcategory** | **Male Prisons** | | | | | **Female Prisons** | | **Total Narratives (Male Prisons** | **Total Narratives (Female Prisons)** |
| --- | --- | --- | --- | --- | --- | --- | --- | --- | --- | --- |
|  |  | **State RS** | **State AM** | **State RO** | **State MG** | **State MS** | **State AM** | **State MS** |  |  |
| **Prison Health Services** | **Access to health services** | 6 | 8 | 6 | 4 | 5 | 0 | 1 | 29 | 1 |
|  | **Distrust in the prison system** | 0 | 0 | 3 | 3 | 2 | 1 | 2 | 8 | 3 |
|  | **Equity in health services** | 2 | 2 | 3 | 4 | 2 | 0 | 1 | 13 | 1 |
|  | **Neglect of rights** | 0 | 4 | 7 | 6 | 7 | 1 | 7 | 24 | 8 |
|  | **Neglect/deficiency of health care** | 0 | 3 | 4 | 4 | 3 | 6 | 21 | 14 | 27 |
| **Experiences with TB** | **Experiences with TB** | 25 | 12 | 11 | 7 | 7 | 16 | 9 | 62 | 25 |
|  | **Information received about TB in the Prison** | 4 | 2 | 4 | 2 | 2 | 3 | 0 | 14 | 3 |
|  | **Fear of contracting TB** | 2 | 1 | 6 | 3 | 6 | 0 | 1 | 18 | 1 |
|  | **Fear of passing on TB for family members during a visit** | 0 | 1 | 0 | 2 | 0 | 0 | 0 | 3 | 0 |
|  | **Disease risk (for self and cellmates)** | 1 | 3 | 4 | 4 | 7 | 1 | 0 | 19 | 1 |
| **New TB vaccines** | **Doubts about the creation of the vaccine** | 6 | 3 | 5 | 7 | 4 | 4 | 0 | 25 | 4 |
|  | **Insecurity about the introduction of protocol changes** | 3 | 0 | 1 | 3 | 2 | 0 | 0 | 9 | 0 |
|  | **Increased knowledge and reliable sources of information** | 6 | 3 | 4 | 3 | 3 | 3 | 0 | 19 | 3 |
|  | **Fear of not receiving post-vaccination care** | 1 | 1 | 0 | 2 | 2 | 2 | 3 | 6 | 5 |
|  | **Potential side effects of the new vaccine** | 1 | 2 | 1 | 5 | 2 | 0 | 1 | 11 | 1 |
|  | Satisfaction in fighting TB | 2 | 2 | 5 | 2 | 4 | 4 | 3 | 15 | 7 |
| **Clinical trials for a new tb vaccine** | **Trust in Science, Professionals, and Healthcare Institutions** | 2 | 0 | 2 | 10 | 7 | 7 | 1 | 21 | 8 |
|  | **Fear of Experimentation** | 12 | 1 | 2 | 9 | 6 | 4 | 2 | 30 | 6 |
|  | **Motivation (or Hesitancy) and Intention to Get Vaccinated** | 14 | 3 | 9 | 9 | 9 | 0 | 0 | 44 | 0 |
| **Vaccines In General** | **Confidence in vaccine administration by healthcare professionals** | 2 | 6 | 2 | 0 | 3 | 0 | 0 | 13 | 0 |
|  | **Confidence in vaccines (benefits and safety)** | 1 | 3 | 2 | 4 | 3 | 8 | 1 | 13 | 9 |
|  | **Prior knowledge about the vaccine** | 4 | 6 | 6 | 4 | 2 | 2 | 1 | 22 | 3 |
|  | **Belief in natural immunity** | 0 | 0 | 1 | 3 | 0 | 0 | 0 | 4 | 0 |
|  | **Personal experiences** | 9 | 5 | 10 | 6 | 3 | 0 | 1 | 33 | 1 |
|  | **Lack of information** | 3 | 9 | 5 | 7 | 5 | 5 | 1 | 29 | 6 |
|  | **Fear of side effects/risk of vaccines** | 1 | 4 | 2 | 0 | 4 | 3 | 0 | 11 | 3 |
|  | **Protection of individual and collective health** | 0 | 1 | 2 | 2 | 6 | 5 | 0 | 11 | 5 |
| **PDL autonomy** | **Individual autonomy constructed within the group context** | 1 | 4 | 3 | 1 | 2 | 9 | 0 | 11 | 9 |
|  | **Coercion** | 5 | 4 | 5 | 0 | 2 | 0 | 3 | 16 | 3 |
|  | **Group identity** | 1 | 1 | 4 | 1 | 1 | 0 | 2 | 8 | 2 |
|  | **Freedom of choice** | 9 | 4 | 12 | 6 | 7 | 12 | 1 | 38 | 13 |
|  | **Repression by staff and external circumstances** | 1 | 1 | 2 | 0 | 0 | 0 | 1 | 4 | 1 |
|  | **Fear of rights violations** | 1 | 0 | 2 | 2 | 1 | 0 | 0 | 6 | 0 |
