# Supplementary material for: Perceptions of persons deprived of liberty regarding tuberculosis vaccine research
Source: PLOS Glob Public Health. 2025 Dec 16;5(12):e0004941. doi: 10.1371/journal.pgph.0004941 (PMC12707645; doi:10.1371/journal.pgph.0004941)
Supplement: S3 Table — (DOCX) [file pgph.0004941.s004.docx]

### **S3 Table: Matrix of Narrative Summary: Convergence and Divergence by Core Theme**

S3 Table was constructed as a summary matrix to synthesize the findings from the analysis. This table presents a comprehensive overview of the **convergence** (areas of agreement or shared experience) and **divergence** (areas of difference or conflict) found across all narratives within each core theme.

To illustrate these findings, the matrix includes a sample of narratives for each theme, serving as concrete examples of the synthesized convergences and divergences. This table is further structured to present the findings separately for **male** and **female** discourses, facilitating a comparative analysis of the experiences and perceptions based on gender within the correctional system.

.

|  | | | | |
| --- | --- | --- | --- | --- |
| **Dimension and Categories** | **Data Synthesis (Male)** | **Data Synthesis (Female)** | **Illustrative Discourses (Male)** | **Illustrative Discourses (Female)** |
| Perceptions of health in the prison (prison health services) | **Convergence:** Participants across different prison units recognized the presence of a structured tuberculosis (TB) care system, including medication delivery, routine testing, and isolation practices. Many acknowledged that health professionals provide treatment, follow-up, and monitoring to prevent outbreaks. There is shared understanding that TB is a serious but controllable disease within the prison environment when treatment protocols are followed.  **Divergence:** While some participants described health services as organized, efficient, and “excellent,” others reported inconsistent care, medication delays, inadequate isolation conditions, and lack of information about vaccines or TB procedures. Perceptions varied widely by facility—ranging from strong satisfaction with medical attention to frustration over neglect, lack of medicines, and delays in diagnosis or referrals. These contrasting accounts reveal disparities in access, quality, and timeliness of prison health care across sites. | **Convergence:** PDLs statements converge on perceptions of a slow and inefficient health care process, where individuals must “beg” for attention from nurses. The lack of communication during the pandemic generated extreme anxiety, with fears that staff might “lock the doors and let us die.”  **Divergence:** Divergence emerges regarding isolation logistics and vaccine perceptions. While some described delayed and ineffective isolation—removing infected individuals only after the virus had spread through the cell—others expressed distrust toward vaccination, believing it to be a “chip.” Although the vaccination process was logistically organized (in groups of three), delays and lack of follow-up led to doubts about the effectiveness of care. | **Discourse:** "I have been a health worker here for twelve years... And the treatment was always very adequate, everything."  **Discourse:** "Health here is excellent. It always has been, you see? Everyone well attended to. Hospitalizations, everything. Everything has always been referred."  **Discourse:** "The area of assistance here regarding health, regarding the TB issue, has extreme control, right? No... There was never a case of someone dying from TB here."  **Discourse:** "Everyone receives their medicine, the exams are all done directly. Anyone who has a health problem that we notice is already referred to where they already do the exam and start treatment. So as not to allow that time to accumulate, right?"  **Discourse:** "the inmate, the detainee is the person who knows best how to manage... Because the first aid comes from us... But there are situations that are not resolved. It needs deeper care, greater attention." | "**Discourse 1:** "People don't have time to bring the vaccine, they ask who wants to get a vaccine, it's an invitation, they just ask who wants it, then they leave, open the door, leave in groups of three, in three. Once a girl said, no, what if it’s the chip."  **Discourse:** "When the virus was there, did we think we were going to die? It’s over, the world is ending and we are here inside and they don't give information, we thought they would lock the door and let us die of the virus here."  **Discourse:** "For example, when I caught the virus after a long time I was really bad, I already had no taste, no smell, fever, fever on top of fever I found a lot, lot, lot of lodging..."  **Discourse:** "Sometimes we say this. Ma'am, please, you Ma'am, here with the others. Please, she is feeling sick.  **Discourse: "**The only information we have is the television... there is no information at all. They just come and say, it’s the flu shot so take it. That's it." |
|  |  |  |  |  |
|  |  |  |  |  |
|  |  |  |  |  |
|  |  |  |  |  |
|  |  |  |  |  |
|  |  |  |  |  |
| Perceptions of health in the prison(experiences with TB) | **Convergence:**The convergence is clear in identifying the prison environment as extremely high-risk for TB transmission due to overcrowding and the absence of adequate isolation. There is consensus on the essential role of internal “health agents” in providing first aid and triage, compensating for gaps in the official health system.  **Divergence:** The main divergence lies in perceptions of TB treatment quality. While participants from one unit described care as “adequate” and “excellent,” others reported negative experiences, characterizing treatment as “not worthy” and recalling unsafe situations in which they were housed alongside individuals with active | **Convergence:** The female discourse converges on the denunciation of systemic failure in tuberculosis care. Participants report that the disease is often downplayed as “a simple flu,” and once treatment begins, it is marked by precarious conditions, including medication interruptions lasting up to two weeks. This institutional neglect contrasts with individual efforts to seek care, such as an inmate who independently went to a health post. Another strong convergence concerns the “prejudice” and stigma associated with infection, leading to social isolation.  **Divergence:** The divergence lies in how fear and stigma are interpreted—participants emphasize that such reactions are not limited to tuberculosis but extend to a broader fear of “several infectious diseases.” | **Discourse:** "I work as a health agent here for two years and six months and already within the cell block six cases of tuberculosis have been treated... In relation to the treatment on the health side, the infirmary with them, it has always been adequate."  **Discourse:** "Actually, correcting, this week a guy came to live with us in the same cell who had tuberculosis. He had already been undergoing treatment on the street for four months and it was necessary to collect him again. So, he is there continuing the treatment."  **Discourse:** "The prisoner, the inmate is the person who knows how to cope the most. Because I am learning, in prison, something tremendous. Because the first aid comes from us. I am a life donor, I bring the first aid."  **Discourse:** "I see that any initiative to care for the inmate, who is very needy, is welcome, but a research work, like you are doing, with this initiative, in my view, will give us much more security and peace of mind to accept."  **Discourse:** "This week a guy came to live with us in the same cell who had tuberculosis. He had already been under treatment for four months outside and he needed to be collected again." | "**Discourse:** "I lived with Dona Leda too and the prejudice was very evident, because people stayed away from her, far from her bed. And my bed was above hers and I was one of the few people who talked to her in the unit, and she was already an elderly woman, she wore a mask all the time. And the prejudice was extreme..."  **Discourse:** "I lived with four girls who had tuberculosis. The first one who had tuberculosis, we called security for her, and people said it was a simple flu and the girl went out and went to the health clinic and confirmed that she had tuberculosis."  **Discourse:** "The other one here inside, our colleague, she went two weeks without medication. When medication starts, it's every day before food... and also two weeks without taking medication, the four girls I lived with had this problem, of stopping the medication."  **Discourse:** "There are situations that are not resolved. It requires deeper attention, greater attention."  **Discourse:** "it's when you see the girl was going to take it at 10 or 11 in the morning. She had already eaten bread, she had already eaten everything." |
|  |  |  |  |  |
|  |  |  |  |  |
|  |  |  |  |  |
|  |  |  |  |  |
|  |  |  |  |  |
| Perceptions of vaccines (vaccines in general) | **Convergence:** Male participants generally converge on expressions of reluctance and skepticism toward vaccination, citing the high frequency of campaigns and lack of transparency about the types of vaccines administered. Many reported feeling overwhelmed by the number of doses and questioning the legitimacy of recent immunizations because they were unaware of the vaccines’ specific names.  **Divergence:** The strongest divergence centers on the perceived coercion linked to vaccination. Acceptance often stems from fear of losing visitation rights (“everyone becomes reluctant, saying that if they don’t take it, they won’t get visits”) rather than voluntary trust in vaccine efficacy. Additionally, negative personal experiences (“I took it not to get the flu, I got the flu”) reinforce mistrust and skepticism about the vaccine’s validity and side effects. | **Convergence:** Female participants generally converge on a fundamental acceptance of vaccination as a public health measure, expressing trust in the idea that vaccines reduce symptom severity (“you catch it but your symptoms are less”) and a consistent desire for protection, regardless of the vaccine brand.  **Divergence:** The main divergence lies in the lack of transparent communication and the passive delivery of vaccines within the prison unit. Information is often limited to television rumors or brief, superficial messages from staff (“They just arrive and say, it’s the flu vaccine, so take it. That’s all.”). This lack of proper explanation creates a clear disconnect between vaccination initiatives and health education, ultimately limiting full confidence in the process. | D**iscourse:** "Very reluctant, people no longer know with so many vaccines. All the time vaccine, vaccine. Then everyone becomes reluctant, saying that if they don't take it, they won't get visits. Everyone gets vaccinated." **Discourse:** "I didn't take it. They block visitation... I've already taken six vaccines. I won't take any more."  **Discourse:** "I also took the vaccine, like him, and it gave me the reaction that I caught the flu. I had nothing. I took it and I got the flu. I took it not to get the flu, I got the flu. Now, I don't know if, according to the research, if you suddenly have flu symptoms, but that's how it is, isn't it? Because I was fine, I took it and got the flu."  **Discourse:** "The last vaccine, they couldn't even say the name. They just said it was the new, modern vaccine."  **Discourse:** "But a vaccine, I'll give you this vaccine, you don't catch it, but for tuberculosis I wouldn't take it." | **Discourse:** ""The only information we have is on television, so yes, sometimes we can't find accommodation because there's no accommodation, so there's no information at all. They just come and say, 'It's a flu vaccine, so take it.' That's all."  **Discourse:** "But the vaccine doesn't mean you won't catch it, you catch it but your symptoms are less."  **Discourse:** "I will share with you from the beginning of COVID-19, the rumor that it was going to be a pandemic... I already thought that way, I followed the newspaper... the agents are the ones who go out to the street, the virus will come through them. I don't want to, I want the vaccine to come. But it took a while."  **Discourse:** "So when the second, third, fourth dose arrives, I don't even want to know if it's AstraZeneca or another, I'm going to take it and me..."  **Discourse:** "Because, folks, before reaching humans, the vaccine they are going to test goes through a laboratory on rats... We cannot reach people and throw that vaccine that a rat died from at us." |
| Perceptions of vaccines (new TB vaccines) | **Convergence:** The convergence in the decision to accept or refuse vaccination is strongly linked to the availability of transparent information regarding vaccine development, safety, and efficacy. Persistent fears of being used as “test subjects” and of experiencing severe side effects serve as key deterrents. Coercion, particularly the threat of losing visitation rights, was cited as the main motivation for accepting other mandatory vaccines.  **Divergence:** Divergence emerges in expressions of appreciation toward researchers’ efforts to consult with them, which some participants interpreted as a positive gesture from the health system. A few also expressed willingness to accept potential risks if the vaccine offered clear and tangible personal health benefits. | **Convergent:** The primary convergence is a profound distrust of any experimental vaccine, reflected in participants’ insistence on approval by high-level external authorities such as ANVISA or the Public Ministry, and on rigorous preclinical testing in animals (rats/monkeys) before human use. Many fear being treated as “guinea pigs,” a perception intertwined with their self-image as the “trash of society” or those who “matter the least.”  **Divergent:** Divergence emerges in the identified sources of trust. While internal prison staff are broadly viewed as lacking credibility (“if it were from the house, we wouldn’t trust”), several participants expressed willingness to participate if the research were conducted by “outside people,” signaling that acceptance depends on assurances of ethical conduct, transparency, and external oversight. | **Discourse:** "I would accept the vaccine if we arrived there, with all the explanation, and they said, look... We are going to take the tuberculous people in the cell block, we are going to give them the vaccine, you will see that they will become immune... will you be immune forever? That's something I would accept."  **Discourse:** "You are being a guinea pig, actually, to test it, actually. It is the most direct way to talk about a vaccine... And the people are us. Only by taking it can we know its reaction."  **Discourse:** "We had to see the results of the exams, right? And you who produce the vaccine, you have to show it, right? Because the thing is to know the result out there. It has to have an approval, right? An approval, everything works, right?"’  **Discourse:** "I will not go out, because the ones who go out to the street are the agents, the virus will come through them. I don't want it, I want the vaccine to come. But it took a long time." | **Discourse:** "I would only take it after it was tested, tested, approved by Anvisa and the Public Prosecutor's Office." **Discourse:** "Because, people, before it reaches human beings, the vaccine they are going to test goes through a laboratory on rats... We cannot come with people and inject them with that vaccine that a rat died with."  **Discourse:** "We take it, but sometimes we think we are being guinea pigs."  **Discourse:** "Due to the way we are treated both inside the unit and outside, viewed, as if, roughly speaking, grotesquely, the trash of society, they are people who matter least." |
|  |  |  |  |  |
|  |  |  |  |  |
|  |  |  |  |  |
|  |  |  |  |  |
|  |  |  |  |  |
| Perceptions of participation in research (clinical trials for a new TB vaccine). | **Convergence:** The main convergence is a pervasive reluctance and mistrust toward vaccines, particularly the more recent ones. This skepticism is driven by the frequency of new doses, lack of transparency about vaccine composition, and a prevailing sense of coercion—especially the perceived threat of losing visitation rights for noncompliance. Reports of adverse side effects, such as acute chest pain, reinforce this distrust and deepen collective apprehension.  **Divergence:** The divergence lies in a small subset of participants who continue to support vaccination as a public health measure. Moreover, participants differentiate between traditional, well-established vaccines—which they generally accept—and newer, more frequent doses, which they perceive as being imposed without adequate explanation or consent. | **Convergence:** The female discourse on general vaccines, such as COVID-19, converges on a pragmatic acceptance of vaccination, motivated by intense fear of the pandemic and the belief that vaccines lessen symptom severity (“you get it but your symptoms are less”). This compliance occurs amid a lack of reliable information, as participants rely primarily on television, while internal communication is limited to brief directives (“just told to take the flu shot”).  **Divergence:** The main divergence lies in the underlying rationale for vaccine acceptance. For some, adherence stems from panic and the desperate desire to avoid death; for others, it reflects a more reasoned understanding of risk reduction and disease mitigation—revealing a tension between emotional fear and rational trust in vaccine functionality. | "**Discourse:** "I would accept the vaccine if we arrived there, with all the explanation, and they said, look... We are going to take the tuberculous people in the cell block, we are going to give them the vaccine, you will see that they will become immune... will you be immune forever? That's something I would accept."  **Discourse:** "If you take it, if you don't take it, you will sign the term of responsibility there, you may take it, you may not. When the topic of visitation comes up. Then, the inmate pulls the handbrake. So, I'll take it."  **Discourse:** "You are being a guinea pig, actually, to test it, actually. It is the most direct way to talk about a vaccine... And the people are us. Only by taking it can we know its reaction."  **Discourse:** "We had to see the results of the exams, right? And you who produce the vaccine, you have to show it, right? Because the thing is to know the result out there. It has to have an approval, right? An approval, everything works, right?" | "**Discourse:** "I would only take this vaccine if it had been tested afterwards. If I took it, I would be thinking about how my body would react if I were to take it as a guinea pig, how it works, what side effects it would have on me, what compositions it has, these things that I would be thinking about, but I would only take it after it was tested and approved by Anvisa and the Public Ministry."  **Discourse:** "Because, people, before it reaches human beings, the vaccine they are going to test goes through a laboratory on rats... We cannot come with people and inject them with that vaccine that a rat died with." **Discourse:** "We take it, but sometimes we think we are being guinea pigs."  **Discourse:** "due to the way we are treated both inside the unit and outside, viewed, like this, roughly speaking, the grotesque trash of society, we are the people who matter the least." |
|  |  |  |  |  |
|  |  |  |  |  |
|  |  |  |  |  |
|  |  |  |  |  |
| Perceptions of participation in research (PDL autonomy) | **Convergence;** The autonomy of the male PPL converges on proactivity in self-management of health, where the internal agent actively monitors TB symptoms and insists on tests, compensating for the lack of institutional resources. There is a consensual valorization of the "right to be heard" and of consent in research, viewed as a recovered respect. **Divergence:** However, the main divergence lies in institutional coercion, where vaccination is perceived as a forced imposition, nullifying free will, often under the threat of losing rights such as intimate visitation. Added to this is the fear of being "guinea pigs" or lab rats in unproven studies, with the explicit demand for institutional accountability in case of damages. | **Convergence:** Participants converge on the perception of being systematically blamed by health staff for outbreaks or health issues, as ca1ptured in the statement, “Every time they come with a subject, the fault is usually ours.” This sense of blame reinforces their lack of autonomy, with the immediate suspension of visitation or restriction on receiving materials often cited as the first institutional response during health crises.  **Divergence:** The divergence lies in how participants identify the true source of infection. Although they feel blamed, PDL consistently point to prison staff and agents as the actual vectors introducing the virus from outside, undermining the effectiveness of isolation measures. They also express a strong demand for improved communication, specifically requesting that information be shared “cell by cell” to guarantee fair and equal access to health updates. | **Discourse:** ‘’I wanted to say that the right to be heard is very good for us. When everyone enters this system, we lose a lot of rights. This right to be heard is very good for us. So, as long as there's respect on both sides, it's easier for us to accept the treatment itself. So, everyone will accept it. I believe it's more than 80%. More than 80%. Most people want to learn about healthcare. Everyone wants to go home. That's the truth. Respect is very good.’’  **Discourse:** ‘’So, if I could participate in this, it would be for us to feel better, not only would we have more knowledge, but we would pass it on to future generations.’’  **Discourse**: ‘’ I already think everyone felt comfortable here, because at the beginning of the conversation, it started on the right side, it started by asking who wanted it and who didn't, and what it is, who is it. And it gave us the chance to say, "Look, do we want to stay or not?" I think that's it. Now, the part I think is right: everyone listens whether they want to or not. I think the conversation always starts off right when one person asks if the other wants to hear you or not. I think that's it.’’  **Discourse:** “I think everyone felt comfortable here, because from the beginning of the conversation it started the right way, they [the focus group facilitators] started by asking who wanted and didn’t want to stay. And they gave us the chance, do we want to stay or not?  **Discourse:** ‘’I personally wouldn't participate in two ways. The first is because, as you said, it's something that's being tested, something that doesn't exist yet, is still being formulated. So, essentially, what would we be? Guinea pigs. And since we're signing this paper now, here, taking full responsibility, we won't gain anything. We might, down the road, perhaps get one less day of remission, you know? If a side effect were to happen to any of us during these tests, we would be harmed ourselves, and that would be it. But if you provided the explanation, the paper here, which we'll be signing and reading, and it stated that you were taking responsibility for any potential seriousness or error that might occur to us, then yes, I would participate.’’  **Discourse:** ‘’I would accept to be part of this research, especially because the vaccine is something very important for our human society. We know that since the last century, there have been many diseases whose mortality has been prevented precisely because of the vaccine, which provides us with our defenses. I believe so, because with new research, and even more so on TB, which is very important in our society, regarding our deprivation of liberty and our coexistence in groups, then it would be very important and essential. We know that yes, there could not be just one vaccine, but rather there could be other types of vaccines. Because as time goes by, we know that all viruses and bacteria only have mutations and are constantly changing. I am sure that there would be not only one vaccine, but constant changes within vaccines. With this research bringing adequate information for us to understand what is happening, I believe that we would agree to take it, and it is essential for us humans to avoid both mortality and the exploitation of viruses for maximum contamination.’’ | **Discourse:** "I personally wouldn't participate in two ways. First, I wouldn't participate because, as you said, it's something that's being tested, something that doesn't exist yet, is still being formulated. So, practically, what would we be? Guinea pigs. And, since we signed this paper now, here, fully taking responsibility, we won't gain anything. Maybe, down the road, we could get one less day of remission, you know? If a side effect were to happen to any of us in these tests, we would be harmed ourselves, and that would be it." But, if you provided the explanation, the paper here, which we will be signing and reading, and it stated that you would be responsible for any potential seriousness or error that might occur to us, then yes, I would participate.  **Discourse**: "I would accept being part of this research, especially because the vaccine is very important for our human society. We know that since the last century, many diseases have been prevented, mortality rates precisely because of the vaccine, which provides us with our defenses. I believe so, because with new research, especially on TB, which is very important in our society, regarding our deprivation of liberty and our group coexistence, it would be very important and essential. We know that there could not be just one vaccine, but rather other types of vaccines. As time goes by, we know that all viruses and bacteria only mutate and are constantly changing. I am sure that there would be not just one vaccine, but constant changes within vaccines. With this research providing adequate information for us to understand what is happening, I believe we would agree to take it." And it's essential for us humans to avoid both mortality and the exploitation of viruses that can cause maximum contamination.  **Discourse:** "Yes, I would like to, yes, accept being part of research like this because I know it wouldn't just involve me, but we are part of the research because we are part of the research. I wouldn't just involve myself, but also the multitude of other people who need treatment. So I would be happy if I would willingly do it for myself alone, but for everyone in general, so yes, I would accept it."  Discourse: "I wouldn't change my mind. However, it's something that... It's something that affects us, it's our family, our children, our mother and father. I've already lost a mother, I've already lost a father. So, I would never accept losing my children for anything in the world. Just their opinion. Or in their case, yes, I would take 50,000 vaccines for them. Other than that, nothing else, I wouldn't change my mind, no way." |
