# Supplementary material for: Perceptions of persons deprived of liberty regarding tuberculosis vaccine research
Source: PLOS Glob Public Health. 2025 Dec 16;5(12):e0004941. doi: 10.1371/journal.pgph.0004941 (PMC12707645; doi:10.1371/journal.pgph.0004941)
Supplement: Attachment — Submitted filename: MM. Critical Assessment of the Article.pdf [file pgph.0004941.s005.pdf]

Critical Assessment of the Article

Perceptions of persons deprived of liberty regarding tuberculosis vaccine research

Strengths

- Novel and highly relevant topic (perceptions of persons deprived of liberty—PDL—regarding TB vaccine trials).
- Well-described qualitative methodology (focus groups, inclusion criteria, thematic analysis with triangulation).
- Inclusion of 91 participants across 7 prisons in 5 Brazilian states, providing diversity.
- Strong discussion, linking findings to international ethical frameworks and recent literature on vulnerability and the right to science.
- Practical recommendations (6 points) are clear and directly derived from participants' testimonies.

Weaknesses / Areas for Improvement

- **Sampling limitations:** Convenience sampling was used. The discussion acknowledges this, but could expand on how it affects transferability of results.
- **Representation of female voices:** Women were included (30%), but most quotations highlighted are from men. Balancing quotes would strengthen the narrative.
- **Confusion in one passage:** (e.g., "It's mandatory to take it…"—unclear whether referring to COVID-19 vaccination or TB trial participation). This needs clarification.
- **Quotations:** Some participant quotes are long and repetitive; condensing would improve readability.
- **References:** Correct incomplete entries (Ako 2020, Furin 2019, etc.) and standardize formatting.

1. Does this manuscript meet PLOS Global Public Health's publication criteria? Is the manuscript technically sound, and do the data support the conclusions?

Yes. The manuscript is technically sound and methodologically rigorous within the qualitative research paradigm. The use of focus groups across multiple prisons, with systematic thematic content analysis, is appropriate for the research question. Ethical approvals are clearly documented. The conclusions are well aligned with and supported by the data presented.

2. Has the statistical analysis been performed appropriately and rigorously?

Yes. This is a qualitative study. Quantitative analysis is limited to descriptive statistics (counts, percentages, mean age) to contextualize the sample, which is appropriate. The qualitative analysis was conducted rigorously, including verbatim transcription, double coding, thematic categorization, and triangulation. No advanced inferential statistics were required or expected for the study design.

3. Have the authors made all data underlying the findings in their manuscript fully available?

Partially. The Data Availability Statement indicates that data are available upon reasonable request from the corresponding author, due to ethical and confidentiality concerns involving incarcerated participants. While this justification is valid, it does not fully comply with PLOS's policy requiring unrestricted availability. The authors may wish to strengthen compliance by providing anonymized coded data, excerpts, or aggregated datasets that do not compromise participant confidentiality.

4. Is the manuscript presented in an intelligible fashion and written in standard English?

Yes. The manuscript is clearly written, logically structured, and presented in professional English. Participant quotations are used effectively, though some could be shortened for readability. Minor stylistic editing (condensing lengthy sentences, polishing phrasing in the Discussion) would improve clarity, but overall the manuscript is intelligible and suitable for publication.

5. Review Comments to the Author

This is a timely and important manuscript addressing the ethical and practical considerations of including persons deprived of liberty in tuberculosis vaccine trials. The study is methodologically sound, and the findings provide valuable insights into participants' perceptions and concerns, with clear implications for public health policy and research ethics.

Strengths:

- Novel and relevant topic, addressing a major gap in TB research.
- Strong qualitative methodology with triangulation and multi-site sampling.
- Ethical approvals clearly described.
- Well-supported conclusions and practical recommendations grounded in participant voices.

Points for improvement:

1. **Data availability:** While confidentiality concerns are valid, consider providing an anonymized coded dataset or expanded supplementary material (e.g., summary matrices) to strengthen compliance with PLOS's data policy.

2. **Quotations:** Some direct quotations are lengthy or repetitive; consider condensing for readability without losing participants' perspectives.
3. **References:** A few references are incomplete (e.g., Ako 2020; Furin, Cox, Pai 2019) and should be corrected/standardized.
4. **Clarity in Results:** In one passage, the phrase *"It's mandatory to take it"* is ambiguous (COVID-19 vaccine vs. TB trial participation). Clarify context.

Overall, this is a strong and ethically rigorous manuscript that will make an important contribution to the field. With minor revisions, it is suitable for publication.

6. Confidential to Editor

I recommend acceptance after minor revisions, primarily concerning data availability compliance, reference corrections, and minor stylistic improvements.

- **Identity disclosure:** I prefer to remain anonymous for this review.
- **Assistance:** No assistance was received in preparing this review.
- **Highlighting:** Yes, I recommend this article be highlighted. It addresses a neglected but highly important issue at the intersection of TB control, incarceration, and research ethics, with global relevance.
