# Supplementary material for: Perceptions of persons deprived of liberty regarding tuberculosis vaccine research
Source: PLOS Glob Public Health. 2025 Dec 16;5(12):e0004941. doi: 10.1371/journal.pgph.0004941 (PMC12707645; doi:10.1371/journal.pgph.0004941)
Supplement: Attachment — Submitted filename: Rebuttal letter.docx [file pgph.0004941.s007.docx]

**Response to Reviewers**

*Manuscript Title:* ***Perceptions of persons deprived of liberty regarding tuberculosis vaccine research.***

*Manuscript ID: PGPH-D-25-01609*

*Journal: PLOS Global Public Health*

Dear Dr. Zulma Vanessa Rueda,

We would like to thank you and the reviewers for your thoughtful and constructive comments on our manuscript. We appreciate the time and effort dedicated to providing feedback, which has greatly helped us improve the clarity, rigor, and overall contribution of our work.

We have carefully addressed all the editorial and reviewer comments, revised the manuscript accordingly, and provide detailed responses below. All changes in the manuscript are highlighted in the file titled “***Revised Manuscript with Track Changes and Revised Manuscrupt****”.*

**Editorial Requirements**

1. ***Please amend your detailed Financial Disclosure statement. This is published with the article. It must therefore be completed in full sentences and contain the exact wording you wish to be published.***

*1. Please clarify all sources of funding (financial or material support) for your study. List the grants (with grant number) or organizations (with url) that supported your study, including funding received from your institution.*

*2. State the initials, alongside each funding source, of each author to receive each grant.*

*3. State what role the funders took in the study. If the funders had no role in your study, please state: “The funders had no role in study design, data collection and analysis, decision to publish, or preparation of the manuscript.”*

*4. If any authors received a salary from any of your funders, please state which authors and which funders.*

*If you did not receive any funding for this study, please simply state: “The authors received no specific funding for this work.”*

Response:

We revised the Financial Disclosure statement to provide full sentences, specify funding sources and grant numbers, indicate the authors associated with each funding source, and clarify the role of funders. The updated statement now reads:

*“This study was supported by* ***Open Philanthropy****. The funders had no role in study design, data collection and analysis, decision to publish, or preparation of the manuscript.”* ***Lines: 722-723.***

2. **In the online submission form, you indicated that The data that support the findings of this study are available from the corresponding author upon reasonable request, in accordance with ethical guidelines and participant confidentiality.**

All PLOS journals now require all data underlying the findings described in their manuscript to be freely available to other researchers, either

1. In a public repository,

2. Within the manuscript itself, or

3. Uploaded as supplementary information.

This policy applies to all data except where public deposition would breach compliance with the protocol approved by your research ethics board. If your data cannot be made publicly available for ethical or legal reasons (e.g., public availability would compromise patient privacy), please explain your reasons by return email and your exemption request will be escalated to the editor for approval. Your exemption request will be handled independently and will not hold up the peer review process, but will need to be resolved should your manuscript be accepted for publication. One of the Editorial team will then be in touch if there are any issues.

Response:

We revised the Data Availability Statement to comply with PLOS data policy. We have uploaded an anonymized coded dataset (summary matrices) as supplementary material (S1 Dataset), which preserves participant confidentiality while providing transparency and accessibility for other researchers.

**3. Please provide separate figure files in .tif or .eps format.**

**For more information about figure files please see our guidelines:** 
[**https://journals.plos.org/globalpublichealth/s/figures**](https://journals.plos.org/globalpublichealth/s/figures) 
[**https://journals.plos.org/globalpublichealth/s/figures#loc-file-requirements**](https://journals.plos.org/globalpublichealth/s/figures#loc-file-requirements)

Response:

All figure files have been uploaded separately in .tif format according to journal guidelines.

**4. 4. Please insert an Ethics Statement at the beginning of your Methods section, under a subheading 'Ethics Statement'. It must include:**

1) The name(s) of the Institutional Review Board(s) or Ethics Committee(s)

2) The approval number(s), or a statement that approval was granted by the named board(s)

3) (for human participants/donors) - A statement that formal consent was obtained (must state whether verbal/written) OR the reason consent was not obtained (e.g. anonymity). NOTE: If child participants, the statement must declare that formal consent was obtained from the parent/guardian.

Response:

We inserted an Ethics Statement at the beginning of the Methods section. The statement includes the name of the ethics committee, approval number, and details on written informed consent obtained from all participants **Location:** Section **Materials and Methods**, line 123 a 134.

**5. We do not publish any copyright or trademark symbols that usually accompany proprietary names, eg (R), (C), or TM  (e.g. next to drug or reagent names). Please remove all instances of trademark/copyright symbols throughout the text, including ® on page 7, 8.**

Response:

We removed all ®, ©, and ™ symbols throughout the text, including pages 7 and 8.

Questionnaire data were stored in a secure **REDCap** and MaxQDA24(29) software was used to organize and highlight the coded texts (line 206 and 220).

**6. Some material included in your submission may be copyrighted. According to PLOS’s copyright policy, authors who use figures or other material (e.g., graphics, clipart, maps) from another author or copyright holder must demonstrate or obtain permission to publish this material under the Creative Commons Attribution 4.0 International (CC BY 4.0) License used by PLOS journals. Please closely review the details of PLOS’s copyright requirements here: PLOS Licenses and Copyright. If you need to request permissions from a copyright holder, you may use PLOS's Copyright Content Permission form.**

Response:

The geographic distribution map of study recruitment sites across Brazil was created using the base shapefile of Brazil’s administrative boundaries obtained from the Brazilian Institute of Geography and Statistics (IBGE). The shapefile is publicly available at: <https://www.ibge.gov.br/geociencias/organizacao-do-territorio/malhas-territoriais/15774-malhas.html>.
Use of the IBGE cartographic base complies with the CC BY 4.0 license and the institution’s open data terms of use. No copyrighted or proprietary material has been used in this figure.

**7. We notice that your supplementary tables are included in the manuscript file. Please remove them and upload them with the file type 'Supporting Information'. Please ensure that each Supporting Information file has a legend listed in the manuscript after the references list.**

Response:

We removed all supplementary tables from the main manuscript and uploaded them as separate Supporting Information files (S1–S3 Tables), each with appropriate legends listed after the References.

### **Table S1: Distribution of Narratives by Core Theme, Subcategory, State, and Prison Type**

### **Table S2: Matrix of Narrative Summary: Convergence and Divergence by Core Theme**

**Table S3:** **Guiding Questions for Focus Group Discussions on Health Care, Tuberculosis, and Acceptability of a New TB Vaccine among Incarcerated Individuals**

**8. If the reviewer comments include a recommendation to cite specific previously published works, please review and evaluate these publications to determine whether they are relevant and should be cited. There is no requirement to cite these works unless the editor has indicated otherwise.**

Response:

We reviewed and updated all references in the manuscript to ensure accuracy and consistency. Missing entries (e.g., Ako 2020; Furin, Cox, and Pai 2019) were completed, and formatting was standardized according to PLOS style guidelines. We also incorporated additional citations suggested by Reviewer #1 and relevant to our study context. No retracted or unreliable sources have been cited.

Specifically, we added five new references to strengthen the discussion:

**36.** Legrand NM, Bull RA, Hajarizadeh B, Lloyd AR, Johnston K, Issa K, et al. Surveillance of Viral Respiratory Infections within Maximum-Security Prison, Australia. Emerg Infect Dis. 2025;31. doi:[10.3201/eid3108.240571](https://doi.org/10.3201/eid3108.240571)2.

**37.** Talevski J, Wong Shee A, Rasmussen B, Kemp G, Beauchamp A. *Teach-back: A systematic review of implementation and impacts.* Mathes T, editor. *PLoS ONE.* 2020;15:e0231350. doi:10.1371/journal.pone.0231350

**38.** Andrews JR, Charalambous S, Churchyard G, Cobelens F, Fernández-Escobar C, Frick M, et al. *The participation of people deprived of liberty in tuberculosis vaccine trials: should they be protected from research, or through research?* *The Lancet Infectious Diseases.* 2025; S1473-3099(25)00305-6. doi:10.1016/S1473-3099(25)00305-6

**39.** Moazen B, Ismail N, Agbaria N, Mazzilli S, Petri D, Amaya A, D'Arcy J, Plugge E, Tavoschi L, Stöver H. *Vaccination against emerging and reemerging infectious diseases in places of detention: a global multistage scoping review.* *Front Public Health.* 2024 Jan 29;12:1323195. doi:10.3389/fpubh.2024.1323195

**40.** Chin ET, Leidner D, Zhang Y, Long E, Prince L, Li Y, et al. *Effectiveness of the mRNA-1273 Vaccine during a SARS-CoV-2 Delta Outbreak in a Prison.* *New England Journal of Medicine.* 2021;385(24):2300-2301. doi:10.1056/NEJMc2114089

**Responses to Reviewer #1**

We are grateful for your insightful and thorough review. We have revised the manuscript substantially to address all your comments. Below we provide a point-by-point response.

**Minor comments**

**Question 1: Under "Respect for autonomy," the bracketed note "[what is 'it' referring to? ...]" should be removed from the final publication version as it appears to be an editorial artifact.**

Response:

We removed the bracketed note “[what is 'it' referring to? ...]” as suggested.

“It’s mandatory to take it [Must get vaccinated against COVID-19]. Even if someone refuses, if they insist on not taking it, they might lose, lose their visitation rights — as we’ve already been threatened with losing visits. [...] That’s why it becomes an obligation”. (**Male)**

Section Table 2: Perceptions of participants regarding health care in prisons, TB, vaccines, and participation in research, line 251 a 253.

**Question 2. The discussion could be strengthened by linking participants’ expressed willingness to vaccination with empirical evidence that incarcerated populations both accept and benefit from vaccination. For example, this recent paper in a maximum-security prison found that SARS-CoV-2 vaccination conferred substantial protection mid-outbreak, despite overcrowding and multiple viral introductions (DOI: 10.3201/eid3108.240571). Citing evidence from other real-world scenarios and infections would reinforce the finding that PDL are not inherently resistant to vaccination and that vaccination remains effective, even under extreme transmission conditions. This would underscore the relevance and public health good of including PDL in TB (and other) vaccine trials.**

Response:

We revised the Discussion section to incorporate empirical evidence on the effectiveness and acceptance of vaccination in prison settings. We cited the suggested paper Legrand et al., 2025 (Ref. 36) (DOI: 10.3201/eid3108.240571), as well as additional relevant studies—Moazen et al., 2024 (Ref. 39) (doi: [10.3389/fpubh.2024.1323195](https://doi.org/10.3389/fpubh.2024.1323195) ) and Chin et al., 2021 (Ref. 40) ( . doi: 10.1056/NEJMc2114089). These quotations are found in lines 483 to 491.

**Question 2. The recommendations, in their current form are a bit too abstract to serve as a practical guide, and read as a conclusion rather than a tool for action. The authors are uniquely positioned to leverage their empirical findings to propose concrete and actionable operational frameworks. I urge them to revise this section to provide the specific, nitty-gritty details that would transform these principles from admirable goals into a usable blueprint for ethical practice. e.g. The advice to "tailor" consent is generic. What does a prison-specific consent process concretely look like in terms of literacy level, cultural format, and verification checkpoints to ensure it is a truly ongoing process?**

Response:

We have significantly restructured the Recommendations section to provide concrete, actionable operational guidance. Beyond specifying elements of a prison-specific consent process—including literacy-appropriate language, culturally adapted formats, verification checkpoints for ongoing consent, and engagement strategies with both staff and incarcerated populations—we also formalized the structure. The revised section now clearly outlines the objective of each recommendation, the practical steps for implementation, and explicitly uses PDL narratives to anchor and strengthen the evidence base for each suggested action.

Recommendations for the ethical inclusion of People Deprived of Liberty (PDL) in clinical trials of new tuberculosis vaccines are presented in **lines 371–465.**

**Question 3. The manuscript notes that some individuals refused participation but does not explore or report their reasons. In a study fundamentally concerned with autonomy and coercion in a carceral setting, understanding the perspectives of those who declined is critical. Being transparent about their reasons would provide a more complete understanding of the landscape of opinion and the potential barriers to ethical recruitment.**

Response:

The inclusion of perspectives regarding refusal to participate is addressed through enhanced transparency in the Methods section under 'Participant Recruitment and Eligibility Criteria.' We explicitly detail that two individuals declined participation upon arrival at the focus group setting and, crucially, that their reasons for refusal were intentionally not collected. This decision aligns with our ethical commitment to strictly respect participant autonomy, providing a complete picture of the non-coercive recruitment dynamics.

***Participant Recruitment and Eligibility Criteria (****line 145 a 153)*

*The total of 93 individuals were initially approached across all sites. Upon arrival at the FG setting, the research team explained the study objectives, procedures, potential risks and benefits, and emphasized voluntariness. Individuals were explicitly asked whether they wished to stay or return to their cells without any consequences. Two individuals declined participation at this stage, choosing to return to their cells. No reasons for refusal were collected, in line with the ethical commitment to respect autonomy. No participants withdrew after signing informed consent.*

**Question 4. Similarly, a more explicit and reflexive discussion of researcher positionality is required. The power differential between academic researchers and incarcerated participants is the central context of this work. The manuscript would be strengthened by a critical examination of how the researchers' social identities, institutional affiliations, and preconceptions may have influenced focus group facilitation, participant responses, and data interpretation.**

Response:

We added a dedicated paragraph in the Discussion **(line 556 to 574),** addressing the research team’s positionality, institutional affiliations, and potential influence on data collection and interpretation. The revised section discusses how reflexive practices were integrated to mitigate power differentials and acknowledges the inherent power asymmetry in research involving People Deprived of Liberty (PDL).

We recognize that the institutional affiliations, social identities, and personal beliefs of the researchers—particularly the facilitators—may have shaped participant responses during focus groups. To minimize such effects, facilitators with extensive experience in qualitative research and prison-based studies carefully framed questions to avoid leading language or value-laden judgments. They also intentionally probed for divergent views and emphasized confidentiality to foster open, authentic discussion.

Despite these efforts, we acknowledge that our findings may still have been influenced by social desirability bias, as participants might have felt compelled to align their responses with perceived expectations of facilitators or peers. The group setting may also have limited the expression of sensitive experiences, such as coercion, distrust, or pressure from prison authorities, gang structures, or fellow PDL. In highly stratified prison environments, existing hierarchies and social dynamics can shape the willingness to voice dissenting or critical perspectives on participation in vaccine research.

**Major comments**

**Question 5. One thing that appears conspicuous is the absence of discordant perspectives, or any discussion of dissent, disagreement, or negative cases. Were there really no participants who expressed cynicism or distrust? The results present a consensus, supported by illustrative quotes, with no outliers that challenge the manuscripts narrative. If there were participants who expressed strong opposition, or distrust regarding research participation, these perspectives should be integrated into the analysis. Their absence or omission risks presenting an overly consensual and potentially incomplete picture.**

Response:

We thank the reviewer for this important comment. In response, we revised the Results and Discussion to explicitly include both supportive and skeptical views about research participation. Several participants expressed willingness to contribute to scientific progress and protect others, provided they received clear information and safety assurances.

Conversely, others voiced distrust, citing fears of being used as “guinea pigs,” skepticism toward vaccine efficacy, and prior experiences of coercion or neglect during COVID-19 vaccination. We also note that participants expressed criticism toward the quality of prison health care, including perceived negligence by prison staff and dissatisfaction with previous vaccination campaigns, which were likely administered by correctional health services. Interestingly, participants appeared to differentiate external research teams from prison health personnel, expressing comparatively greater trust in the former. This distinction may have shaped their positive attitudes toward hypothetical TB vaccine research led by independent investigators.

To address this issue more comprehensively, we incorporated the following analytical paragraph into the Discussion section (**lines 508-520**):

“The coexistence of enthusiasm and skepticism toward research participation underscores the ambivalent nature of trust within carceral settings. While many participants recognized the social and scientific value of clinical research, others interpreted it through a lens of vulnerability and historical neglect. Distrust was not absolute but conditional—shaped by the degree of transparency, voluntariness, and external oversight. In particular, participants tended to express greater trust in external research teams than in prison health authorities, likely reflecting previous experiences of neglect or coercion in routine health care and vaccination campaigns. This distinction has important ethical implications: ensuring that vaccine trials are clearly led by independent research teams, rather than correctional staff, may enhance their perceived legitimacy and acceptability. Moreover, fears of being ‘used as test subjects’ reveal both limited opportunities for health education and legitimate concerns about power imbalances and inequity. Addressing these perceptions through participatory communication, trustworthy partnerships, and transparent ethical engagement is essential to fostering genuine autonomy and informed consent in future vaccine research involving PDL.”

**Question 6:** **On a related note, the use of convenience sampling is mentioned almost in passing, and requires elaboration. The manuscript does not state how many individuals initially volunteered, nor whether any were excluded after volunteering. How were participants actually selected? Did everyone have the opportunity to volunteer? Were they pre-vetted? Were they considered the more compliant, less fearful, or more privileged individuals within the prison hierarchy? The potential for selection bias here is substantial and fundamentally shapes who gets to have a "perspective" in this study. Those who volunteered may represent a specific subset of the population with shared traits or living conditions not representative of the general population. In a multi-site study across diverse prisons, not providing more detail here is a unignorable weakness.**

We thank the reviewer for bringing attention to the need for greater detail around procedures for participant recruitment and selection, and for discussion around the limitations of our approach.

We expanded the Methods to describe the recruitment process in detail **(145 a 168),** including the number of individuals volunteered and excluded; the role of prison social service teams; and potential sources of selection bias. We critically discuss these limitations in the Discussion, acknowledging their impact on representativeness **(lines 546 a 555)**.

*Participant Recruitment and Eligibility Criteria*

Participants (N = 91) were recruited through a multi-stage convenience sampling strategy, designed to ensure inclusion of both male and female incarcerated individuals from diverse cells and pavilions across participating prisons. Prior to recruitment, research teams from the participating center conducted visits to each participating prison to (i) present the study to prison administrators and health/social service staff, (ii) identify appropriate spaces for focus group discussions, and (iii) discuss eligibility criteria and logistical considerations with prison social service teams.

Given the security restrictions within prison settings, potential participants were initially identified by the prison social service sector in each unit. Identification followed the predefined eligibility criteria, with a special emphasis on ensuring representativeness, but was also subject to operational considerations (e.g., movement schedules, security clearance). Eligibility criteria included: (1) age 18 years or older; (2) literacy; (3) a cumulative incarceration period of at least 12 months (continuous or non-continuous); and (4) provision of voluntary informed consent.

A total of 93 individuals were initially approached across all sites. Upon arrival at the focus group setting, the research team explained the study objectives, procedures, potential risks and benefits, and emphasized voluntariness. The Free and Informed Consent Form (Termo de Consentimento Livre e Esclarecido – TCLE) was read aloud and discussed with the PDL. Individuals were explicitly asked whether they wished to stay or return to their cells without any consequences. Two individuals declined participation at this stage, choosing to return to their cells. No reasons for refusal were provided. No participants withdrew after signing informed consent. Those who agreed to participate signed two copies of the form—one was kept by the participant or filed in the medical record.

Discussion

We also acknowledge the critical power asymmetry inherent in research involving PDL. The institutional affiliations, social identities, and beliefs of the researchers, especially the facilitators, may have influenced participant responses during focus groups. To mitigate this, facilitators with demonstrated expertise in this methodology posed questions carefully, aiming to avoid guiding questions or value judgments that may have influenced responses. Moreover, facilitators intentionally probed for divergent beliefs and provided strong assurances of confidentiality to encourage sharing of honest, authentic perspectives. Despite these attempts, our findings may have still been subject to social desirability bias, as participants might have felt compelled to offer responses that aligned with perceived expectations of facilitators or peers. The group setting may also have constrained open discussion on sensitive topics, including potential experiences of coercion, distrust, or pressure from prison authorities, gang dynamics, or other PDL. In highly stratified prison environments, group hierarchies and social tensions may influence the expression of dissent, fear, or more critical perspectives on participation in vaccine trials. Future studies could consider using complementary methodologies—such as anonymous surveys or in-depth individual interviews—to elicit more candid responses and triangulate findings across formats.

**Responses to Reviewer #2**

We thank you for your positive assessment and helpful suggestions.

**Question 1: Data availability: While confidentiality concerns are valid, consider providing an anonymized coded dataset or expanded supplementary material (e.g., summary matrices) to strengthen compliance with PLOS’s data policy.**

Response:

We thank the reviewer for this valuable suggestion. In response, we developed and included three *Supporting Information* tables to enhance transparency and ensure alignment with PLOS’s data policy while safeguarding participant confidentiality. Specifically, we constructed **two tables summarizing the anonymized and aggregated qualitative data** and **one table presenting the guiding questions used during the focus group discussions**. All tables are now cited in the manuscript and listed after the References section with full legends.

**Table S1:** Distribution of Narratives by Core Theme, Subcategory, State, and Prison Type
**Table S2:** Matrix of Narrative Summary: Convergence and Divergence by Core Theme
**Table S3:** Guiding Questions for Focus Group Discussions on Health Care, Tuberculosis, and

Acceptability of a New TB Vaccine among Incarcerated Individuals

These materials provide structured, de-identified summaries that allow readers to assess the analytic process and thematic coherence of the study while maintaining ethical standards and the privacy of participants.

**Question 2: Quotations: Some direct quotations are lengthy or repetitive; consider condensing for readability without losing participants’ perspectives.**

We edited several participant quotations to remove repetitions and condense lengthy passages, maintaining the integrity of participants’ perspectives while improving readability.

**Question 3: References: A few references are incomplete (e.g., Ako 2020; Furin, Cox, Pai 2019) and should be corrected/standardized.**

We reviewed and updated all references in the manuscript to ensure accuracy and consistency. Missing entries (e.g., Ako 2020; Furin, Cox, and Pai 2019) were completed, and formatting was standardized according to PLOS style guidelines. We also incorporated additional citations suggested by Reviewer #1 and relevant to our study context. No retracted or unreliable sources have been cited.

Specifically, we added five new references to strengthen the discussion:

**36.** Legrand NM, Bull RA, Hajarizadeh B, Lloyd AR, Johnston K, Issa K, et al. Surveillance of Viral Respiratory Infections within Maximum-Security Prison, Australia. Emerg Infect Dis. 2025;31. doi:[10.3201/eid3108.240571](https://doi.org/10.3201/eid3108.240571)2.

**37.** Talevski J, Wong Shee A, Rasmussen B, Kemp G, Beauchamp A. *Teach-back: A systematic review of implementation and impacts.* Mathes T, editor. *PLoS ONE.* 2020;15:e0231350. doi:10.1371/journal.pone.0231350

**38.** Andrews JR, Charalambous S, Churchyard G, Cobelens F, Fernández-Escobar C, Frick M, et al. *The participation of people deprived of liberty in tuberculosis vaccine trials: should they be protected from research, or through research?* *The Lancet Infectious Diseases.* 2025; S1473-3099(25)00305-6. doi:10.1016/S1473-3099(25)00305-6

**39.** Moazen B, Ismail N, Agbaria N, Mazzilli S, Petri D, Amaya A, D'Arcy J, Plugge E, Tavoschi L, Stöver H. *Vaccination against emerging and reemerging infectious diseases in places of detention: a global multistage scoping review.* *Front Public Health.* 2024 Jan 29;12:1323195. doi:10.3389/fpubh.2024.1323195

**40.** Chin ET, Leidner D, Zhang Y, Long E, Prince L, Li Y, et al. *Effectiveness of the mRNA-1273 Vaccine during a SARS-CoV-2 Delta Outbreak in a Prison.* *New England Journal of Medicine.* 2021;385(24):2300-2301. doi:10.1056/NEJMc2114089

**Question 4. Clarity in Results: In one passage, the phrase “It’s mandatory to take it” is ambiguous (COVID-19 vaccine vs. TB trial participation). Clarify context.**

Response:

We clarified the Results section to specify that the quote refers to the COVID-19 vaccination campaign within prisons, not participation in TB vaccine trials.

**Conclusion**

We believe these revisions have strengthened the manuscript substantially. We have clarified methodological details, enriched the discussion with empirical evidence and reflexivity, improved data availability, and aligned fully with PLOS formatting and ethical requirements.

We thank the Editor and Reviewers again for their constructive input and look forward to your assessment of our revised manuscript.

Sincerely,
